# Hologenomic insights into mammalian adaptations to myrmecophagy

**DOI:** 10.1093/nsr/nwac174

**Published:** 2022-08-24

**Authors:** Shao-Chen Cheng, Chun-Bing Liu, Xue-Qin Yao, Jing-Yang Hu, Ting-Ting Yin, Burton K Lim, Wu Chen, Guo-Dong Wang, Cheng-Lin Zhang, David M Irwin, Zhi-Gang Zhang, Ya-Ping Zhang, Li Yu

**Affiliations:** State Key Laboratory for Conservation and Utilization of Bio-Resources in Yunnan, School of Life Sciences, Yunnan University, Kunming 650091, China; State Key Laboratory for Conservation and Utilization of Bio-Resources in Yunnan, School of Life Sciences, Yunnan University, Kunming 650091, China; State Key Laboratory for Conservation and Utilization of Bio-Resources in Yunnan, School of Life Sciences, Yunnan University, Kunming 650091, China; State Key Laboratory for Conservation and Utilization of Bio-Resources in Yunnan, School of Life Sciences, Yunnan University, Kunming 650091, China; State Key Laboratory of Genetic Resources and Evolution, Kunming Institute of Zoology, Chinese Academy of Sciences, Kunming 650223, China; Department of Natural History, Royal Ontario Museum, Toronto, ON M5S2C6, Canada; Guangzhou Zoo, Guangzhou 510000, China; State Key Laboratory of Genetic Resources and Evolution, Kunming Institute of Zoology, Chinese Academy of Sciences, Kunming 650223, China; Center for Excellence in Animal Evolution and Genetics, Kunming Institute of Zoology, Chinese Academy of Sciences, Kunming 650223, China; Beijing Zoo, Beijing 100000, China; Department of Laboratory Medicine and Pathobiology, University of Toronto, Toronto, ON M5S2E8, Canada; State Key Laboratory for Conservation and Utilization of Bio-Resources in Yunnan, School of Life Sciences, Yunnan University, Kunming 650091, China; State Key Laboratory for Conservation and Utilization of Bio-Resources in Yunnan, School of Life Sciences, Yunnan University, Kunming 650091, China; State Key Laboratory of Genetic Resources and Evolution, Kunming Institute of Zoology, Chinese Academy of Sciences, Kunming 650223, China; Center for Excellence in Animal Evolution and Genetics, Kunming Institute of Zoology, Chinese Academy of Sciences, Kunming 650223, China; State Key Laboratory for Conservation and Utilization of Bio-Resources in Yunnan, School of Life Sciences, Yunnan University, Kunming 650091, China

**Keywords:** myrmecophagy, adaptive evolution, hologenome, genomics, metagenomics, convergent evolution

## Abstract

Highly specialized myrmecophagy (ant- and termite-eating) has independently evolved multiple times in species of various mammalian orders and represents a textbook example of phenotypic evolutionary convergence. We explored the mechanisms involved in this unique dietary adaptation and convergence through multi-omic analyses, including analyses of host genomes and transcriptomes, as well as gut metagenomes, in combination with validating assays of key enzymes’ activities, in the species of three mammalian orders (anteaters, echidnas and pangolins of the orders Xenarthra, Monotremata and Pholidota, respectively) and their relatives. We demonstrate the complex and diverse interactions between hosts and their symbiotic microbiota that have provided adaptive solutions for nutritional and detoxification challenges associated with high levels of protein and lipid metabolisms, trehalose degradation, and toxic substance detoxification. Interestingly, we also reveal their spatially complementary cooperation involved in degradation of ants’ and termites’ chitin exoskeletons. This study contributes new insights into the dietary evolution of mammals and the mechanisms involved in the coordination of physiological functions by animal hosts and their gut commensals.

## INTRODUCTION

Diet profoundly affects mammalian radiations into new ecological niches. In addition to well-known carnivory, herbivory and omnivory, highly specialized myrmecophagy (ant- and termite-eating) independently evolved multiple times in species of various mammalian orders (e.g. anteaters and armadillos of Xenarthra, echidnas of Monotremata, pangolins of Pholidota, numbats of Marsupialia, aardvarks of Tubulidentata and aardwolfs of Carnivora) [1]. This represents a textbook example of phenotypic evolutionary convergence. Over 90% of myrmecophagous mammals’ diets consist of ants and termites [2–4], which have high contents of proteins and lipids (they respectively account for 40%–70% and 14%–26% of their total body weight) and trehalose—the primary insect blood sugar [2,5–7]. In addition, myrmecophagous mammals are unique in that they can digest the chitinous exoskeleton that covers the whole body of an ant or termite to obtain nutrients and energy, and can detoxify the toxic substances (e.g. formic acid and alkaloids) that the ants/termites secrete [7–13].

The long-term dietary specialization involved has driven myrmecophagous mammals’ evolution of various morphological and physiological adaptations. These include: strong forelimbs with long claws that facilitate excavation of ant nests; the regression or loss of teeth, a tubular mouth, well-developed salivary glands and a slender tongue that enable the capture and ingestion of large numbers of ants or termites; a cornified interior stomach and thickened pylorus muscle layers that facilitate digestion of hard chitinous exoskeletons; and higher trehalase activities in the small intestine than those of non-myrmecophagous mammals, which enable trehalose digestion [1,14–17]. However, the genetic mechanisms that contribute to the dietary convergence of myrmecophagous mammals, especially those involved in the digestion of various nutrients and defenses against toxic secretions, remain unclear.

Many studies have shown that long-term dietary adaptations are associated with genetic changes in both host genomes and host-associated microbiomes. Examples of the adaptive changes in host genomes include amylase gene duplications during the domestication process that led to the evolution of dogs (with starch-rich diets) from carnivorous wolves [18]. Another is the adaptive convergence in giant and red pandas’ genomes of the positive selection of three genes (*PRSS1, PRSS36* and *CPB1*) involved in dietary protein digestion that improve the efficiency of nutrient absorption and utilization from low-nutrient and high-fiber bamboo-based diets [19]. Examples of adaptive changes in symbiotic microbes include findings, in studies of human gut microbiota, that show that people with high-protein and high-fat diets generally have a Bacteroides-dominated enterotype while those with carbohydrate-rich diets generally have a Prevotella-dominated enterotype [20,21]. Another is a recent finding that serine endopeptidases and aminopeptidases identified in the gut microbiota of vultures can help the host to detoxify toxins (e.g. shiga and tetanus toxins) in carrion [22]. In addition, convergent evolution in structure and function of the gut microbiota of yaks and Tibetan sheep helped their hosts to produce more energy-providing substances (short-chain fatty acids) in adaptive responses to extreme high-altitude environments [23].

Increasing numbers of studies have also shown that interactions between host genomes and host-associated microbiomes, i.e. ‘hologenomes’ [24,25], play important roles in mammalian dietary specializations. Examples include the suite of changes involved in vampire bats’ adaptation to a diet of blood that is risky (due to blood-borne pathogens) and has poor nutrient balance (with high protein and low carbohydrate contents). An important adaptation was positive selection of a glucose homeostasis-related gene (*FFAR1*) that helped the bat hosts to maximize use of carbohydrates. Others included enrichment of gut microbiota with urea metabolism-related genes (e.g. *ureA*) and potentially protective bacteria (e.g. *Amycolatopsis*) that enhance hosts’ metabolism of urea and other nitrogen-based wastes, and improve their resistance to blood-borne pathogens [26].

The myrmecophagy that has evolved in multiple mammalian orders provides an ideal model for hologenomic studies to elucidate the genetic basis of evolutionarily convergent dietary adaptations and host-microbiota interactions. Previous studies have addressed host or microbial components of the genetic basis of myrmecophagous adaptation in one myrmecophagous group of mammals, pangolins [27–30]. Others have characterized dietary convergence of myrmecophagous mammals based solely on microbial composition inferred from *16S rRNA* analyses [1]. However, there have been no systematic studies of the convergence of host and microbiome genes and functions, i.e. convergence at the hologenome level, yet.

In the present study, we elucidate the hologenomic mechanisms involved in the dietary convergence of myrmecophagous mammals more comprehensively, through multi-omics analyses. These included analyses of host genomes and transcriptomes, as well as gut metagenomes, in combination with validating assays of key enzymes’ activities, in the species of three mammalian orders (anteaters, echidnas and pangolins of the orders Xenarthra, Monotremata and Pholidota, respectively) and their relatives. We demonstrate the complex and diverse interactions between hosts and their symbiotic microbiota that have provided adaptive solutions for nutritional and detoxification challenges associated with high levels of protein and lipid metabolisms, trehalose degradation and toxic substance detoxification. Interestingly, we also reveal their spatially complementary cooperation involved in degradation of ants’ and termites’ chitin exoskeletons. This study contributes new insights into the dietary evolution of mammals and the mechanisms involved in the coordination of physiological functions by animal hosts and their gut commensals.

## RESULTS

### New *de novo* genome assemblies and comparative genomic analysis

We obtained high-quality *de novo* genome assemblies for the lesser anteater (*Tamandua tetradactyla*) and short-beaked echidna (*Tachygolssus aculeatus*) using the SMRT long-read sequencing and whole-genome shotgun (WGS) short-read sequencing strategies (Supplementary Table S1). We also downloaded a publicly available genome of the Malayan pangolin (*Manis javanica*), and genomes of some of the three species’ relatives (Supplementary Table S2), to generate comparative genomic data. The sizes of the anteater and echidna genome assemblies were 3.05 and 2.37 Gb, with contig N50 values of 3.4 and 8.76 Mb, respectively (Supplementary Table S1). The quality of the *de novo* genome assemblies and annotations was assessed using the core eukaryotic genes mapping approach (CEGMA) and benchmarking universal Single-copy orthologs (BUSCO). CEGMA evaluation of the anteater and echidna genome assemblies indicated that they included complete copies of 234 (94.35%) and 233 (93.95%) out of 248 core eukaryotic genes. Similarly, BUSCO evaluation showed that they included complete copies of 87.8% and 91.1% of 4104 single-copy mammalian orthologous genes (Supplementary Table S1). These evaluations show that the genome assemblies had high completeness. In addition, 98.74% of the Illumina anteater reads and 98.94% of the echidna reads could be mapped to their respective assembled genomes (Supplementary Table S1). The base error rate of the whole genome was respectively 0.001% and 0.006% for anteater and echidna (Supplementary Table S1), indicating that the genome assemblies had high accuracy.

**Figure 1.**
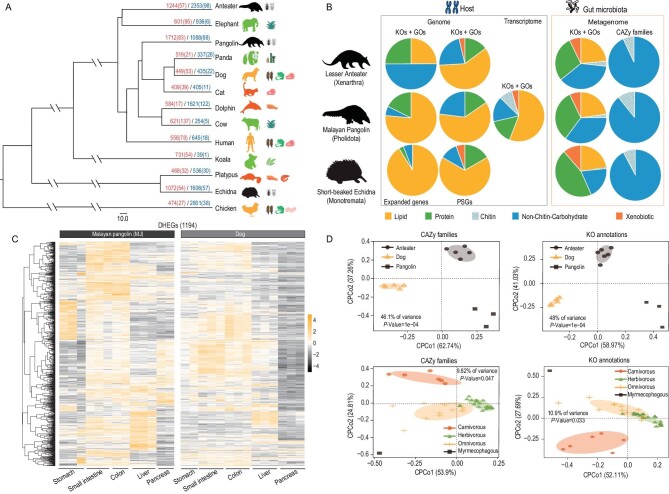
Summary of comparative host genomic, transcriptomic and gut metagenomic analyses of myrmecophagous mammals and mammals of other dietary classes. (A) Results of analysis of gene families in 13 species examined in this study. Numbers on branches indicate numbers of genes of families that have apparently expanded and contracted, with numbers of gene families that have significantly expanded (in red) and contracted (in blue) in parentheses. The colors of species’ images indicate their feeding habits (black, green, orange and red for myrmecophagous, herbivorous, omnivorous and carnivorous species, respectively). (B) Pie charts summarizing significant functional enrichment related to nutrition (lipid, protein, carbohydrate and chitin degradation) and detoxification for myrmecophagous hosts and their gut microbiomes. The pie chart shows the total number of KEGG Orthology (KOs)/GO terms/CAZy families, and the numbers of various nutritional categories and detoxification occupied in the total number of KOs/GO terms/CAZy families. The myrmecophagous host pie charts include the number of KOs and GO terms from the functional enrichment analyses of gene family expansion (Supplementary Table S3), PSGs (Supplementary Table S4) and highly expressed genes (DHEGs) (Supplementary Table S8) in myrmecophagous species compared to non-myrmecophagous species. Their gut microbiome pie charts include the number of KOs, GO terms and CAZy families from the functional enrichment analyses of the higher abundance of classifications in myrmecophagous species compared to non-myrmecophagous species (Supplementary Table S13). (C) Heat maps of 1194 DHEGs that were more highly expressed in Malayan pangolins (in tissues related to food digestion and absorption) than in dogs. (D) Upper left and upper right panels respectively show plots obtained from analyses of CAZy families and KO annotations of the pangolin, anteater and dog. Lower left and lower right panels respectively show plots obtained from analyses of CAZy families and KO annotations of the echidna and 38 other mammals.

Gene family analyses (Fig. 1A) and positive selection analyses revealed that the significantly expanded genes and positively selected genes (PSGs) of the three myrmecophagous species (lesser anteater, short-beaked echidna and Malayan pangolin) were significantly enriched in categories involved in carbohydrate, protein and lipid metabolism (Fig. 1B, Supplementary Tables S3 and S4). Intriguingly, enrichment of some GO terms and pathways was shared by pairs of the myrmecophagous species (Supplementary Table S5). Gene family analyses showed that GO: 0015031 (‘protein transport’) was enriched in both the anteater and echidna. In addition, positive selection analyses showed that several terms involved in metabolism of lipids (GO: 0000038, ‘very-long-chain fatty acid metabolic process’), proteins (GO: 0005782, ‘peroxisomal matrix’ and GO: 0006625, ‘protein targeting to peroxisome’) and carbohydrates (GO: 0008286, ‘insulin receptor signaling pathway’) were enriched in both the anteater and echidna. A term involved in lipid metabolism (GO: 0005777, ‘peroxisome’) was enriched in the pangolin and echidna. Moreover, some PSGs related to metabolism of these substances were also shared by all or pairs of the myrmecophagous species. The gene *MVK* [31], involved in cholesterol biosynthesis, was found among PSGs of all three of the species. A gene involved in cholesterol catabolism (*CYP7A1*) [32,33] was identified as a PSG in the anteater and pangolin, while *HSD17B4*, involved in oxidation of fatty acids [34], was identified as a PSG in the pangolin and echidna. Four PSGs associated with metabolism of lipids (*HACL1, SCARF1*) [35,36] and proteins (*MCCC1, IDE*) [37,38] were shared by the anteater and echidna (Supplementary Table S5).

We next identified amino acid substitutions in proteins shared by the three myrmecophagous species (Supplementary Table S5). Overall, 36 convergent/parallel amino acid substitutions in proteins encoded by 34 genes were observed in all three species. This number is significantly higher than chance (*P* < 0.05), and is further verified by a significantly low level of convergence along the combined branches of the non-myrmecophagous sister species (dog, cat, giant panda, elephant and platypus) (16 convergence/parallel amino acid substitutions in proteins encoded by 16 genes; *P* < 0.05) to these three myrmecophagous species. Among the 34 genes identified in myrmecophagous species, the beta-hexosaminidase subunit alpha gene (*HEXA*) participates in producing N-Acetylglucosamine (GlcNAc), which is the main degradation product of chitin [39]. Another is the LDL Receptor Related Protein 1 gene (*LRP1*), encoding a member of the low-density lipoprotein receptor family, which is involved in maintenance of normal cholesterol homeostasis [40]. Besides those identified in all three myrmecophagous species, 863 convergent/parallel amino acid substitutions in proteins encoded by 606 genes were observed in the anteater and pangolin; 210 in proteins encoded by 161 genes in the anteater and echidna; and 153 in proteins encoded by 140 genes in the pangolin and echidna. This number is significantly higher than chance (*P* < 0.05). These genes also showed significant enrichment in the categories involved in carbohydrate, protein and lipid metabolisms (Supplementary Table S6). Interestingly, there were several overlapping GO terms and KEGG pathways in the pairwise comparisons (Supplementary Table S5). In addition, *SCARF1*, a lipid metabolism-related gene found in the anteater and echidna [36], was also identified as a PSG in these two species (Supplementary Table S5).

We also examined two enzymes with crucial roles in the digestion of chitin and trehalose, both of which are abundant in ants and termites. One, acidic mammalian chitinase (AMCase), encoded by the chitinase gene (*CHIA*), is responsible for the digestion of the exoskeletal chitin of insects [41], and the species that consume invertebrates have multiple functional copies of this gene [42,43]. We found four functional copies of *CHIA* in the lesser anteater genome, and one each in genomes of the Malayan pangolin and short-beaked echidna (Supplementary Fig. S1). The presence of multiple functional *CHIA* paralogs in the anteater indicates that the adaptive changes during the species’ evolution included increases in AMCase enzyme levels and hence capacity to digest chitin [42,43]. In contrast, the single functional *CHIA* in pangolin and echidna indicates that

alternative mechanisms may be involved in chitin degradation in these two species, such as higher levels of the gene's expression and/or participation of chitin-digesting bacteria [42]. The other examined enzyme, trehalase, encoded by the trehalase gene (*TREH*), catalyzes conversion of trehalose to glucose [44]. Retention of this gene has been found to correlate closely with the feeding habits of insectivorous mammals, while loss or pseudogenization of the gene correlates with habits of non-insectivorous mammals [45]. We found that the three myrmecophagous species all have an intact *TREH* gene (Supplementary Fig. S2), corroborating their capacity to metabolize trehalose.

### Transcriptome assemblies and differential gene expression analysis

To supplement the genetic information acquired regarding the host genomes, we compared transcriptomes of the myrmecophagous Malayan pangolin (*M. javanica*) and its closest relative, the omnivorous dog (*Canis familiaris*). For this we obtained high-quality RNA-seq data on transcriptomes of 10 tissues, including 5 associated with food digestion and absorption (stomach, small intestine, colon, liver and pancreas) and 5 others (lung, cerebrum, tongue, kidney and heart), of three pangolins (designated as Mp01T, Mp02T and Mp03T) and three dogs (designated as D01T-D03T) (Supplementary Fig. S3, Supplementary Table S7). A significant difference between dogs and pangolins is that the oxyntic glands of dogs are scattered within the gastric tissue, whereas those of pangolins are concentrated in a highly specialized glandular mass, forming an oval mound elevated above the gastric lumen [12,29]. Thus, in addition to collecting normal gastric tissue (from Mp01T and Mp02T) as in dogs, we also collected samples of the specialized oxyntic gland tissue from the pangolin (Mp03T). In total, 21 785 and 36 391 transcripts were reconstructed from the pangolin and dog transcriptomic data sets, respectively.

Of the differentially highly expressed genes (DHEGs), we identified, from analyses of tissues involved in the digestion and absorption of food, 1194 that had higher expression levels in Malayan pangolins than in dogs (Fig. 1C). In accordance with results of the genomic analyses, significant enrichment of categories involved in carbohydrate, protein and lipid metabolisms was detected in these DHEGs (Fig. 1B, Supplementary Table S8), except those from the pancreas, which showed significant enrichment in the immune response category. Interestingly, we also detected significant enrichment of an arachidonic acid metabolism pathway (ko00590), which participates in lipid metabolism, in analyses of pangolin gene families (Supplementary Table S3). Four GO terms and three pathways related to lipid and protein metabolism were also significantly enriched in the PSGs of pangolins (Supplementary Table S4), as was a lipid metabolism-related GO term (peroxisome, GO : 0005777) in the PSGs of both the pangolin and echidna (Supplementary Table S5).

**Figure 2.**
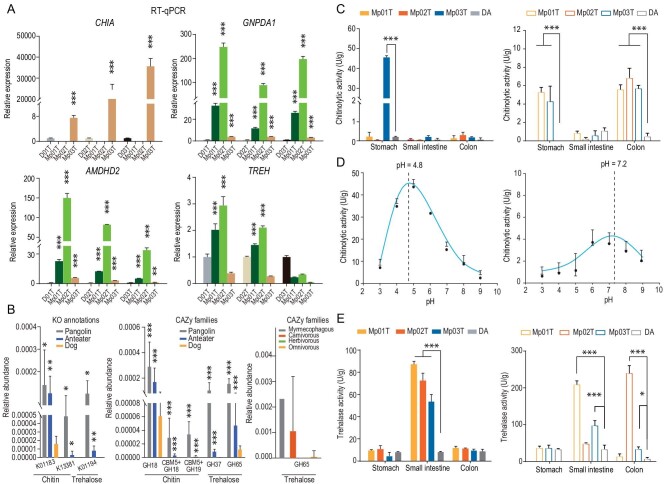
Host and gut microbiome expression of dietary chitin- and trehalose-degradation genes and enzyme (chitinase and trehalase) activities. (A) Results of quantitative reverse transcription PCR (RT-qPCR) analysis of four genes involved in diet-related chitin (*CHIA, AMDHD2* and *GNPDA1*) and trehalose (*TREH*) metabolism in pangolins and dogs. CHIA is expressed at a significantly higher level in the oxyntic gland tissue of pangolins than in the stomach of dogs (upper left) and other pangolin tissues (Supplementary Fig. S4A). AMDHD2 (lower left), GNPDA1 (upper right) and TREH (lower right) are expressed at a significantly higher level in the intestine of pangolins than the intestine of dogs and other pangolin tissues (Supplementary Fig. S4C, E and G). (B) KO annotations and CAZy families associated with the degradation of chitin and trehalose are significantly enriched in pangolins and anteaters, and more enriched in echidnas relative to mammals with other diets. (C) Chitinolytic activities of tissues (left) and contents (right) of the gastrointestinal (GI) tract of pangolins and a dog. No chitinolytic activity was detected in the gastric content of DA. Gastric content samples of Mp02T were not successfully obtained. (D) Chitinolytic activity in the oxyntic gland (left) and colon contents (right) measured at different pH settings in pangolins. In each case the intersection point of the dashed line and X-axis is the optimum pH. (E) Trehalase activities of tissues (left) and contents (right) of GI tracts of pangolins and a dog. Mann-Whitney *U* tests were used to assess the significance of differences between samples (*P < 0.05, **P < 0.01, ***P < 0.001).

Notably, enrichment of multiple chitin degradation-related GO terms and pathways was observed (Fig. 1B, Supplementary Table S8). *CHIA* was expressed at significantly higher levels in the oxyntic gland tissue of the pangolin than in both other pangolin tissues and dog stomach. Thus, although there is only one functional copy of *CHIA* in the pangolin genome, its increased expression may play a key role in chitin digestion. In addition, *AMDHD2* and *GNPDA1*, which are involved in the breakdown of chitin into glucose [46], were both significantly more highly expressed in the small intestine of pangolins than in the small intestine of dogs and other pangolin tissues. *TREH* was also significantly more highly expressed in the small intestine of the two pangolins Mp01T and Mp02T than in the small intestine of dogs and other pangolin tissues. The higher expression of the four DHEGs involved in degradation of chitin (*CHIA, AMDHD2* and *GNPDA1*) and trehalose (*TREH*) was verified by quantitative reverse transcription PCR (RT-qPCR) analysis (Fig. 2A, Supplementary Fig. S4).

In addition, several GO terms and pathways related to the metabolism and detoxification of xenobiotics, including the exogenous drug catabolic process (GO: 0042738) and drug metabolism—cytochrome P450 (ko00982), were significantly enriched in the liver and small intestine (Supplementary Table S8). Glucose dehydrogenase (*UGDH*) and UDP-glucuronosyltransferases (UGTs: *UGT1A10* and *UGT2A3*), which are important families of detoxification enzymes and catalyze the conjugation of diverse exogenous compounds [27,47–50], were also significantly more highly expressed in the pangolin small intestine than in the small intestine of dogs. *UGT1A10* was also significantly more highly expressed in the liver of pangolins than in dogs.

### Metagenome assemblies and analyses

In addition to the host genomic and transcriptomic analyses, we also analyzed gut microbiomes. Metagenomic data from three Malayan pangolins (*M. javanica*) and six giant anteaters (*Myrmecophaga tridactyla*) representing anteaters were obtained and compared with those from five dogs (Supplementary Table S9). Filtered reads from the same species were assembled by individual and co-assembly strategies, resulting in 283 710, 1 696 545 and 600 142 contigs for pangolin, anteater and dog metagenomes, and 751 768, 4 188 947 and 1 672 532 open reading frames (ORFs), respectively (Supplementary Table S9). In addition to pangolin and anteater, we analyzed metagenomes of the short-beaked echidna and 38 other mammals comprising 11 omnivores, 21 herbivores and 6 carnivores, using previously presented data [51] (Supplementary Table S10).

Three myrmecophagous species showed higher or significantly higher proportions of their symbiotic bacteria that were associated with chitin degradation, secondary bile acid biosynthesis and short-chain fatty acids biosynthesis than mammals with other diets (Supplementary Tables S11 and S12). Constrained principal coordinate analysis (CPCoA) of 713 CAZy families and 8567 KEGG Orthology (KO) annotations obtained from analyses of the anteater, pangolin and dog samples clearly separated the anteater and pangolin samples from those of dog samples (Fig. 1D). Similarly, CPCoA of 509 CAZy families and 8356 KO annotations from analyses of the echidna and the non-myrmecophagous mammals confirmed the separation of the samples of echidna and other mammals (Fig. 1D), clearly indicating that the microbiomes of myrmecophagous mammals and mammals with other diets substantially differ.

We further investigated functional features of their microbiomes to explore reasons for the differences. We found that genes that were more abundant in microbiomes of the three myrmecophagous species than in those of other mammals were consistent with results of the host genomic and differential expression gene analyses. They were more enriched in categories involved in carbohydrate, protein and lipid metabolism, as well as the metabolism and detoxification of xenobiotics (Fig. 1B, Supplementary Table S13). In the carbohydrate metabolism category, KO annotations related to chitin degradation and trehalose degradation were mainly enriched, with chitinase (K01183, EC : 3.2.1.14), bifunctional chitinase/lysozyme (K13381, EC : 3.2.1.14 and EC : 3.2.1.17) and alpha, alpha-trehalase (K01194, EC : 3.2.1.28) significantly more abundant in pangolins and anteaters than in dogs (Fig. 2B). CAZymes of chitin degradation-related enzymes (GH18 and GH18 + CBM5, CBM5 + GH19) and trehalose degradation-related enzyme GH37 (trehalase) were significantly more abundant in pangolins and anteaters than in dogs (Fig. 2B). Moreover, trehalose degradation-related enzyme GH65 (trehalase) was more abundant in all three myrmecophagous species than in species with other diets (Fig. 2B).

### Chitinolytic and trehalase activity assays

Both the host genome and microbiome analyses provided evidence of adaptive changes in myrmecophagous mammals that facilitate chitin and trehalose degradation. To further explore the roles of host and gut microbes in degradation of these substances, we assayed chitinolytic and trehalase activities in host digestive tissues (oxyntic gland, stomach, small intestine and colon) and corresponding gut contents of three pangolins (Mp01T-Mp03T) and one dog (DA). The results show that the oxyntic gland and colon contents of pangolins have the highest chitinolytic activities (Fig. 2C, Supplementary Table S14). Relatively high chitinolytic activity was also detected in the gastric contents of the pangolins, but this might be due to chitinolase secreted from the pangolin's oxyntic gland, rather than microbial contributions. This hypothesis is supported by findings that the gastric and small intestine contents had lower levels of genes encoding chitinolytic-related enzymes than the colon content when we sequenced and analyzed metagenomes of pangolin gut contents (Supplementary Tables S15 and S16). In addition, the optimal pH values for chitinolytic activity in the pangolin's oxyntic gland tissue and colon contents were 4.8 and 7.2, respectively (Fig. 2D), similar to the average values (4.36 and 7.25) measured in the internal stomach and colon environments, respectively.

Trehalase activity was found to be significantly higher in the small intestines of all three pangolins than in the other pangolin tissues and all digestive tissues of the dog. We also detected higher trehalase activities in the small intestine contents of two of the three pangolins (Mp01T and Mp03T) than in the contents of other parts, or any gut contents of the dog (Fig. 2E, Supplementary Table S14). Thus, trehalase activities in both small intestine tissue and contents appear to be higher in pangolins than in dogs.

In summary, results of the enzyme activity assays corroborated the importance of both the host and gut microbes in chitin and trehalose degradation. Furthermore, the host and gut microbes contribute to chitin degradation in different parts of the digestive tract.

## DISCUSSION

**Figure 3.**
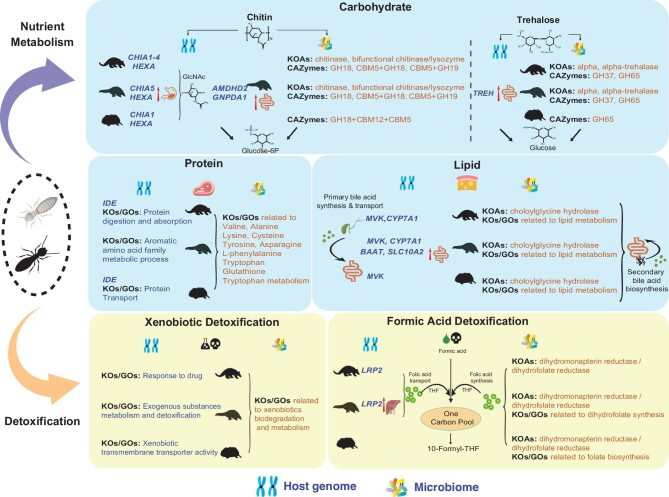
Hologenomic adaptation underlying mammalian myrmecophagous evolution. Blue boxes indicate the metabolism of nutrients, including carbohydrates (chitin and trehalose), proteins and lipids (particularly bile acid biosynthesis). Yellow boxes indicate xenobiotic and formic acid detoxification. Red arrows indicate genes that are differentially highly expressed in pangolins relative to dogs. KO annotations are abbreviated to KOAs. CAZy families are abbreviated to CAZys.

Studies of mammalian adaptations to myrmecophagy have lagged far behind those of animals with more common feeding habits. This study provides the first hologenomic insights into such myrmecophagous adaptations, and deciphers the complex and diverse interactions between hosts and the symbiotic gut microbiota involved in nutrient utilization and toxin resistance or tolerance (Fig. 3). These include not only synergistic cooperation in digestion of the high protein and lipid contents of the host diets, trehalose degradation, and detoxification of toxic substances, but also the spatially complementary cooperation involved in degradation of ants’ and termites’ chitin exoskeletons.

The first challenge for myrmecophagous mammals in obtaining nutrients from ants and termites is the ingestion and digestion of the hard chitin exoskeleton (Fig. 3). Our study shows that both the host genome and gut microbiome contribute to meeting the challenge of converting chitin into energy and nutrients. Myrmecophagous mammals have either higher numbers of functional paralogs of chitin degradation-related genes or higher gene expression and chitinolytic activity than other mammals. We also identified a higher abundance of chitin-digesting bacteria and enrichment of chitin degradation-related enzymes in the microbiomes of all three myrmecophagous mammals than in those of mammals with other diets. In previous studies, such adaptive changes facilitating chitin degradation in hosts and microbiomes have been observed in pangolins [28,29,52,53]. Our study extends these observations to several independently evolved myrmecophagous mammals. More interestingly, we found evidence that this interaction is complementary due to differences in locations of the host- and microbiome-mediated chitinolytic activity. By surveying the pangolins and dogs, the host activity is found to be highest in the oxyntic gland tissue of the pangolin stomach, while the gut microbiome activity is highest in the pangolin colon content. Little or no chitinolytic activity was detected in other pangolin digestive tissues and gut contents. A previous study also found that the chitinase gene is highly expressed in the oxyntic gland tissue of the pangolin stomach [29]. This is corroborated by our transcriptomic and RT-qPCR analyses, as well as our comparative analyses of pangolins and dogs. Furthermore, we observed high chitinolytic activity in this tissue. Insectivorous bats, which also have diets with high chitin content, have been reported to show chitinolytic activity only in intestine or gastric mucosa [54,55] or not found in both of them [56]. However, in the pangolins examined here, chitin is digested by a combination of endogenous chitinolytic enzymes from the oxyntic gland of the stomach and enzymes secreted by bacteria in the colon. We also found that the chitinolytic enzymes of pangolins, expressed in the stomach or by their colon microbiome, have adapted to the pH of the respective environments, thereby enabling more efficient chitin digestion. Thus, we propose that ‘spatially complementary co-action’ may be an important adaptive strategy of the polysaccharide metabolism of myrmecophagous mammals, involving both the host and gut microbes. This differs from the mechanism that has evolved in herbivores that heavily relies on the polysaccharide cellulose. Herbivores metabolize cellulose via bacteria in the stomach (foregut fermentation) or intestine (hindgut fermentation) [57–59], rather than adopting a cooperative strategy involving both host activities and gut microbes like the myrmecophagous mammals. It should be noted that this inferred strategy, based on the chitinolytic activity, is only found in pangolin species, and in the future this strategy needs to be verified by studies of other myrmecophagous species.

In addition to providing the host with nutrients and energy by digesting chitin itself, degradation of the chitin exoskeleton also releases protein, lipid and trehalose from ants’ and termites’ internal tissues (Fig. 3). Here again, host components and the microbiome of myrmecophagous mammals cooperatively extract nutrients from these sources. The *TREH* gene, which plays a key role in trehalose digestion, is expressed at a higher level and trehalase activity is higher in the small intestine of pangolins than in dogs. A previous proteomic analysis also detected TREH protein in the intestinal fluid of pangolins [30], and another previous study found higher trehalase activities in the small intestines of echidna than in those of humans or dogs [60]. Trehalase is also enriched in the microbiomes of all three myrmecophagous mammals relative to those of mammals with other diets. Altogether, these findings indicate that convergent evolution of myrmecophagous mammals has included adaptive increases in abilities to digest and utilize trehalose (Fig. 3).

We also detected interactive participation of the host genome and microbiome in protein and especially lipid metabolism (Fig. 3), which allows myrmecophagous mammals that prey on the lipid-rich larvae of ants and termites to exploit the high energy and nutrient contents of the larvae. Furthermore, genes and pathways particularly associated with the cholesterol metabolism pathways of lipid metabolism and bile acid biosynthesis were most significantly enriched. These findings are consistent with previous transcriptomic studies of pangolins [28,53]. We not only found that these pathways are enriched in multiple myrmecophagous mammals, but also identified several key related genes. For example, the primary synthesis of bile acids, which occurs in the host's liver from which the acids are released into the small intestine where they emulsify dietary fats, is controlled by genes that have either been positively selected (*CYP7A1*) [32,33] or up-regulated (*BAAT*) [33]. Following this, the primary bile acids are transported back into the liver via a process involving a transporter encoded by another up-regulated gene, *SLC10A2*, to promote the reabsorption of bile acids [61]. Bile acids that are not reabsorbed are transformed into unconjugated bile acids and secondary bile acids by the microbiome. Choloylglycine hydrolase (*BSH*) (K01442, EC 3.5.1.24), the key enzyme in the transformation of primary bile acids into unconjugated bile acids [62], is significantly more abundant in anteaters and pangolins than in dogs, and more abundant in echidna than 38 other mammals, albeit not significantly so (Fig. 3). Our metagenomic analysis also revealed that secondary bile acid synthesis pathways were more abundant in all three myrmecophagous species than in mammals with other diets, which has only been reported in the microbiome analyses of pangolins [52].

Insects can secrete various xenobiotics, including toxic substances [8,10–13]. Ants and termites are no exception, so myrmecophagous mammals must deal with detoxification challenges. Our analyses of host PSG analyses and DHEGs showed that GO functions and pathways involved in xenobiotic metabolism and detoxification were enriched in the small intestine and liver tissues of pangolins relative to dogs. They were also richer in the microbiomes of the three myrmecophagous mammals than in those of mammals with other diets (Fig. 3). Among them, the enrichment of xenobiotic metabolism, and degradation of benzoate, chloroalkane and chloroalkene degradation functions and pathways detected in the myrmecophagous mammals, have also been observed in previous metagenomic analyses of pangolins [52]. In addition, our results also demonstrate enrichment of GO functions and pathways associated with detoxification of secondary metabolites and toxic substances, as reported in studies of herbivorous mammals [8,12,63]. Notably, we found that the myrmecophagous mammals may have evolved the capacity to detoxify formic acid. Previous studies have found that formic acid is degraded by folic-acid-mediated one-carbon oxidation, and it enters the one-carbon pool by combination with tetrahydrofolic acid (THF), resulting in 10-formyl-THF formation and subsequent conversion to THF and CO_2_ [12,63–65]. We identified a key gene in folic acid transport, *LRP2* [66,67], under positive selection in our PSG analyses of anteater and pangolin genomes, and found that it is highly expressed in pangolin liver. Interestingly, the KO annotations from the microbiome analyses also found folic acid biosynthesis-related enrichment, with dihydromonapterin reductase/dihydrofolate reductase (*FolM*) (K13938, EC: 1.5.1.50 and EC: 1.5.1.3) more abundant in the three myrmecophagous species than in mammals with other diets (Fig. 3). Therefore, synthesis of folic acid by the microbiome may facilitate formic acid metabolism by the host and may be selectively advantageous during the evolution of host myrmecophagy. These results suggest that synergistic action of host systems and gut microbes may provide myrmecophagous mammals with generally high tolerance of xenobiotic substances.

This study has several limitations that need to be considered. First, the sizes of samples used in the transcriptome and metagenomic analyses were limited, mainly because it is very difficult to obtain more samples of these wild animals due to their inaccessible habitats and wildlife protection policies. To minimize effects of small sample sizes, we ensured that there were at least three repetitions of each sample of these myrmecophagous mammals. Second, the fecal samples used in the metagenomic analyses were from zoos, where the diet may differ from that of the corresponding animals in native habitats. In efforts to minimize the effects of such differences on the results, the myrmecophagous mammals were fed with dried ants as part of their routine diet before we collected the samples. In future studies these limitations could potentially be avoided by collecting more samples, especially from the wild species in native habitats.

## CONCLUSIONS

This study provides the first hologenomic insights into myrmecophagous adaptations. There are many examples of the contributions of symbiotic microbes to the dietary needs of their hosts. However, the interplay between the hosts and their microbiota has been largely unexplored. Using myrmecophagy in multiple mammalian orders as a model, our hologenomic study illuminates the complex and diverse interactions between hosts and their symbiotic gut microbiota. Our study contributes to the understanding of the dietary evolution of mammals and the mechanisms involved in the coordination of physiological functions by animal hosts and their gut commensals.

## METHODS

### Ethical approval

Necessary research permits and ethical approvals were granted (Supplementary Data).

### *De novo* genome sequencing and assembly

Genomic DNA from samples of a lesser anteater (*T. tetradactyla*) and a short-beaked echidna (*T. aculeatus*) was extracted. 20 kb insert PacBio sequencing libraries were constructed and SMRT cells of data were generated. PacBio reads were corrected and assembled into a draft assembly using Wtdbg. A two-step polishing strategy was applied (Supplementary Data).

### Repeat identification, gene prediction and annotation

The lesser anteater and short-beaked echidna genomes were searched for repeats using RepeatMasker [68], the Repbase library [69] and TRF [70]. We predicted genes based on *ab initio* prediction and homology-based gene prediction approaches. Gene functions were assigned in Swissprot [71], Trembl [71], KEGG [72] and GO [73] databases (Supplementary Data).

### Gene family analysis and phylogenetic tree construction

Gene families were constructed using TreeFam [74] and CAFE [75] based on 13 species, including 3 myrmecophagous species (lesser anteater, short-beaked echidna and Malayan pangolin [76]) and 10 non-myrmecophagous species. A phylogenetic tree was reconstructed with the maximum-likelihood algorithm, as implemented in RaxML [77] (Supplementary Data).

### Positive selection test

The single-copy orthologous genes were identified by InParanoid [78]. We used the branch-site model analysis in the Codeml of the PAML package [79] to detect PSGs in each of the three myrmecophagous mammal genomes (Supplementary Data).

### Convergent amino acid substitutions

Ancestral protein sequences were reconstructed using the Codeml program in PAML [79]. The extant sequences were compared to the ancestral sequences, and the observed numbers of convergent/parallel amino acid substitutions were calculated. A Poisson test was used to verify whether the observed number of convergent/parallel sites of each gene was significantly greater than the expected number [80] (Supplementary Data).

### Transcriptome sequencing and assembly

Total RNA was extracted from tissue samples of Malayan pangolins and dogs, and sequenced using Illumina sequencing platforms. We used HISAT-StringTie [81] for transcriptome assembly and quantified the expression levels in terms of fragments per kilo base (kb) of exon per million fragments mapped (FPKM) [82] (Supplementary Data).

### Analyses of correlations between tissue transcriptomes

Spearman's correlation coefficients [83] were calculated and the ‘outliers’ were removed (Supplementary Data).

### Analysis of differential gene expression

Differentially expressed genes were identified using two methods implemented in DESeq2 [84] and edgeR [85] (Supplementary Data).

### Quantitative reverse transcription PCR

Expression levels of four DHEGs (*CHIA, AMDHD2, GNPDA1* and *TREH*) that were potentially involved in diet-related chitin and trehalose metabolism were verified by RT-qPCR (Supplementary Data).

### Metagenomic sequencing and assembly

DNA from the fecal samples of Malayan pangolins and giant anteaters were extracted (Supplementary Table S9) and sequenced with an Illumina HiSeq2000 platform. Metagenomic data of five omnivorous dogs were downloaded from a previous study [86] (Supplementary Data).

Reads from each individual and each species were assembled using MEGAHIT [87]. Contigs were used to predict ORFs using MetaGeneMark [88]. CD-Hit [89] was used to obtain a non-redundant gene catalogue (Supplementary Data).

### Metagenomic taxonomic annotation and functional analysis

The non-redundant gene catalogues were searched against the NCBI non-redundant protein database. MetaGenome Analyzer (MEGAN) software [90] was used to obtain taxonomic annotation. For functional analyses, non-redundant genes were blasted against the KEGG database for annotation. The eggNOG mapper [91] was used for GO annotations. In addition, carbohydrate-active enzymes (CAZymes) annotations were obtained using dbCAN2 [92] (Supplementary Data).

The CPCoA [93] was applied to investigate patterns of separation between the samples from the pangolin, anteater and dog.

### Comparative analyses of the short-beaked echidna and other mammals

A comparative analysis of previously published metagenomic data on fecal samples of the myrmecophagous short-beaked echidna [51] and 38 other mammals (Supplementary Table S10) was performed. Clean reads were annotated by alignment with entries in the NR, GO, KEGG and CAZy databases (Supplementary Data).

The CPCoA [93] was used to investigate patterns of separation between the samples from the short-beaked echidna and mammals with other diets (Supplementary Data).

### Chitinolytic and trehalase activity assays

Digestive tissues from pangolins and one dog were used for the chitinolytic and trehalase assays, using a Chitinase Assay Kit and a Trehalase Assay Kit, respectively. We measured the pH in different parts of the gastrointestinal (GI) tract of the pangolin using a Microprocessor pH meter (Supplementary Data).

## DATA AVAILABILITY

The *de novo* genome assemblies have been deposited in the Genome Sequence Archive (GSA) (http://gsa.big.ac.cn/index.jsp) under accession numbers WGS020015 and WGS020016. Raw RNA-seq data are available in the GSA under accession number CRA004191. Raw metagenomic sequencing data are available in the GSA under accession number CRA004231. All data are available in the main text or the Supplementary Data.

## Supplementary Material

nwac174_Supplemental_File
