# Supplementary material for: Hologenomic insights into mammalian adaptations to myrmecophagy
Source: Natl Sci Rev. 2022 Aug 24;10(4):nwac174. doi: 10.1093/nsr/nwac174 (PMC10139702; doi:10.1093/nsr/nwac174)
Supplement: nwac174_Supplemental_File [file nwac174_supplemental_file.docx]

**Supplementary Information for Hologenomic Insights into Mammalian Adaptations to Myrmecophagy**

Shao-Chen Cheng^1,¶^, Chun-Bing Liu^1,¶^, Xue-Qin Yao^1,¶^, Jing-Yang Hu^1,¶^, Ting-Ting Yin^2^, Burton K Lim^3^, Wu Chen^4^, Guo-Dong Wang^2,5^, Cheng-Lin Zhang^6^, David M Irwin^7^, Zhi-Gang Zhang^1,*^ Ya-Ping Zhang^1,2,5*^, Li Yu^1,*^

^1^State Key Laboratory for Conservation and Utilization of Bio-Resources in Yunnan, School of Life Sciences, Yunnan University, Kunming, China,

^2^State Key Laboratory of Genetic Resources and Evolution, Kunming Institute of Zoology, Chinese Academy of Sciences, Kunming 650223, China,

^3^Department of Natural History, Royal Ontario Museum, Toronto, ON, Canada,

^4^Guangzhou Zoo, Guangzhou, China,

^5^Center for Excellence in Animal Evolution and Genetics, Kunming Institute of Zoology, Chinese Academy of Sciences, Kunming 650223, China.

^6^Beijing Zoo, Beijing, China,

^7^Department of Laboratory Medicine and Pathobiology, University of Toronto, Toronto, Canada

^¶^ These authors contributed equally to this work.

*Correspondence should be addressed to L.Y. (email: [yuli@ynu.edu.cn](mailto:yuli@ynu.edu.cn)), Y.P.Z. (email: [zhangyp@mail.kiz.ac.cn](mailto:zhangyp@mail.kiz.ac.cn)) or Z.G.Z (email: zhangzhigang@ynu.edu.cn)

**This file contains:**

METHODS

Figures S1 to S4

Tables S1 to S17

REFERENCES

**METHODS**

**Ethical approval**

All necessary research permits and ethical approvals were granted for this study from Yunnan University (Project no. ynucae20190023), Guangzhou Zoo (Project no. GZ_20210318), and the Animal Branch of the Germplasm Bank of Wild Species of the Chinese Academy of Sciences (The Large Research Infrastructure Funding Project no. SYDW-20121220-65).

***De novo* genome sequencing and assembly**

Tissue samples from a lesser anteater (*Tamandua tetradactyla*: order Xenarthra, ROM 108923) and a short-beaked echidna (*Tachygolssus aculeatus*: order Monotremata, ROM 106230) were provided by the Royal Ontario Museum (Toronto, Canada) and used for *de novo* genomic sequencing. Genomic DNA was extracted using the sodium dodecyl sulfate (SDS) DNA extraction method [1]. Libraries with an average insert size of 20 Kb were constructed using SMRTbell Template Prep Kits and sequenced with PacBio Sequel instruments at the Genome Center of NextOmics Bioscience Co., Ltd (Wuhan, China). In total, 36 and 54 SMRT cells were run for the lesser anteater and short-beaked echidna genomes, respectively (Supplementary Table S1).

After filtering out adaptor sequences and low-quality reads, PacBio long reads were corrected using Falcon (v1.8.7) ([https://github.com/Pacfic](https://github.com/%20Pacfic)Biosciences/FALCON) and assembled into a draft assembly using Wtdbg (<https://github>.com/ruanjue/wtdbg). To further improve the accuracy of the genome assemblies, a two-step polishing strategy was applied. We first used Arrow [2] in the SMRT Link (v5.1.0) software package to correct errors in the PacBio long reads, and then used Pilon (v1.20) [3] to correct remaining errors in the sequence contigs with whole-genome shotgun (WGS) data generated with an Illumina Hiseq2000 platform. 186.69 Gb (~60 X) from the lesser anteater (with library insert sizes of 10kb and 12kb) and 53.15 Gb (~20 X) from the short-beaked echidna (with library insert sizes of 12kb and 15kb) were obtained and used for polishing (Supplementary Table S1). CEGMA (v2.5) [4] and BUSCO (v3.0.1) [5] were used to assess the quality and completeness of the genome assemblies. In addition, the Illumina WGS reads were mapped onto the assembled genome using the Burrows-Wheeler Aligner (BWA) (v0.7.12) [6] to calculate the mapping ratios, and total number of homozygous derived alleles (> 10 coverage) were obtained by Genome Analysis Toolkit (GATK) (v4.0) [7] to calculate genome base error rates.

**Repeat identification, gene prediction, and annotation**

The lesser anteater and short-beaked echidna genomes were searched for repeats using RepeatMasker [8], the Repbase library [9] and TRF [10].

Using the repeat-masked genomes, we predicted genes based on *ab initio* prediction and homology-based gene prediction approaches. Augustus [11], Glimmer-HMM [12] and Geneid [13] were used for the *ab initio* predictions. Protein sequences from *Homo sapiens*, *Mus musculus*, *Rattus norvegicus*, *Loxodonta africana*, *Phascolarctos cinereus*, and *Trichechus manatus latirostris* were aligned to the lesser anteater genome and those from *Homo sapiens*, *Mus musculus*, *Rattus norvegicus*, *Phascolarctos cinereus*, *Monodelphis domestica*, and *Ornithorhynchus anatinus* were aligned to the short-beaked echidna genome using Genewise [14] for homology-based gene prediction. Gene sets predicted by the two approaches were integrated using EvidenceModeler [15] to produce the consensus gene models.

Gene functions were assigned according to the best match for each gene in the Swissprot, Treml [16], KEGG [17], and GO [18] databases using Blastall [19] (E value ≤1×10^−5^) and in the InterPro database [20,21] using InterProScan (v5.2-45.0) [20].

**Gene family analysis and phylogenetic tree construction**

Predicted genes in the lesser anteater and short-beaked echidna genomes were used together with those in the Malayan pangolin (*Manis javanica*), dog (*Canis familiaris*), cat (*Felis catus*), human (*Homo sapiens*), cow (*Bos Taurus*), giant panda (*Ailuropoda melanoleuca*), bottlenose dolphin (*Tursiops truncates*), African elephant (*Loxodonta africana*), koala (*Phascolarctos cinereus*), platypus (*Ornithorhynchus anatinus*), and chicken (*Gallus gallus*) genomes, which were downloaded from the NCBI public database (Supplementary Table S2). These genomes were selected to provide the phylogenetic coverage of myrmecophagous and non-myrmecophagous sister taxa, as well as dietary coverage of myrmecophagous, herbivorous, carnivorous, and omnivorous species. Another criterion was high genome quality (scaffold N50 ≥1Mb, and average scaffold N50 of these genomes was 52 Mb). The longest translation form was chosen to represent each gene.

Gene families were constructed using TreeFam [22]. Gene family expansions and contractions were determined using CAFE 4.2 [23]. Based on the 1,686 single-copy genes of the 13 species listed above identified using TreeFam, a phylogenetic tree was reconstructed with the maximum-likelihood algorithm as implemented in RaxML [24]. jModelTest 2 [25] was used to estimate the best-fit nucleotide substitution model in the RaxML analysis. Chicken (*Gallus gallus*) was used as the outgroup. Protein sequences for these single-copy genes were aligned using Prank [26], and protein sequence alignments were transformed back into coding sequence alignments. Divergence times of the analyzed species were estimated using the MCMCtree program implemented in PAML (v4.9) [27]. Four calibration times from the TimeTree database [28] were used, including the divergence times between birds and mammals (312.3-330.4 Ma) [29], between Caniformia and Feliformia of Carnivora (43-65 Mya) [30,31], between Cetacea and Artiodactyla of Cetartiodactyla (53-59 Mya) [31,32], and between Afrotheria and Xenarthra (96-105 Mya) [28]. The resulting phylogenetic tree was used in CAFE 4.2 [23] to infer changes in sizes of gene families using a probabilistic model. Finally, we identified 57, 54 and 83 significantly expanded gene families, with 451, 510 and 382 genes, respectively, in the anteater, echidna, and pangolin genomes.

**Positive selection test**

In total, 4,901 high-confidence single-copy orthologous genes were identified in all 13 of the listed mammals’ genomes (Supplementary Table S2) by InParanoid (v4.1) software [33] with the default settings. We used codeml of the PAML package [27] between neutral and selection model [34] and likelihood ratio tests (LRTs) to detect PSGs in each genome of the three myrmecophagous mammals (lesser anteater, short-beaked echidna and Malayan pangolin). In the analyses, each myrmecophagous species was selected as the foreground branch, and the other two were excluded from the background branches. P values were assigned for each of these 2 LRTs using the chi-squared (χ^2^) distribution with 1 degree of freedom and genes with p value less than 0.05 and corrected for multiple testing by a false discovery rate (FDR) less than 0.05. Finally, we identified 537, 614 and 137 PSGs in the anteater, echidna and pangolin genomes, respectively.

**Convergent amino acid substitutions**

To identify convergent/parallel amino acid substitutions, ancestral protein sequences were first reconstructed for the 4,901 single-copy orthologous genes from the 13 species using the Codeml program in PAML (v4.9) [27]. The extant sequences were then compared to the ancestral sequences. The observed numbers of convergent/parallel amino acid substitutions that occurred in all the three myrmecophagous species (lesser anteater, short-beaked echidna, and Malayan pangolin) and the two myrmecophagous species pairs were calculated. To exclude noise from chance amino acid substitutions, a Poisson test was used to verify whether the observed number of convergent/parallel sites of each gene was significantly greater than the expected number under a random JTT-f_gene_ amino acid substitution model following the method of Zou and Zhang (2015) [35]. P <0.05 represent the number of substitutions not resulting from chance. In addition, to further confirm that the number of substitutions was unlikely to have occurred by chance, convergent/ parallel acid substitutions were also identified in combined branches of the non-myrmecophagous sister species of these three myrmecophagous species as negative controls. The chi-square test was used in these significance tests.

**Transcriptome sequencing and assembly**

There is a general consensus that pangolins and carnivores (dogs and cats) are closely related taxa [29,35-38]. To supplement the genetic information regarding the host genomes, we carried out comparative transcriptome analyses of the myrmecophagous Malayan pangolin (*Manis javanica*) and its closest relative, the omnivorous dog (*Canis familiaris*). The Malayan pangolins, which had died in the process of rescue, were obtained from the Guangzhou Wildlife Rescue Center. Dogs were obtained from the Animal Branch of the Germplasm Bank of Wild Species of Chinese Academy of Sciences. All animal treatments were approved by the ethics committees for animal experiments of these two institutions. We collected samples of 10 tissues, including five associated with food digestion and absorption (stomach, small intestine, colon, liver, and pancreas) and five others (lung, cerebrum, tongue, kidney, and heart) from each of three Malayan pangolins (designated as Mp01T-Mp03T; except for cerebrum tissue of Mp03T, which was not successfully obtained), and three dogs (designated as D01T-D03T) (Supplementary Table S7). We recently published the transcriptomic data for Mp01T (NCBI: PRJNA531293). As already mentioned, significant difference between gastric tissues of dogs and pangolins is that dogs’ oxyntic glands are scattered within the gastric tissue, whereas those of pangolins are concentrated in a highly specialized glandular mass that forms an oval mound elevated above the gastric lumen [39]. Thus, in addition to collecting normal gastric tissue (Mp01T-Mp02T) as in the dogs, we also collected the specialized oxyntic gland tissue of a pangolin (Mp03T). Total RNA was extracted from each tissue using a TRIzol Reagent Kit (Invitrogen) according to the manufacturer’s protocol. Paired-end 100 or 150 bp libraries were sequenced using Illumina HiSeq2000 or 2500 sequencing platforms.

Raw data were cleaned to obtain high-quality reads by removing adaptor sequences and low-quality reads with a cut-off of 25 for average quality scores within a moving window of 5 bp using Btrim (v1.5.3) [40]. We also removed reads less than 60 long (in 100 bp libraries) or 100 bp long (in 150 bp libraries) using Trimmomatic (v0.39) [41]. The quality of reads was checked using FastQC (<http://www.bioinformatics.babraham.ac.uk/projects>/fastqc/). Transcriptomes from each pangolin and dog tissue sample were assembled by mapping the clean reads to the Malayan pangolin (NCBI Project ID: PRJNA529513) [42] and dog (NCBI: CanFam3.1) reference genomes, respectively, to obtain the transcripts using HISAT-StringTie [43]. Expression levels of each transcript in each tissue sample were quantified in terms of fragments per kilo base (kb) of exon per million fragments mapped (FPKM) using a standard formula [44].

**Analyses of correlations between tissue transcriptomes**

Based on the bi-directional BLASTp best-hit method (E value = 1e^-5^), 14,914 one-to- one orthologous genes shared by the pangolin and dog were identified using human protein sequence annotation information as a reference ([ftp://ftp.ncbi.nlm.nih.gov/ genomes/all/GCF/000/](ftp://ftp.ncbi.nlm.nih.gov/%20genomes/all/GCF/000/)001/405/GCF_000001405.39_GRCh38/GCF_000001405.39_GRCh38.p13_protein.faa.gz). To examine correlations between the same tissues of the same species, an expression value (FPKM) matrix of the orthologous genes in the transcripts of each tissue was used to calculate Spearman’s correlation coefficients [45]. A symmetrical heat-map of all tissue samples was generated using the R package pheatmap. Samples with greater inter-group correlation (between different tissues of the same species) than intra-group correlation (between samples of the same tissue of the same species) were regarded as ‘outliers’ and excluded from subsequent analyses. Finally, Spearman’s correlation coefficients and symmetrical heat-maps of the expression values of 14,914 orthologous genes between the two species showed that there was greater inter-group correlation than intra-group correlation for the colon tissue of Mp01T, pancreas of Mp03T, tongue of D02T and stomach of D03T (Supplementary Fig. S3), which were regarded as “outliers” and excluded from the subsequent analyses.

**Analysis of differential gene expression**

For the differential gene analysis, calculated expression values (read counts) were normalized. Differentially expressed genes were identified using two methods implemented in DESeq2 [46] and edgeR [47]. Genes identified by both methods with |log_2_ (fold change) | ≥ 1.5, P < 0.01 and FDR < 0.05 were considered to be differentially expressed [48,49].

To identify differentially highly expressed genes (DHEGs) in tissues related to food digestion and absorption (colon, small intestine, stomach, liver, and pancreas) of the Malayan pangolin, we first identified genes that were significantly more highly expressed in tissues related to food digestion and absorption than in other tissues. We then compared their expression levels to those of orthologous genes in the corresponding food digestion and absorption-related tissues of the dogs. Those showing higher expression levels in the Malayan pangolins than in the dogs were regarded as DHEGs and used for the functional enrichment analyses. GO terms and KEGG pathways with P < 0.05 were regarded as significantly enriched functional annotations.

**Quantitative reverse transcription PCR (RT-qPCR)**

Expression levels of four DHEGs (*CHIA*, *AMDHD2*, *GNPDA1* and *TREH*) identified in the transcriptomes that were potentially involved in diet-related chitin and trehalose metabolism were verified by real-time quantitative reverse transcription PCR (RT-qPCR). The same three Malayan pangolins (Mp01T-Mp03T) and three dogs (D01T-D03T) used for transcriptome analyses were used for the RT-qPCR analysis. Total RNA samples extracted from the five tissues related to food digestion and absorption (stomach, small intestine, colon, liver, and pancreas) and five others (lung, cerebrum, tongue, kidney, and heart) described above for the transcriptome sequencing were reverse-transcribed into cDNA using a RT reagent Kit with gDNA Eraser (Takara, DRR047A) according to the manufacturer’s protocol. Specific primers for the four genes in both species are listed in Supplementary Table S17. Quantitative RT-PCR was performed on a QuantStudio® 5 Real-Time PCR System (Applied Biosystems, Inc.) with the following thermal program: 15 seconds at 95°C, 40 cycles of 5 seconds at 95°C, 30 seconds at 60°C. RT-qPCR experiments were repeated three times per tissue sample. The TATA-Box Binding Protein (TBP) gene was used as a reference gene. *TBP* genes in the pangolin and dog genomes were amplified with primer pair TBP-F: 5′-GTCTGGACTGTTCTTCACTCTT-3′and TBP-R: 5′-GTCTGGACTGTTCTTCACTCTT-3′. Relative expression levels of the genes were determined using the formula 2^−ΔΔC^_T_, where ΔΔC_T_ = (ΔC_T_ _of the gene in the target sample_ – ΔC_T of the gene in the control_).

**Metagenomic sequencing and assembly**

Fecal samples from the three Malayan pangolins (*M. javanica*: Mp01-Mp03) used for the transcriptome sequencing were collected for this analysis. Fecal samples from six giant anteaters (*M. tridactyla*, designated as GA01-GA06), as representatives of anteaters, were collected from Beijing Zoo (Supplementary Table S9). These anteaters were fed with dried ants as part of their routine diet. DNA from the fecal samples was extracted using a QIAamp DNAStool Mini kit (Qiagen) with modifications to the manufacturer’s instructions. The samples were subjected to shotgun metagenomic analysis by preparation of 150 bp paired-end libraries and sequencing with an Illumina HiSeq2000 platform.

In addition, metagenomic data from five omnivorous dogs (designated as D01M-D05M) that were sequenced using an Illumina HiSeq2500 platform in a previous study [50] were downloaded and used in the present analyses.

Raw reads from the pangolins, anteaters, and dogs were cleaned with Trimmomatic [41] by removing sequencing adaptors and low-quality reads. The quality of the reads was checked using FastQC. To remove host reads from the metagenome reads, the filtered reads were mapped against the host genomes, i.e., the available Malayan pangolin genome (NCBI Project ID: PRJNA529513) [42], lesser anteater genome sequenced in this study (NCBI Project ID: PRJNA529513) and the dog reference genome (NCBI: CanFam3.1). For this we used the Burrows-Wheeler Aligner (BWA) (v0.7.17) [6], and only non-mapping reads were retained.

To obtain as comprehensive and abundant a gene catalogue as possible, reads from each individual (individual assembly strategy) and each species (co-assembly strategy) was assembled using MEGAHIT (v1.1.3) [51]. Contigs longer than 500 bp were used for the downstream analyses. Contigs were used to predict open reading frames (ORF) using MetaGeneMark (v3.38) [52] with default parameters. CD-Hit (v4.7) [53] was used to remove redundant sequences with identity parameters exceeding 95% to obtain a non-redundant gene catalogue for each of the three species. The relative abundance of each gene in each sample was estimated by mapping the cleaned reads against the non-redundant gene catalogues then normalizing with respect to the total number of reads for the non-redundant genes by the nucleotide lengths of these genes.

**Metagenomic taxonomic annotation and functional analysis**

The non-redundant gene catalogues were searched against the NCBI non- redundant (NR) protein database (updated 2019_9_2) using DIAMOND (v0.9.22.12) [54] with an E-value cutoff of 1e^−2^. The resulting NR search results were processed to obtain taxonomic annotation using the lowest common ancestor (LCA) algorithm in the MetaGenome Analyzer (MEGAN) software package [55]. The LCA annotation results and gene abundance data were used to obtain the number of genes and abundance information for each sample at each taxonomic classification level.

For functional analyses, non-redundant genes were blasted against the KEGG database (V92) for annotation. We used the Hmmer algorithm [56] in eggNOG mapper [57] to compare them to entries in the eggNOG database [58] for GO annotations. In addition, Carbohydrate-Active Enzymes (CAZy) annotations were obtained using dbCAN2 [59]. The abundance of the KEGG, GO and CAZy entries in each sample was represented by the mean coverage (weighted by their coverage) of the genes belonging to the entry.

Using the database annotation results and relative abundances of genes, constrained principal coordinate analysis (CPCoA) [60] with Bray-Curtis distances was applied to investigate patterns of separation between the samples from the pangolin, anteater and dog. The differences between species were estimated using the ADONIS in the R package vegan [61]. The Wilcoxon rank-sum test (P < 0.05) was used to determine the significance of differences in abundance between these species.

Metagenomic sequencing was also performed on three content samples (colon, small intestine, and stomach) of pangolin (Mp02T), and the processing method was similar to fecal samples (Table S14).

**Comparative analyses of the short-beaked echidna to other mammals**

In addition to the pangolin and anteater, our study included comparative analysis of previously published metagenomic data on fecal samples of the myrmecophagous short-beaked echidna [62], and 38 other mammals (including 11 omnivorous, 21 herbivorous and 6 carnivorous species) (Supplementary Table S10). These metagenomic sequences were generated using the 454 platform. Due to the large difference in sequence depth of the samples in the previously published study [51] (0.003 to 0.052 G, 0.015 G on average; Supplementary Table S10) and this study (2.86 to 0.23G, 6.72 G on average; Supplementary Table S9), the 39 samples sequenced in the previously published study [62] were analyzed separately. Clean reads were annotated by alignment with entries in the NR, GO, KEGG and CAZy databases using the methods described above for the pangolin, anteater, and dog samples. We integrated all annotation entries for each of the 39 samples, and counted the number of times that an entry was detected to identify the best hit for reads from each sample. The counts of annotated reads were then regarded as the abundances of the corresponding genes in the samples.

Using the database annotation results and relative abundances of genes, we applied constrained principal coordinate analysis (CPCoA) [60] with Bray-Curtis distances to investigate patterns of separation between the samples from the short-beaked echidna and mammals with other diets (omnivores, herbivores, and carnivores). The differences between diets were estimated using the ADONIS in the R package vegan [61]. Since only one myrmecophagous short-beaked echidna had been sampled, the Wilcoxon rank-sum test could not be used to assess the significance of differences in abundance between it and mammals of the three other dietary classes. Thus, we only assessed the differences in abundances in the comparative analysis.

**Chitinolytic and trehalase activity assays**

Digestive tissues (stomach, small intestine, and colon tissues) from the three pangolins (Mp01T-Mp03T) and one dog (DA) obtained from the Animal Branch of the Germplasm Bank of Wild Species of Chinese Academy of Sciences were used for the chitinolytic and trehalase assays. Due to the differences in structure of gastric tissues between pangolins and dogs, as described above, gastric tissue from the dog (stomach) was compared to two gastric tissues (stomach and oxidation gland) from the pangolin. In addition, we compared chitinolytic and trehalase activities in gastric, small intestine, and colon contents of three pangolins (except for the gastric content from Mp02T, which was not successfully obtained) and corresponding gut contents of the same dog (Supplementary Table S7).

Chitinolytic and trehalase activities were measured using a Chitinase Assay Kit and a Trehalase Assay Kit (Beijing Solarbio Science & Technology Co., Ltd, China), respectively, according to the manufacturer’s protocols. The 3, 5-dinitrosalicylic acid (DNS) method [63] was used to determine the level of reducing sugars with absorbance detection set at 540 nm. Chitinolytic activity was determined by estimating amounts of N-acetylglucosamine released from a standard curve and expressed in U (μmol N-acetylglucosamine liberated per hour per gram sample). Similarly, trehalase activity was determined using a glucose standard curve and expressed in U (μg glucose liberated per minute per gram sample). Three replicates of each sample were measured.

For the pangolin tissues and gut contents with higher chitinase activities, relative to corresponding dog tissues and contents, the optimal pH for chitinase activities was assessed by measuring activity across a range of pH from 3 to 9. We also measured the pH in different parts of the gastrointestinal (GI) tract of the pangolin using a Microprocessor pH meter (Hanna Instruments®, Italy).

**
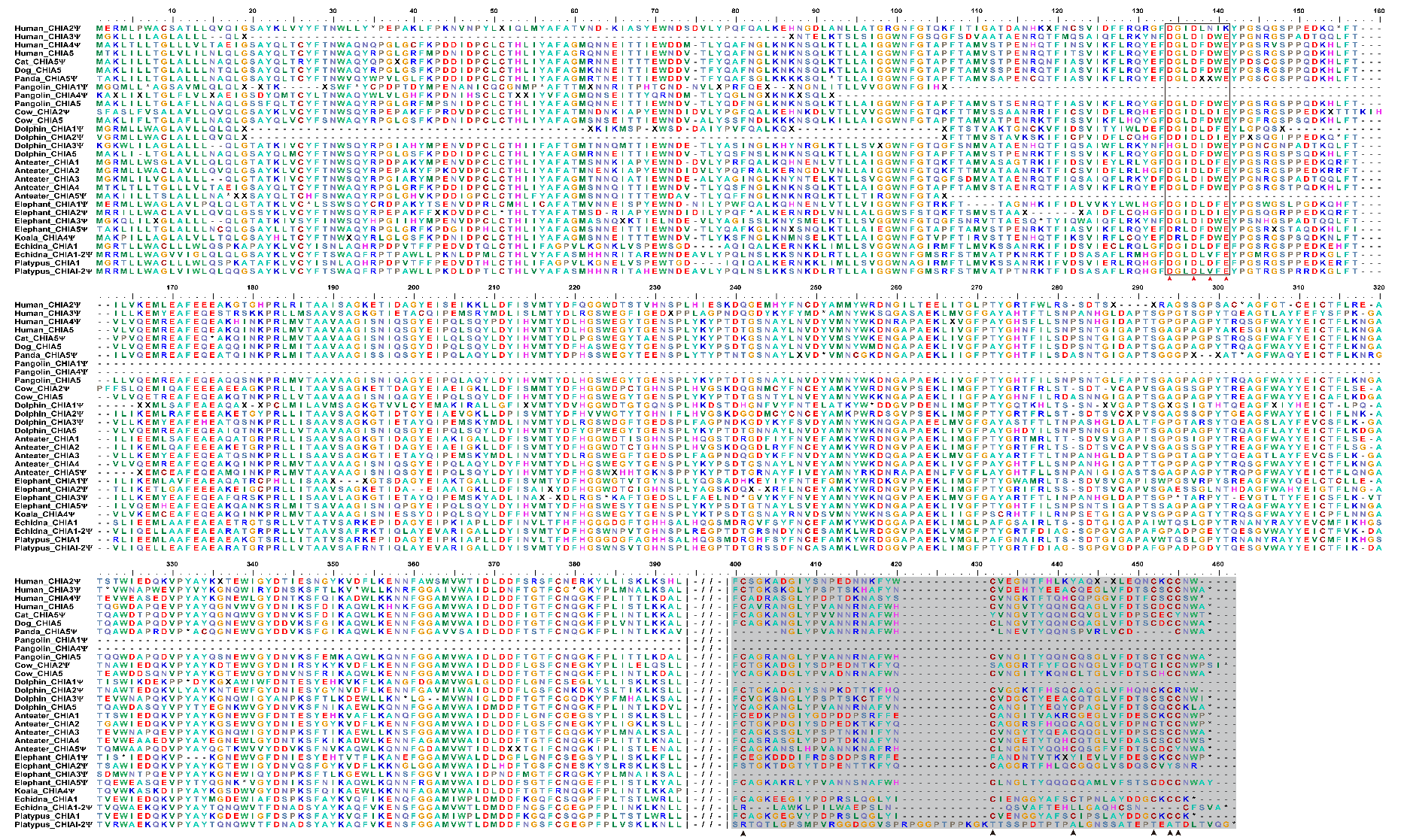
Figure S1: Amino acid sequences of *CHIA* from mammals examined in this study.** Black box indicates a conserved sequence region. Red arrows indicate active site residues. Black arrows indicate cysteine residues. Shadow indicates chitin binding region. “Ψ” indicates pseudogenes.

**
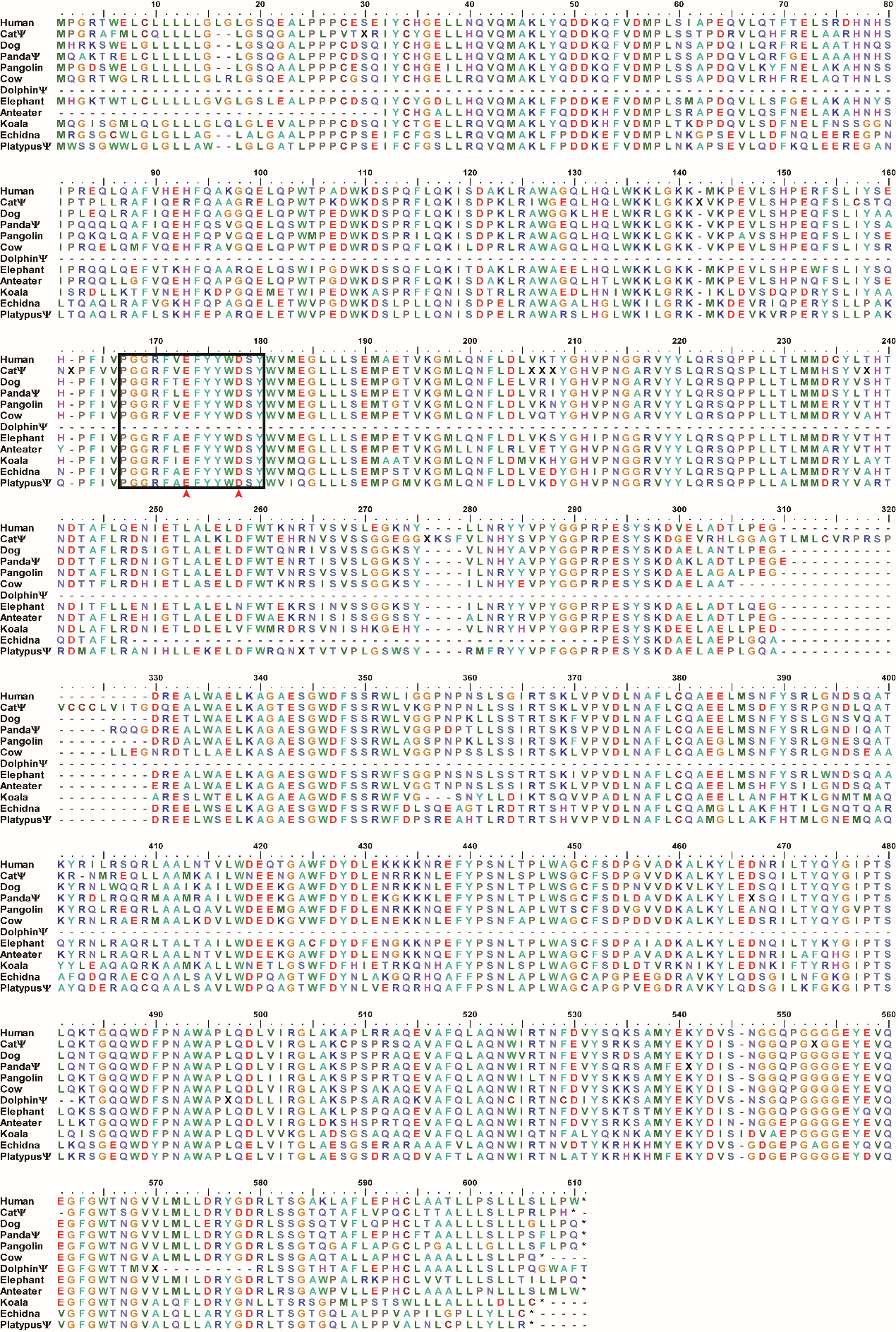
Figure S2: Amino acid sequence of *TREH* from mammals examined in this study.** Black box indicates a conserved sequence region. Red arrows indicate active site residues. “Ψ” indicates pseudogenes.


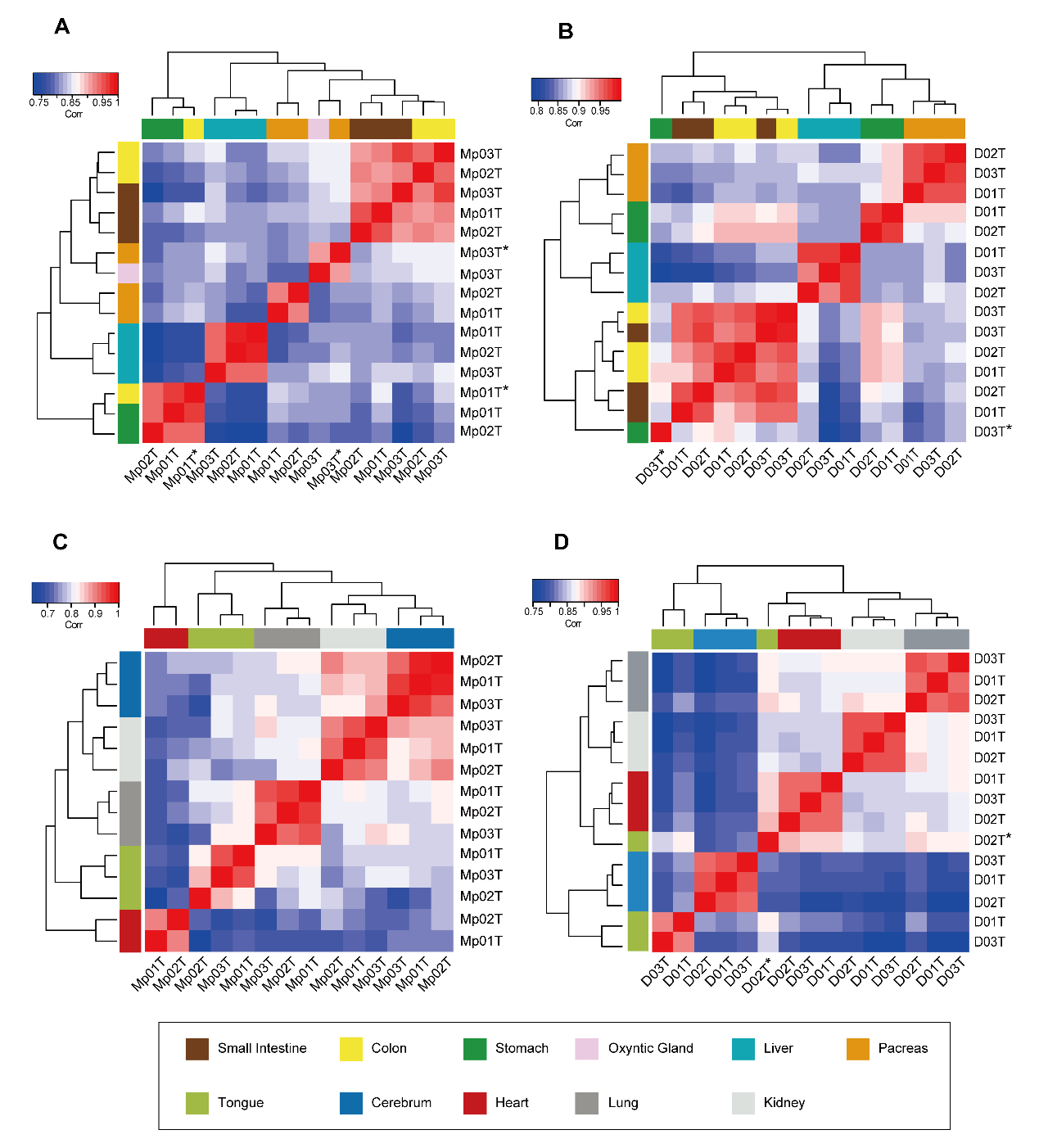
**Figure S3: Symmetrical heat-map of all tissue samples based on Spearman’s correlation coefficients.** (A) and (B) are the clustering results for food digestion and absorption tissues in the pangolin and dog, respectively. (C) and (D) are the clustering results of other tissues in the pangolin and dog, respectively. “*” indicate that the samples have greater inter-group correlation (different tissues of the same species) than intra-group correlation (the same tissue of the same species) and was regarded as an “outlier” and removed from the subsequent analyses.

**
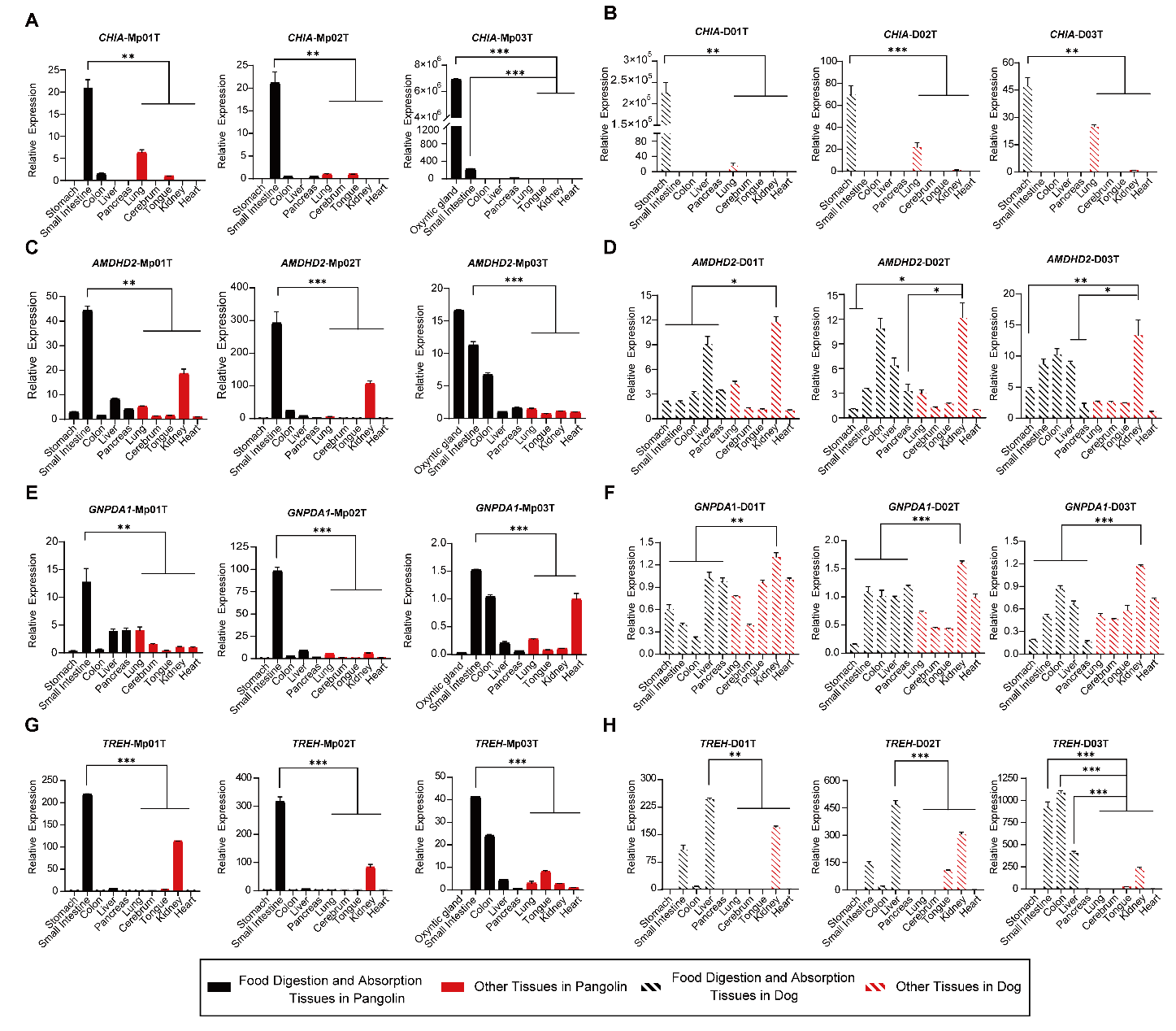
Figure S4:** **Quantitative reverse transcription PCR (RT-qPCR) of four differentially expressed genes from Malayan pangolins compared to dogs.** These four genes were identified using the transcriptome data and verified by qRT-PCR. *CHIA*, *AMDHD2*, and *GNPDA1* (A-F) are involved in diet-related chitin metabolism and *TREH* (G-H) is involved in trehalose metabolism. Mp01T, Mp02T, and Mp03T are the three pangolin individuals examined. D01T, D02T, and D02T are the three dog individuals examined. The five tissues on the left side of the abscissa are related to food digestion and absorption. The last five tissues on the right side of the abscissa are other tissues. Solid bars are from tissues of the pangolin, and bars with skew lines are from tissues from the dog. Mann-Whitney *U* test was used to assess the statistical significance of difference between the two species (* P < 0.05, ** P < 0.01, *** P < 0.001).

**Table S1.** **Genome sequencing and assembly information for anteater and echidna.**

| **Genome Information** | **Anteater** | **Echidna** |
| --- | --- | --- |
| SMRT Cells | 36 | 54 |
| Subreads Data | 188.9 Gb | 212.4 Gb |
| Genome Size | 3.05Gb | 2.37Gb |
| Contig N50 | 3.4 Mb | 8.76 Mb |
| Whole-Genome Shotgun Data Mapping Rate | 98.74% | 98.94% |
| Whole-Genome base error rates | 0.001% | 0.006% |
| CEGMA | 94.35% | 93.95% |
| BUSCO | 87.80% | 91.10% |
| Predicted Genes | 20,027 | 19,982 |
| Annotated Genes | 19,832 | 19,909 |
| Repeat Elements | 43.66% | 54.42% |

**Table S2.** **Information on the genomes from the public NCBI database for species used in the comparative genome analyses.**

| **Common Name** | **Species Name** | **Order** | **Version** | **Access No.** | **Genome Size** |
| --- | --- | --- | --- | --- | --- |
| Human | *Homo sapiens* | Primates | GRCh38.p12 | GCF_000001405 | 3.10 Gb |
| Cat | *Felis catus* | Carnivora | Felis_catus_9.0 | GCF_000181335 | 2.52 Gb |
| Dog | *Canis familiaris* | Carnivora | CanFam3.1 | GCF_000002285 | 2.41 Gb |
| Giant panda | *Ailuropoda melanoleuca* | Carnivora | AilMel_1.0 | GCF_000004335 | 2.29 Gb |
| Malayan pangolin | *Manis javanica* | Pholidota | YNU_ManJav_2.0 | GCF_014570535 | 2.44 Gb |
| Cow | *Bos taurus* | Artiodactyla | ARS-UCD1.2 | GCF_002263795 | 2.72 Gb |
| Dolphin | *Tursiops truncatus* | Artiodactyla | NIST_Tur_tru_v1 | GCF_001922835 | 2.13 Gb |
| Elephant | *Loxodonta africana* | Afrotheria | Loxafr3.0 | GCF_000001905 | 3.20 Gb |
| Koala | *Phascolarctos cinereus* | Marsupialia | phaCin_unsw_v4.1 | GCF_002099425 | 3.20 Gb |
| Platypus | *Ornithorhynchus anatinus* | Monotremata | mOrnAna1.p.v1 | GCF_004115215 | 1.86 Gb |
| Chicken | *Gallus gallus* | Galliformes | GRCg6a | GCF_000002315 | 1.07 Gb |

Table S3. Gene ontology (GO) and Kyoto Encyclopedia of Genes and Genomes (KEGG) enrichment results related to nutrient metabolism derived from the analysis of gene family expansion in three myrmecophagous species. Terms in bold terms overlap between species within a metabolic category. Underlined terms overlap between the present analyses and the transcriptomic analyses of the pangolins (also see Table S8).

| **Metabolism category** | | **Species** | **GOs and KEGGs** |
| --- | --- | --- | --- |
| Nutrient | Carbohydrate | Anteater | GO:0005975 carbohydrate metabolic process, GO:0006006 glucose metabolic process, GO:0032868 response to insulin; ko01200 carbon metabolism, ko04910 insulin signaling pathway, ko04922 glucagon signaling pathway |
|  |  | Pangolin | GO:0042593 glucose homeostasis |
|  |  | Echidna | GO:0009743 response to carbohydrate |
|  | Protein | Anteater | **GO:0015031 protein transport**, GO:0004190 aspartic-type endopeptidase activity; ko00270 cysteine and methionine metabolism |
|  |  | Pangolin | GO:0051603 proteolysis involved in cellular protein catabolic process, GO:1900005 positive regulation of serine-type endopeptidase activity, GO:0004197 cysteine-type endopeptidase activity |
|  |  | Echidna | **GO:0015031 protein transport**; ko00310 lysine degradation |
|  | Lipid | Anteater | GO:0019752 carboxylic acid metabolic process, GO:0016746 transferase activity, transferring acyl groups; ko00640 propanoate metabolism |
|  |  | Pangolin | GO:0019369 arachidonic acid metabolic process, GO:0019372 lipoxygenase pathway, GO:0019395 fatty acid oxidation, GO:0033559 unsaturated fatty acid metabolic process, GO:0034440 lipid oxidation, GO:0043651 linoleic acid metabolic process, GO:0045599 negative regulation of fat cell differentiation, GO:0050873 brown fat cell differentiation, GO:0004052 arachidonate 12(S)-lipoxygenase activity, GO:0016165 linoleate 13S-lipoxygenase activity, GO:0036403 arachidonate 8(S)-lipoxygenase activity, GO:0050473 arachidonate 15-lipoxygenase activity; ko00590 arachidonic acid metabolism |
|  |  | Echidna | GO:0006642 triglyceride mobilization, GO:0010884 positive regulation of lipid storage, GO:0010886 positive regulation of cholesterol storage, GO:0019433 triglyceride catabolic process, GO:0030301 cholesterol transport, GO:0034371 chylomicron remodeling, GO:0034374 low-density lipoprotein particle remodeling, GO:0034378 chylomicron assembly, GO:0034379 very-low-density lipoprotein particle assembly, GO:0034382 chylomicron remnant clearance, GO:0034383 low-density lipoprotein particle clearance, GO:0034447 very-low-density lipoprotein particle clearance, GO:0042158 lipoprotein biosynthetic process, GO:0042159 lipoprotein catabolic process, GO:0042953 lipoprotein transport, GO:0045540 regulation of cholesterol biosynthetic process, GO:0046486 glycerolipid metabolic process, GO:0046834 lipid phosphorylation, GO:0034359 mature chylomicron, GO:0034360 chylomicron remnant, GO:0034361 very-low-density lipoprotein particle, GO:0034362 low-density lipoprotein particle, GO:0034363 intermediate-density lipoprotein particle, GO:0034364 high-density lipoprotein particle, GO:0042627 chylomicron, GO:0035473 lipase binding, GO:0033344 cholesterol efflux, GO:0050750 low-density lipoprotein particle receptor binding, GO:0120020 cholesterol transfer activity; ko00561 glycerolipid metabolism, ko04975 fat digestion and absorption, ko04979 cholesterol metabolism |

**Table S4. GO and KEGG enrichment results related to nutrient metabolism as well as detoxification based on PSGs from myrmecophagous species.** Terms in bold are overlap between species within a metabolic category. Underlined terms are overlap between the present analyses and the transcriptomic analyses of pangolins (also see Table S8).

| **Metabolism category** | | **Species** | **GOs and KEGGs** |
| --- | --- | --- | --- |
| Nutrient | Lipid | Anteater | **GO:0000038 very long-chain fatty acid metabolic process**, GO:0005041 low-density lipoprotein particle receptor activity, **GO:0005782 peroxisomal matrix**, **GO:0006625 protein targeting to peroxisome**, GO:0006629 lipid metabolic process, GO:0006654 phosphatidic acid biosynthetic process, GO:0006707 cholesterol catabolic process, GO:0009395, phospholipid catabolic process, GO:0019216 regulation of lipid metabolic process, GO:0019432 triglyceride biosynthetic process, GO:0030169 low-density lipoprotein particle binding, GO:0034185 apolipoprotein binding, GO:0034201 response to oleic acid, GO:0045444 fat cell differentiation, GO:0050873 brown fat cell differentiation, GO:0060612 adipose tissue development |
|  |  | Pangolin | **GO:0005777 peroxisome**, GO:0006695 cholesterol biosynthetic process, GO:0006699 bile acid biosynthetic process, GO:0008123 cholesterol 7-alpha-monooxygenase activity, GO:0010897 negative regulation of triglyceride catabolic process, GO:0010983 positive regulation of high-density lipoprotein particle clearance, GO:0033489 cholesterol biosynthetic process via desmosterol, GO:0033490 cholesterol biosynthetic process via lathosterol, GO:0050873 brown fat cell differentiation, GO:0070857 regulation of bile acid biosynthetic process, GO:0071397 cellular response to cholesterol, GO:0071401 cellular response to triglyceride, GO:0036111 very long-chain fatty-acyl-CoA metabolic process, GO:0036112 medium-chain fatty-acyl-CoA metabolic process; ko00120 Primary bile acid biosynthesis, ko04979 Cholesterol metabolism |
|  |  | Echidna | **GO:0000038 very long-chain fatty acid metabolic process**, GO:0001561 fatty acid alpha-oxidation, **GO:0005777 peroxisome**, **GO:0005782 peroxisomal matrix**, **GO:0006625 protein targeting to peroxisome**, GO:0045714 regulation of low-density lipoprotein particle receptor biosynthetic process, GO:0070300 phosphatidic acid binding; ko00062 Fatty acid elongation, ko00590 Arachidonic acid metabolism, ko00640 Propanoate metabolism, ko01040 Biosynthesis of unsaturated fatty acids, ko04146 Peroxisome |
|  | Protein | Anteater | GO:0002020 protease binding, GO:0004177 aminopeptidase activity, GO:0044267 cellular protein metabolic process; ko04974 Protein digestion and absorption |
|  |  | Pangolin | GO:0000052 citrulline metabolic process, GO:0006508 proteolysis, GO:0006552 leucine catabolic process, GO:0009072 aromatic amino acid family metabolic process; ko00400 Phenylalanine, tyrosine and tryptophan biosynthesis |
|  |  | Echidna | GO:0070728 leucine binding, GO:0071233 cellular response to leucine, GO:0015031 protein transport |
|  | Carbohydrate | Anteater | **GO:0043569 negative regulation of insulin-like growth factor receptor signaling pathway**, **GO:0008286 insulin receptor signaling pathway**, GO:0009743 response to carbohydrate, GO:0043559 insulin binding, GO:0071333 cellular response to glucose stimulus, GO:0005539 glycosaminoglycan binding |
|  |  | Pangolin | GO:0008506 sucrose:proton symporter activity, GO:0010907 positive regulation of glucose metabolic process, GO:0015770 sucrose transport, GO:0031994 insulin-like growth factor I binding, GO:0050796 regulation of insulin secretion, GO:0051119 sugar transmembrane transporter activity |
|  |  | Echidna | **GO:0043569 negative regulation of insulin-like growth factor receptor signaling pathway**, **GO:0008286 insulin receptor signaling pathway** |
| Detoxification | | Anteater | GO:0042493 response to drug |
|  |  | Echidna | GO:0042910 xenobiotic transmembrane transporter activity |

**Table S5. Evidence of convergent evolution between the host and microbiome level among myrmecophagous mammals.**

|  | **Category** | **Species** | **GOs/KOs/Genes/KO annotations/CAZy families** |
| --- | --- | --- | --- |
| Host | Expanded  genes | Anteater/Echidna | GO:0015031 protein transport |
|  | Positively selected genes | Anteater/Pangolin/Echidna | *MVK* |
|  |  | Echidna/Pangolin | GO:0005777 peroxisome  *HSD17B4* |
|  |  | Anteater/Echidna | GO:0000038 very long-chain fatty acid metabolic process, GO:0005782 peroxisomal matrix, GO:0006625 protein targeting to peroxisome, GO:0008286 insulin receptor signaling pathway  *HACL1, SCARF1, MCCC1, IDE* |
|  |  | Anteater/Pangolin | *LRP2, CYP7A1* |
|  | Convergent / Parallel genes | Anteater/Pangolin &  Anteater/Echidna  &  Echidna/Pangolin | GO:0051223 regulation of protein transport, GO:0019216 regulation of lipid metabolic process, GO:0045834 positive regulation of lipid metabolic process, GO:0008610 lipid biosynthetic process, GO:0046890 regulation of lipid biosynthetic process, GO:0005975 carbohydrate metabolic process |
|  |  | Anteater/Pangolin &  Anteater/Echidna | GO:0006886 intracellular protein transport, GO:1905954 positive regulation of lipid localization, GO:1905952 regulation of lipid localization, GO:0006650 glycerophospholipid metabolic process, GO:0005543 phospholipid binding, GO:0016051 carbohydrate biosynthetic process, GO:0006094 gluconeogenesis, GO:0019319 hexose biosynthetic process, GO:0019318 hexose metabolic process, GO:0046364 monosaccharide biosynthetic process, GO:0005996 monosaccharide metabolic process, GO:0006006 glucose metabolic process, GO:0044270 cellular nitrogen compound catabolic process |
|  |  | Anteater/Pangolin & Echidna/Pangolin | GO:0032787 monocarboxylic acid metabolic process, GO:0006631 fatty acid metabolic process, GO:0031406 carboxylic acid binding, GO:0032868 response to insulin, GO:0009749 response to glucose, GO:0048029 monosaccharide binding, GO:0009746 response to hexose |
| Gut Microbiota | Metagenomic genes | Anteater/Echidna / Pangolin | GO:0003978 UDP-glucose 4-epimerase activity, GO:0004360 glutamine-fructose-6-phosphate transaminase (isomerizing) activity, GO:0004616 phosphogluconate dehydrogenase (decarboxylating) activity, GO:0005351 carbohydrate:proton symporter activity, GO:0008955 peptidoglycan glycosyltransferase activity, GO:0035251 UDP-glucosyltransferase activity, GO:0046527 glucosyltransferase activity, GO:0006522 alanine metabolic process, GO:0006534 cysteine metabolic process, GO:0006554 lysine catabolic process, GO:0006558 L-phenylalanine metabolic process, GO:0006570 tyrosine metabolic process, GO:0006573 valine metabolic process, GO:0009078 pyruvate family amino acid metabolic process, GO:0018249 protein dehydration, GO:0019477 L-lysine catabolic process, GO:0046144 D-alanine family amino acid metabolic process, GO:0046416 D-amino acid metabolic process, GO:0046436 D-alanine metabolic process, GO:0046440 L-lysine metabolic process, GO:1902221 erythrose 4-phosphate/phosphoenolpyruvate family amino acid metabolic process, GO:0006071 glycerol metabolic process, GO:0019216 regulation of lipid metabolic process, GO:0019563 glycerol catabolic process; K13938 folM; dihydromonapterin reductase / dihydrofolate reductase [EC:1.5.1.50 1.5.1.3], ko00362 benzoate degradation, ko00625 chloroalkane and chloroalkene degradation, ko00626 vaphthalene degradation, ko00627 aminobenzoate degradation, ko00643 styrene degradation, ko00930 caprolactam degradation, ko00660 C5-Branched dibasic acid metabolism, ko00072 synthesis and degradation of ketone bodies, ko00561 glycerolipid metabolism, ko00380 tryptophan metabolism, ko0450 selenocompound metabolism, ko00480 glutathione metabolism; GH65 (trehalase), CBM34, GH1, GH23,GH120, PL9 |

**Table S6. GO and KEGG enrichment results related to nutrient metabolism as well as detoxification based on convergent/parallel genes from myrmecophagous species.**

| **Metabolism category** | | **Species** | **GOs and KOs** |
| --- | --- | --- | --- |
| Nutrient | Carbohydrate | Anteater/Pangolin | GO:0009313 oligosaccharide catabolic process, GO:0019319 hexose biosynthetic process, GO:0019318 hexose metabolic process, GO:1901137 carbohydrate derivative biosynthetic process, GO:0046364 monosaccharide biosynthetic process, GO:0016051 carbohydrate biosynthetic process, GO:0010906 regulation of glucose metabolic process, GO:0005996 monosaccharide metabolic process, GO:0006006 glucose metabolic process, GO:0042593 glucose homeostasis, GO:0033500 carbohydrate homeostasis, GO:0005975 carbohydrate metabolic process, GO:0010675 regulation of cellular carbohydrate metabolic process, GO:0033692 cellular polysaccharide biosynthetic process, GO:0000271 polysaccharide biosynthetic process, GO:0010907 positive regulation of glucose metabolic process, GO:0009749 response to glucose, GO:0006109 regulation of carbohydrate metabolic process, GO:0009746 response to hexose, GO:0048029 monosaccharide binding, GO:0050796 regulation of insulin secretion, GO:0008286 insulin receptor signaling pathway, GO:0032869 cellular response to insulin stimulus, GO:0032868 response to insulin |
|  |  | Anteater/Echidna | GO:0006094 gluconeogenesis, GO:0019319 hexose biosynthetic process, GO:0019318 hexose metabolic process, GO:0046364 monosaccharide biosynthetic process, GO:0005996 monosaccharide metabolic process, GO:0006006 glucose metabolic process, GO:0005975 carbohydrate metabolic process, GO:0016051 carbohydrate biosynthetic process, GO:1901136 carbohydrate derivative catabolic process |
|  |  | Echidna/Pangolin | GO:0009743 response to carbohydrate, GO:0005975 carbohydrate metabolic process, GO:0030246 carbohydrate binding, GO:0034284 response to monosaccharide, GO:0009749 response to glucose, GO:0048029 monosaccharide binding, GO:0032868 response to insulin |
|  | Protein | Anteater/Pangolin | GO:0006520 cellular amino acid metabolic process, GO:1901606 alpha-amino acid catabolic process, GO:0009063 cellular amino acid catabolic process, GO:0030163 protein catabolic process, GO:0044257 cellular protein catabolic process, GO:0009072 aromatic amino acid family metabolic process, GO:0051603 proteolysis involved in cellular protein catabolic process, GO:1901605 alpha-amino acid metabolic process, GO:1905369 endopeptidase complex, GO:1905368 peptidase complex, GO:0008233 peptidase activity, GO:0008238 exopeptidase activity, GO:0004175 endopeptidase activity, GO:0004181 metallocarboxypeptidase activity, GO:0004222 metalloendopeptidase activity, GO:0008236 serine-type peptidase activity, GO:0016831 carboxy-lyase activity, GO:0017171 serine hydrolase activity, GO:0008235 metalloexopeptidase activity, GO:0006886 intracellular protein transport, GO:0051223 regulation of protein transport; ko00310 Lysine degradation, ko00250 Alanine, aspartate and glutamate metabolism, ko00260 Glycine, serine and threonine metabolism, ko04974 Protein digestion and absorption |
|  |  | Anteater/Echidna | GO:0051223 regulation of protein transport, GO:0006886 intracellular protein transport, GO:0090316 positive regulation of intracellular protein transport, GO:0051222 positive regulation of protein transport |
|  |  | Echidna/Pangolin | GO:0050708 regulation of protein secretion, GO:0051223 regulation of protein transport |
|  | Lipid | Anteater/Pangolin | GO:0031406 carboxylic acid binding, GO:0046395 carboxylic acid catabolic process, GO:0044242 cellular lipid catabolic process, GO:0071396 cellular response to lipid, GO:0045444 fat cell differentiation, GO:0006635 fatty acid beta-oxidation, GO:0009062 fatty acid catabolic process, GO:1901568 fatty acid derivative metabolic process, GO:0006631 fatty acid metabolic process, GO:0019395 fatty acid oxidation, GO:0015908 fatty acid transport, GO:0045017 glycerolipid biosynthetic process, GO:0046486 glycerolipid metabolic process, GO:0140333 glycerophospholipid flippase activity, GO:0006650 glycerophospholipid metabolic process, GO:0008610 lipid biosynthetic process, GO:0016042 lipid catabolic process, GO:0030258 lipid modification, GO:0034440 lipid oxidation, GO:0034204 lipid translocation, GO:0006869 lipid transport, GO:0015909 long-chain fatty acid transport, GO:0072330 monocarboxylic acid biosynthetic process, GO:0032787 monocarboxylic acid metabolic process, GO:0005543, phospholipid binding, GO:0010875 positive regulation of cholesterol efflux, GO:0032376 positive regulation of cholesterol transport, GO:0045923 positive regulation of fatty acid metabolic process, GO:1905954 positive regulation of lipid localization, GO:0045834 positive regulation of lipid metabolic process, GO:0032370 positive regulation of lipid transport, GO:0032373 positive regulation of sterol transport, GO:0046320 regulation of fatty acid oxidation, GO:0046890 regulation of lipid biosynthetic process, GO:1905952 regulation of lipid localization, GO:0019216 regulation of lipid metabolic process, GO:0032368 regulation of lipid transport, GO:0070542 response to fatty acid, GO:0055094 response to lipoprotein particle; ko00061 Fatty acid biosynthesis, ko01212 Fatty acid metabolism, ko04146 Peroxisome, ko00565 Ether lipid metabolism, ko00071 Fatty acid degradation, ko03320 PPAR signaling pathway |
|  |  | Anteater/Echidna | GO:0046942 carboxylic acid transport, GO:0008203 cholesterol metabolic process, GO:0008289 lipid binding, GO:0008610 lipid biosynthetic process, GO:0055088 lipid homeostasis, GO:0010876 lipid localization, GO:0005319 lipid transporter activity, GO:0006650 glycerophospholipid metabolic process, GO:0005543 phospholipid binding, GO:0008654 phospholipid biosynthetic process, GO:0006644 phospholipid metabolic process, GO:0015914 phospholipid transport, GO:0045834 positive regulation of lipid metabolic process, GO:0046890 regulation of lipid biosynthetic process, GO:0019216 regulation of lipid metabolic process, GO:0090207 regulation of triglyceride metabolic process, GO:0006665 sphingolipid metabolic process, GO:0016125 sterol metabolic process |
|  |  | Echidna/Pangolin | GO:0031406 carboxylic acid binding, GO:0006631 fatty acid metabolic process, GO:0008610 lipid biosynthetic process, GO:0001676 long-chain fatty acid metabolic process, GO:0032787 monocarboxylic acid metabolic process, GO:0046889 positive regulation of lipid biosynthetic process, GO:0045834 positive regulation of lipid metabolic process, GO:0019217 regulation of fatty acid metabolic process, GO:0046890 regulation of lipid biosynthetic process, GO:0019216 regulation of lipid metabolic process; ko04146 Peroxisome |

**Table S7.** **Summary of the transcriptome data sets from the pangolins and dogs.**

| **Species** | **Sample ID** | **Tissues** | **Raw data (Gb)** | **Clean data (Gb)** | **Mapping rate** | **Q30** |
| --- | --- | --- | --- | --- | --- | --- |
| Malayan pangolin (*Manis javanica*) | Mp01T | Colon | 9.79 | 5.2 | 89.63% | 96.62% |
|  |  | Small Intestine | 6.64 | 3.75 | 92.82% | 96.66% |
|  |  | Stomach | 7.97 | 5.85 | 94.49% | 97.10% |
|  |  | Liver | 7.83 | 4.35 | 89.27% | 96.52% |
|  |  | Pancreas | 6.11 | 3.7 | 93.33% | 98.11% |
|  |  | Lung | 7.52 | 3.55 | 85.69% | 96.44% |
|  |  | Cerebrum | 6.76 | 3.85 | 90.33% | 96.62% |
|  |  | Tongue | 6.31 | 4.55 | 92.95% | 93.68% |
|  |  | Kidney | 6.34 | 3.55 | 93.38% | 96.47% |
|  |  | Heart | 6.84 | 3.78 | 90.55% | 96.43% |
|  | Mp02T | Colon | 7.32 | 4.65 | 91.40% | 96.66% |
|  |  | Small Intestine | 7.74 | 5.7 | 89.28% | 96.67% |
|  |  | Stomach | 6.32 | 4.45 | 90.58% | 96.21% |
|  |  | Liver | 6.6 | 4.2 | 92.91% | 96.68% |
|  |  | Pancreas | 6.92 | 4.2 | 92.64% | 96.34% |
|  |  | Lung | 6.12 | 4.45 | 87.44% | 96.34% |
|  |  | Cerebrum | 8 | 5.5 | 83.84% | 95.91% |
|  |  | Tongue | 6.78 | 5.75 | 86.09% | 93.54% |
|  |  | Kidney | 6.72 | 4.75 | 91.96% | 96.21% |
|  |  | Heart | 6.38 | 4.7 | 88.53% | 96.76% |
|  | Mp03T | Colon | 8.3 | 6.95 | 92.52% | 98.47% |
|  |  | Small Intestine | 9.29 | 7.35 | 93.11% | 97.95% |
|  |  | Oxyntic gland | 8.93 | 7.6 | 96.11% | 98.12% |
|  |  | Liver | 11.4 | 9.75 | 89.68% | 98.58% |
|  |  | Pancreas | 10.8 | 9.15 | 91.47% | 97.98% |
|  |  | Lung | 11.2 | 9.45 | 94.33% | 98.51% |
|  |  | Cerebrum | 9.52 | 8 | 92.77% | 98.09% |
|  |  | Tongue | 11.6 | 10 | 90.99% | 98.55% |
|  |  | Kidney | 7.98 | 6.45 | 93.27% | 97.96% |
| Dog (*Canis familiaris*) | D01T | Colon | 6.5 | 4.15 | 95.27% | 95.57% |
|  |  | Small Intestine | 5.4 | 3.05 | 93.01% | 95.10% |
|  |  | Stomach | 7.3 | 5.9 | 96.06% | 97.50% |
|  |  | Liver | 12 | 7.9 | 95.77% | 96.61% |
|  |  | Pancreas | 5.7 | 2.25 | 97.08% | 96.15% |
|  |  | Lung | 10.1 | 8.15 | 96.05% | 97.47% |
|  |  | Cerebrum | 5.2 | 3.9 | 93.57% | 97.37% |
|  |  | Tongue | 8.3 | 4.15 | 94.77% | 94.50% |
|  |  | Kidney | 8.59 | 6.85 | 95.41% | 97.96% |
|  |  | Heart | 9.32 | 7.35 | 95.78% | 97.96% |
|  | D02T | Colon | 7.1 | 5.3 | 94.01% | 97.18% |
|  |  | Small Intestine | 6.63 | 4.3 | 93.93% | 95.93% |
|  |  | Stomach | 6.95 | 3.55 | 93.92% | 94.83% |
|  |  | Liver | 6.07 | 4 | 95.13% | 95.84% |
|  |  | Pancreas | 5.72 | 4 | 97.42% | 95.97% |
|  |  | Lung | 8.28 | 7.35 | 92.44% | 98.17% |
|  |  | Cerebrum | 7.01 | 4.8 | 92.41% | 96.79% |
|  |  | Tongue | 7.54 | 6.65 | 93.74% | 98.02% |
|  |  | Kidney | 7.34 | 6.6 | 93.99% | 98.21% |
|  |  | Heart | 7.38 | 6.65 | 93.41% | 98.05% |
|  | D03T | Colon | 9.5 | 6.6 | 94.02% | 96.55% |
|  |  | Small Intestine | 12 | 7.9 | 91.52% | 96.29% |
|  |  | Stomach | 8.3 | 5.4 | 94.16% | 96.25% |
|  |  | Liver | 6.1 | 4.25 | 93.47% | 96.73% |
|  |  | Pancreas | 6.1 | 2.75 | 97.24% | 94.18% |
|  |  | Lung | 9.49 | 7.85 | 95.44% | 97.60% |
|  |  | Cerebrum | 6.6 | 3.75 | 94.56% | 95.10% |
|  |  | Tongue | 7.1 | 2.95 | 94.27% | 94.13% |
|  |  | Kidney | 9.07 | 7.05 | 96.10% | 97.93% |
|  |  | Heart | 8.86 | 7.1 | 95.11% | 97.95% |

**Table S8. Gene oncology (GO) and Kyoto Encyclopedia of Genes and Genomes (KEGG) enrichment results related to nutrient metabolism and xenobiotic detoxification based on the differentially highly expressed genes (DHEGs) identified in the transcriptomes of the pangolins compared to dogs****.** Underlined terms indicated the overlap between the present analyses and the genomic analyses of pangolins (also see Table S3 and S4).

| **Tissues** | **Metabolism category** | **GOs and KEGGs** |
| --- | --- | --- |
| Colon | Non-chitin carbohydrate | GO:0030246 carbohydrate binding, GO:0044275 cellular carbohydrate catabolic process, GO:0033500 carbohydrate homeostasis, GO:1901143 insulin catabolic process, GO:0035774 positive regulation of insulin secretion involved in cellular response to glucose stimulus; ko00500 starch and sucrose metabolism |
|  | Chitin | GO:0006047 UDP-N-acetylglucosamine metabolic process, |
|  | Protein | GO:0009063 cellular amino acid catabolic process, GO:0009072 aromatic amino acid family metabolic process, GO:2000213 positive regulation of glutamate metabolic process, GO:0004175 endopeptidase activity, GO:0004252 serine-type endopeptidase activity, GO:0006572 tyrosine catabolic process, GO:0006576 cellular biogenic amine metabolic process, GO:0016259 selenocysteine metabolic process, GO:1904464 regulation of matrix metallopeptidase secretion, GO:0034255 regulation of urea metabolic process |
|  | Lipid | GO:0070858 negative regulation of bile acid biosynthetic process, GO:0038181 bile acid receptor activity, GO:0038183 bile acid signaling pathway, GO:1903413 cellular response to bile acid, GO:0038185 intracellular bile acid receptor signaling pathway, GO:0015721 bile acid and bile salt transport, GO:0015913 short-chain fatty acid import, GO:0015552 propionate transmembrane transporter activity, GO:0008028 monocarboxylic acid transmembrane transporter, GO:1902122 chenodeoxycholic acid binding, GO:0042632 cholesterol homeostasis, GO:0050994 regulation of lipid catabolic process, GO:0071401 cellular response to triglyceride, GO:0071398 cellular response to fatty acid, GO:0050482 arachidonic acid secretion, GO:0055088 lipid homeostasis, GO:0034191 apolipoprotein A-I receptor binding, GO:0010884 positive regulation of lipid storage; ko04976 bile secretion |
| Small intestine | Non-chitin carbohydrate | GO:0000272 polysaccharide catabolic process, GO:0044245 polysaccharide digestion, GO:0005975 carbohydrate metabolic process, GO:0044275 cellular carbohydrate catabolic process, GO:0005997 xylulose metabolic process, GO:0005998 xylulose catabolic process, GO:0035774 positive regulation of insulin secretion involved in cellular response to glucose stimulus, GO:1901143 insulin catabolic process; ***ko00520 amino sugar and nucleotide sugar metabolism***, ko00500 starch and sucrose metabolism, ko00040 pentose and glucuronate interconversions |
|  | Chitin | GO:0006030 chitin metabolic process, GO:0006043 glucosamine catabolic process, GO:0003979 UDP-glucose 6-dehydrogenase activity, GO:0004342 glucosamine-6-phosphate deaminase activity, GO:0006046 N-acetylglucosamine catabolic process, GO:0006048 UDP-N-acetylglucosamine biosynthetic process, GO:0008448 N-acetylglucosamine-6-phosphate deacetylase activity |
|  | Protein | GO:0006508 proteolysis, GO:0006570 tyrosine metabolic process, GO:0006593 ornithine catabolic process, GO:1904464 regulation of matrix metallopeptidase secretion, GO:0006577 regulation of matrix metallopeptidase secretion, GO:0006579 amino-acid betaine catabolic process, GO:0008238 exopeptidase activity, GO:0004252 serine-type endopeptidase activity, GO:0004180 carboxypeptidase activity, GO:0004175 endopeptidase activity |
|  | Lipid | GO:0008203 cholesterol metabolic process, GO:0090122 cholesterol ester hydrolysis involved in cholesterol transport, GO:0098856 intestinal lipid absorption, GO:0030299 intestinal cholesterol absorption, GO:0015485 cholesterol binding, GO:0010878 cholesterol storage, GO:0033344 cholesterol efflux, GO:0042632 cholesterol homeostasis, GO:0030301 cholesterol transport, GO:0120020 cholesterol transfer activity, GO:0090209 negative regulation of triglyceride metabolic process, GO:0019433 triglyceride catabolic process, GO:0004806 triglyceride lipase activity, GO:0005504 fatty acid binding, GO:0006631 fatty acid metabolic process, GO:0045717 negative regulation of fatty acid biosynthetic process, GO:0052869 arachidonic acid omega-hydroxylase activity, GO:0010884 positive regulation of lipid storage, GO:0006629 lipid metabolic process, GO:0042953 lipoprotein transport, GO:0042158 lipoprotein biosynthetic process, GO:0034379 very-low-density lipoprotein particle assembly, GO:0034364 high-density lipoprotein particle, GO:0034361 very-low-density lipoprotein particle, GO:0034359 mature chylomicron, GO:0042627 chylomicron, GO:0034360 chylomicron remnant, GO:0000062 fatty-acyl-CoA binding, GO:0004623 phospholipase A2 activity, GO:0008202 steroid metabolic process, GO:0005496 steroid binding, GO:0008395 steroid hydroxylase activity; ko04976 bile secretion, ko04979 cholesterol metabolism, ko04975 fat digestion and absorption, ko00592 alpha-Linolenic acid metabolism, ko00591 linoleic acid metabolism, ko00590 arachidonic acid metabolism |
|  | Xenobiotic detoxification | GO:0009636 response to toxic substance, GO:0017144 drug metabolic process, GO:0042738 exogenous drug catabolic process, GO:1990748 cellular detoxification, GO:1990961 xenobiotic detoxification by transmembrane export across the plasma membrane; ko00980 metabolism of xenobiotics by cytochrome P450, ko00982 drug metabolism - cytochrome P450, ko00983 drug metabolism - other enzymes |
| Stomach (Normal Stomach tissue) | Non-chitin carbohydrate | GO:0071322 cellular response to carbohydrate stimulus |
|  | Protein | GO:0018277 protein deamination, GO:0019538 protein metabolic process, GO:0006577 amino-acid betaine metabolic process, GO:0006579 amino-acid betaine catabolic process, GO:0097274 urea homeostasis |
| Oxyntic gland | Non-chitin carbohydrate | GO:0000272 polysaccharide catabolic process, GO:0044245 polysaccharide digestion, GO:0044275 cellular carbohydrate catabolic process, GO:0046324 regulation of glucose import; |
|  | Chitin | GO:0006030 chitin metabolic process, GO:0006032 chitin catabolic process, GO:0008061 chitin binding, GO:0004568 chitinase activity, GO:0006048 UDP-N-acetylglucosamine biosynthetic process, GO:0043541 UDP-N-acetylglucosamine transferase complex, GO:0003977 UDP-N-acetylglucosamine diphosphorylase activity, GO:0000225 N-acetylglucosaminylphosphatidylinositol deacetylase activity, ko00520 amino sugar and nucleotide sugar metabolism |
|  | Protein | GO:0006555 methionine metabolic process, GO:0006572 tyrosine catabolic process, GO:0006559 L-phenylalanine catabolic process, GO:0019482 beta-alanine metabolic process, GO:1905286 serine-type peptidase complex, GO:0006571 tyrosine biosynthetic process; ko00260 Glycine, serine and threonine metabolism, ko00360 phenylalanine metabolism, ko00400 phenylalanine, tyrosine and tryptophan biosynthesis |
|  | Lipid | GO:0070508 cholesterol import, GO:0032052 bile acid binding, GO:0036315 cellular response to sterol, GO:0006651 diacylglycerol biosynthetic process, GO:0019992 diacylglycerol binding, GO:0008289 lipid binding, GO:0005504 fatty acid binding, GO:0008392 arachidonic acid epoxygenase activity, GO:0032431 activation of phospholipase A2 activity, GO:0003997 acyl-CoA oxidase activity; ko04976 bile secretion, ko04975 fat digestion and absorption, ko00590 arachidonic acid metabolism |
| Liver | Non-chitin carbohydrate | GO:0006041 glucosamine metabolic process, GO:0009311 glucosamine metabolic process, GO:0030212 hyaluronan metabolic process, GO:0006006 glucose metabolic process, GO:0004346 glucose-6-phosphatase activity, GO:0004345 glucose-6-phosphate dehydrogenase activity, GO:0004343 glucosamine 6-phosphate N-acetyltransferase activity, GO:0001951 intestinal D-glucose absorption; ko00500 starch and sucrose metabolism |
|  | Chitin | GO:0004568 chitinase activity |
|  | Protein | GO:0006579 amino-acid betaine catabolic process, GO:0034255 regulation of urea metabolic process, GO:0006533 aspartate catabolic process, GO:0006569 tryptophan catabolic process, GO:0006576 cellular biogenic amine metabolic process, GO:0015801 aromatic amino acid transport, GO:0019442 tryptophan catabolic process to acetyl-CoA, GO:0070779 D-aspartate import across plasma membrane, GO:1905012 regulation of 'de novo' NAD biosynthetic process from tryptophan, GO:2000213 positive regulation of glutamate metabolic process, GO:0008792 arginine decarboxylase activity, GO:0015173 aromatic amino acid transmembrane transporter activity, GO:0030429 kynureninase activity, GO:0042978 ornithine decarboxylase activator activity; ko04974 protein digestion and absorption, ko00250 alanine, aspartate and glutamate metabolism |
|  | Lipid | GO:0008206 bile acid metabolic process, GO:0030573 bile acid catabolic process, GO:0015721 bile acid and bile salt transport, GO:0006699 bile acid biosynthetic process, GO:0038181 bile acid receptor activity, GO:0038183 bile acid signaling pathway, GO:0038185 intracellular bile acid receptor signaling pathway, GO:0002152 bile acid conjugation, GO:1902122 chenodeoxycholic acid binding, GO:0010878 cholesterol storage, GO:0033344 cholesterol efflux, GO:0042632 cholesterol homeostasis, GO:0102294 cholesterol dehydrogenase activity, GO:0032374 regulation of cholesterol transport, GO:0006651 diacylglycerol biosynthetic process, GO:0019432 triglyceride biosynthetic process, GO:0035356 cellular triglyceride homeostasis, GO:0032788 saturated monocarboxylic acid metabolic process, GO:0032789 unsaturated monocarboxylic acid metabolic process, GO:0006636 unsaturated fatty acid biosynthetic process, GO:2001280 positive regulation of unsaturated fatty acid biosynthetic process, GO:0008028 monocarboxylic acid transmembrane transporter activity, GO:0046459 short-chain fatty acid metabolic process, GO:0043648 dicarboxylic acid metabolic process, GO:0043649 dicarboxylic acid catabolic process, GO:0052869 arachidonic acid omega-hydroxylase activity, GO:0052817 very long chain acyl-CoA hydrolase activity, GO:0033540 fatty acid beta-oxidation using acyl-CoA oxidase, GO:1903545 cellular response to butyrate, GO:0071398 cellular response to fatty acid, GO:0052816 long-chain acyl-CoA hydrolase activity, GO:0000038 very long-chain fatty acid metabolic process, GO:0001676 long-chain fatty acid metabolic process, GO:0042759 long-chain fatty acid biosynthetic process, GO:0035338 long-chain fatty-acyl-CoA biosynthetic process, GO:0006633 fatty acid biosynthetic process, GO:0055089 fatty acid homeostasis, GO:0052815 medium-chain acyl-CoA hydrolase activity, GO:0006082 organic acid metabolic process, GO:0008514 organic anion transmembrane transporter activity, GO:0015711 organic anion transport, GO:0034379 very-low-density lipoprotein particle assembly, GO:0034364 high-density lipoprotein particle, GO:0005777 peroxisome, GO:0005782 peroxisomal matrix, GO:0008202 steroid metabolic process, GO:0005496 steroid binding, GO:0008395 steroid hydroxylase activity, GO:0006694 steroid biosynthetic process, GO:0052689 carboxylic ester hydrolase activity, GO:0006637 acyl-CoA metabolic process, GO:0047617 acyl-CoA hydrolase activity; ko00120 primary bile acid biosynthesis, ko04976 bile secretion, ko04979 cholesterol metabolism, ko00062 fatty acid elongation, ko01040 biosynthesis of unsaturated fatty acids, ko04146 peroxisome, ko00561 glycerolipid metabolism, ko00100 steroid biosynthesis |
|  | Xenobiotic detoxification | GO:0006805 xenobiotic metabolic process, GO:0042738 exogenous drug catabolic process; ko00982 drug metabolism - cytochrome P450 |

**Table S9. Summary the metagenomics data sets from pangolins, anteaters, and dogs [64].**

| **Species** | **Sample ID** | **Clean Data (Gb)** | **Clean Data (removing host reads) (Gb)** | **Contigs** | **ORFs** | **Mapping rate (%)** |
| --- | --- | --- | --- | --- | --- | --- |
| Malayan pangolin (*Manis javanica*) | Mp01 | 5.71 | 5.54 | 51,638 | 126,793 | 94.55 |
|  | Mp02 | 5.99 | 4.27 | 55,875 | 163,383 | 91.89 |
|  | Mp03 | 7.77 | 2.86 | 40,108 | 105,357 | 92.41 |
|  | Co-assembly | 19.47 | 12.67 | 136,089 | 356,235 | - |
|  | Total | - | - | 283,710 | 751,768 | - |
| Anteater (*Myrmecophaga tridactyla*) | GA01 | 5.90 | 5.78 | 113,266 | 295,460 | 93.92 |
|  | GA02 | 5.72 | 5.65 | 171,164 | 399,646 | 89.81 |
|  | GA03 | 5.31 | 5.21 | 168,702 | 405,811 | 90.75 |
|  | GA04 | 5.83 | 5.82 | 192,094 | 479,278 | 89.82 |
|  | GA05 | 5.82 | 5.56 | 148,830 | 365,730 | 91.99 |
|  | GA06 | 5.68 | 5.38 | 163,457 | 374,946 | 93.76 |
|  | Co-assembly | 34.26 | 33.4 | 739,032 | 1,868,076 | - |
|  | Total | - | - | 1,696,545 | 4,188,947 | - |
| Dog  (*Canis familiaris*)  (Coelho et al. 2018) | D01M | 10.84 | 8.87 | 81,139 | 229,542 | 93.76 |
|  | D02M | 12.54 | 9.93 | 76,316 | 199,085 | 95.08 |
|  | D03M | 12.09 | 9.45 | 62,796 | 181,732 | 95.53 |
|  | D04M | 12.30 | 10.23 | 108,105 | 297,062 | 92.42 |
|  | D05M | 11.26 | 9.53 | 84,973 | 219,392 | 93.70 |
|  | Co-assembly | 59.03 | 48.01 | 186,813 | 545,719 | - |
|  | Total | - | - | 600,142 | 1,672,532 | - |

**Table S10. Summary the metagenomics data sets from 39 mammals [62].**

| **Diets** | **Sample ID** | **Species** | **Clean Data (Gb)** |
| --- | --- | --- | --- |
| Myrmecophagy | Echidna | Short-beaked echidna | 0.0151 |
| Omnivory | BaboonSTL | Baboon 1 | 0.0117 |
|  | BaboonW | Baboon 2 | 0.0064 |
|  | BlackBr2 | Black bear | 0.0283 |
|  | BlackLemur | Black lemur | 0.0078 |
|  | Callimicos | Callimicos | 0.0112 |
|  | Chimp1 | Chimpanzee 1 | 0.0147 |
|  | Chimp2 | Chimpanzee 2 | 0.0063 |
|  | RTLemur | Ringtailed lemur | 0.0206 |
|  | Saki | Saki | 0.0522 |
|  | SpecBr2 | Spectacled bear | 0.0151 |
|  | Squirrel | Squirrel | 0.0101 |
| Carnivory | BushDog1 | Bush dog | 0.0144 |
|  | Lion1 | Lion 1 | 0.0031 |
|  | Lion2 | Lion 2 | 0.0310 |
|  | Hyena2 | Hyena | 0.0156 |
|  | PolarBr2 | Polar bear | 0.0233 |
|  | Armadillo | Armadillo | 0.0065 |
| Herbivory | AfElphSD3 | African elephant | 0.0239 |
|  | BigHornSD | Bighorn sheep 1 | 0.0182 |
|  | BigHornW3 | Bighorn sheep 2 | 0.0211 |
|  | BlackRhino1 | Black rhinoceros | 0.0192 |
|  | Capybara | Capybara | 0.0236 |
|  | Colobus | Colobus | 0.0147 |
|  | Gazelle3 | Gazelle | 0.0151 |
|  | Giraffe2 | Giraffe | 0.0098 |
|  | GorillaSTL | Gorilla | 0.0038 |
|  | Horse1 | Horse | 0.0211 |
|  | HyraxSD | Rock hyrax 1 | 0.0091 |
|  | HyraxSTL | Rock hyrax 2 | 0.0151 |
|  | Kroo3 | Kangaroo | 0.0049 |
|  | Okapi1 | Okapi 1 | 0.0068 |
|  | Okapi2 | Okapi 2 | 0.0050 |
|  | Orang1 | Orangutan | 0.0059 |
|  | Rabbit | European rabbit | 0.0160 |
|  | SpgbkW | Springbok | 0.0113 |
|  | Urial2 | Transcaspian Urial sheep | 0.0064 |
|  | VWPig | Visayan warty pig | 0.0232 |
|  | ZebraSTL1 | Zebra | 0.0057 |

**Table S11. Bacterial families and genera in the gut microbiomes of pangolins and anteaters are significantly higher abundance than those in the dog.** “†”, “※” and “#” indicated the taxa associated with chitin degradation, secondary bile acid biosynthesis and short-chain fatty acids biosynthesis respectively.

|  | **Taxonomic level** | **Taxa** | **Mean_Pangolins** | **Mean_Dogs** | **P_value** |
| --- | --- | --- | --- | --- | --- |
| Pangolins vs. Dogs | Family | Aeromonadaceae | 0.00681 | 0.00013 | 0.03571 |
|  |  | Enterobacteriaceae | 0.26759 | 0.00151 | 0.03571 |
|  |  | Erysipelotrichaceae | 0.01645 | 0.00429 | 0.03571 |
|  |  | Helicobacteraceae | 0.00064 | 0.00015 | 0.03571 |
|  |  | Lactobacillaceae | 0.01130 | 0.00119 | 0.03571 |
|  |  | Morganellaceae | 0.02586 | 0.00003 | 0.03571 |
|  |  | Oscillospiraceae | 0.00572 | 0.00096 | 0.03571 |
|  |  | Peptostreptococcaceae | 0.00228 | 0.00057 | 0.03571 |
|  |  | Selenomonadaceae | 0.00836 | 0.00464 | 0.03571 |
|  |  | Streptococcaceae | 0.00054 | 0.00004 | 0.03571 |
|  |  | Tannerellaceae | 0.02654 | 0.00097 | 0.03571 |
|  | Genus | *Aeromonas* | 0.00681 | 0.00013 | 0.03571 |
|  |  | *Bilophila* | 0.00267 | 0.00003 | 0.03571 |
|  |  | *Citrobacter*^†^ | 0.00397 | 0 | 0.03571 |
|  |  | *Enterobacter*^†^ | 0.04377 | 0 | 0.03571 |
|  |  | *Escherichia* | 0.06380 | 0.00059 | 0.03571 |
|  |  | *Faecalibaculum* | 0.00006 | 0.00002 | 0.03571 |
|  |  | *Helicobacter* | 0.00063 | 0.00013 | 0.03571 |
|  |  | *Klebsiella*^†^ | 0.07508 | 0.00010 | 0.03571 |
|  |  | *Lactobacillus* | 0.01110 | 0.00119 | 0.03571 |
|  |  | *Lactococcus*^†^ | 0.00051 | 0.00004 | 0.03571 |
|  |  | *Megamonas* | 0.00836 | 0.00458 | 0.03571 |
|  |  | *Morganella* | 0.02586 | 0.00002 | 0.03571 |
|  |  | *Niameybacter* | 0.00145 | 0.00006 | 0.03571 |
|  |  | *Oscillibacter* | 0.00569 | 0.00089 | 0.03571 |
|  |  | *Parabacteroides* | 0.02654 | 0.00096 | 0.03571 |
|  |  | *Peptoclostridium* | 0.00171 | 0.00019 | 0.03571 |
|  |  | *Shigella* | 0.00241 | 0.00001 | 0.03571 |
|  |  | *Streptococcus* | 0.00003 | 0 | 0.03571 |
|  | **Taxonomic level** | **Taxa** | **Mean_Anteaters** | **Mean_Dogs** | **P_value** |
| Anteaters vs. Dogs | Family | Aeromonadaceae | 0.000637 | 0.000133 | 0.00433 |
|  |  | Atopobiaceae | 0.000628 | 1.33E-05 | 0.01732 |
|  |  | Bacillaceae | 0.010359 | 1.67E-05 | 0.00433 |
|  |  | Clostridiaceae | 0.046714 | 0.025059 | 0.00433 |
|  |  | Clostridiales Family XIII. Incertae Sedis | 0.000887 | 5.29E-05 | 0.03030 |
|  |  | Deferribacteraceae | 0.001147 | 6.55E-05 | 0.00433 |
|  |  | Enterobacteriaceae | 0.019066 | 0.001507 | 0.00433 |
|  |  | Erysipelotrichaceae | 0.011285 | 0.004289 | 0.00433 |
|  |  | Eubacteriaceae | 0.005697 | 0.001624 | 0.00433 |
|  |  | Helicobacteraceae | 0.000715 | 0.000151 | 0.00433 |
|  |  | Lachnospiraceae^#^ | 0.030573 | 0.02069 | 0.03030 |
|  |  | Morganellaceae | 0.002556 | 2.74E-05 | 0.00433 |
|  |  | Mycobacteriaceae | 0.00031 | 8.02E-06 | 0.00433 |
|  |  | Odoribacteraceae | 0.006659 | 0.001412 | 0.00866 |
|  |  | Paenibacillaceae | 0.00167 | 1.89E-05 | 0.00433 |
|  |  | Peptostreptococcaceae | 0.008819 | 0.000571 | 0.00433 |
|  |  | Pseudomonadaceae | 0.000305 | 4.78E-05 | 0.00433 |
|  |  | Ruminococcaceae^#^ | 0.031056 | 0.014792 | 0.01732 |
|  |  | Staphylococcaceae | 0.000707 | 5.71E-05 | 0.00433 |
|  |  | Streptococcaceae | 0.004384 | 4.42E-05 | 0.00433 |
|  |  | Succinivibrionaceae | 0.005444 | 0.001998 | 0.00433 |
|  |  | Sutterellaceae | 0.003655 | 0.000378 | 0.00433 |
|  |  | Synergistaceae | 0.001441 | 0.000126 | 0.00433 |
|  |  | Tannerellaceae | 0.009739 | 0.000973 | 0.00433 |
|  | Genus | *Aeromonas* | 0.00061 | 0.00013 | 0.00433 |
|  |  | *Akkermansia* | 0.00445 | 0.00001 | 0.01732 |
|  |  | *Allobaculum* | 0.00118 | 0.00052 | 0.00433 |
|  |  | *Anaerobiospirillum* | 0.00427 | 0.00161 | 0.00433 |
|  |  | *Anaerotruncus* | 0.00402 | 0.00063 | 0.00866 |
|  |  | *Blautia* | 0.00966 | 0.00043 | 0.00433 |
|  |  | *Butyricimonas* | 0.00216 | 0.00002 | 0.00433 |
|  |  | *Catenibacterium* | 0.00211 | 0.00006 | 0.00433 |
|  |  | *Citrobacter*^†^ | 0.00016 | 0 | 0.00433 |
|  |  | *Cloacibacillus* | 0.00109 | 0.00008 | 0.00433 |
|  |  | *Clostridioides* | 0.00062 | 0.00003 | 0.00433 |
|  |  | *Clostridium*^†※^ | 0.04051 | 0.01637 | 0.00433 |
|  |  | *Collinsella* | 0.00447 | 0.00060 | 0.00433 |
|  |  | *Dialister*^#^ | 0.00053 | 0.00019 | 0.00433 |
|  |  | *Dubosiella* | 0.00066 | 0 | 0.00433 |
|  |  | *Enterobacter*^†^ | 0.00243 | 0 | 0.00433 |
|  |  | *Erysipelatoclostridium* | 0.00071 | 0.00008 | 0.00433 |
|  |  | *Eubacterium*^※^ | 0.00429 | 0.00157 | 0.00866 |
|  |  | *Faecalibaculum* | 0.00098 | 0.00002 | 0.00433 |
|  |  | *Helicobacter* | 0.00069 | 0.00013 | 0.00433 |
|  |  | *Lachnospira^#^* | 0.00006 | 0 | 0.00433 |
|  |  | *Megasphaera* | 0.00128 | 0.00006 | 0.00866 |
|  |  | *Morganella* | 0.00240 | 0.00002 | 0.00433 |
|  |  | *Mucispirillum* | 0.00114 | 0.00006 | 0.00433 |
|  |  | *Niameybacter* | 0.00105 | 0.00006 | 0.00433 |
|  |  | *Paenibacillus* | 0.00151 | 0.00001 | 0.00433 |
|  |  | *Parabacteroides* | 0.00951 | 0.00096 | 0.00866 |
|  |  | *Pseudomonas* | 0.00030 | 0.00005 | 0.00433 |
|  |  | *Robinsoniella* | 0.00051 | 0.00002 | 0.00433 |
|  |  | *Ruminococcus* | 0.00470 | 0.00129 | 0.00433 |
|  |  | *Staphylococcus*^†^ | 0.00070 | 0.00006 | 0.00433 |
|  |  | *Streptococcus* | 0.00360 | 0 | 0.00433 |
|  |  | *Subdoligranulum* | 0.00145 | 0.00009 | 0.00433 |
|  |  | *Sutterella* | 0.00322 | 0.00012 | 0.00433 |
|  |  | *Terrisporobacter* | 0.00064 | 0 | 0.00433 |
|  |  | *Tyzzerella* | 0.00050 | 0.00014 | 0.01732 |

**Table S12. Bacterial families and bacterial genera in the echidna are higher abundance than in 38 other mammals with different dietary types [62].** “†” , “※” and “#” indicated the taxa associated with chitin degradation, secondary bile acid biosynthesis and short-chain fatty acids biosynthesis respectively.

| **Taxonomic level** | **Taxa** | **Echidna** | **Mean_ Carnivority** | **Mean_ Herbivory** | **Mean_ Omnivory** |
| --- | --- | --- | --- | --- | --- |
| Family | Cellulomonadaceae | 0.00792 | 0 | 0.00020 | 0.00092 |
|  | Desulfovibrionaceae | 0.00764 | 0.00587 | 0.00730 | 0.00742 |
|  | Clostridiales Family XIII. Incertae Sedis | 0.00959 | 0.00073 | 0.00209 | 0.00301 |
|  | Flavobacteriaceae | 0.01376 | 0.00208 | 0.00286 | 0.00237 |
|  | Synergistaceae | 0.02056 | 0 | 0.00088 | 0.00055 |
|  | Eubacteriaceae | 0.02376 | 0.01760 | 0.01539 | 0.01542 |
|  | Tannerellaceae | 0.01640 | 0.00429 | 0.00750 | 0.00537 |
|  | Fibrobacteraceae | 0.00945 | 0.00562 | 0.00570 | 0.00829 |
|  | Peptococcaceae | 0.00542 | 0 | 0.00125 | 0.00073 |
|  | Dysgonamonadaceae | 0.00736 | 0 | 0.00010 | 0.00029 |
|  | Lachnospiraceae^#^ | 0.12728 | 0.12334 | 0.10571 | 0.08428 |
|  | Sphingobacteriaceae | 0.00584 | 0 | 0.00062 | 0.00029 |
|  | Paenibacillaceae | 0.01139 | 0.00266 | 0.00454 | 0.00626 |
|  | Opitutaceae | 0.00500 | 0 | 0 | 0.00032 |
|  | Victivallaceae | 0.01459 | 0 | 0.00018 | 0.00057 |
|  | Bacteroidaceae | 0.16854 | 0.06699 | 0.14413 | 0.11928 |
| Genus | *Alistipes* | 0.08667 | 0.03168 | 0.06710 | 0.02716 |
|  | *Anaerotruncus* | 0.00774 | 0.00203 | 0.00409 | 0.00472 |
|  | *Bacteroides*^†^ | 0.24409 | 0.08552 | 0.17516 | 0.14210 |
|  | *Butyrivibrio* | 0.00995 | 0.00309 | 0.00417 | 0.00381 |
|  | *Cellulomonas* | 0.01260 | 0 | 0.00027 | 0.00140 |
|  | *Cloacibacillus* | 0.00995 | 0 | 0.00008 | 0.00014 |
|  | *Eubacterium*^※^ | 0.02344 | 0.01749 | 0.01554 | 0.01437 |
|  | *Fibrobacter* | 0.01503 | 0.00798 | 0.00812 | 0.01138 |
|  | *Lachnoclostridium* | 0.00707 | 0.00234 | 0.00347 | 0.00418 |
|  | *Paenibacillus* | 0.01238 | 0.00235 | 0.00376 | 0.00655 |
|  | *Parabacteroides* | 0.02233 | 0.00462 | 0.00976 | 0.00651 |
|  | *Porphyromonas* | 0.00685 | 0 | 0 | 0 |
|  | *Ruminococcus* | 0.09308 | 0.06435 | 0.06229 | 0.05331 |
|  | *Treponema* | 0.07009 | 0.02589 | 0.02870 | 0.06149 |
|  | *Victivallis* | 0.02321 | 0 | 0.00027 | 0.00080 |

**Table S13.** **Gene oncology (GO) and Kyoto Encyclopedia of Genes and Genomes (KEGG) enrichment results and Carbohydrate-Active Enzymes (CAZy) results related to nutrient metabolism and xenobiotic detoxification based on the higher abundance of classifications in myrmecophagous species compared to species with different diets.** Terms in bold means shared among the three myrmecophagous species.

| **Species** | **Metabolism category** | | **GO and KEGG and CAZy** |
| --- | --- | --- | --- |
| Pangolins vs. Dogs | Nutrient | Non-Chitin- Carbohydrate | KOs+GOs: GO:0000270 peptidoglycan metabolic process, GO:0000272 polysaccharide catabolic process, GO:0001530 lipopolysaccharide binding, GO:0003850 2-deoxyglucose-6-phosphatase activity, **GO:0003978 UDP-glucose 4-epimerase activity**, GO:0004089 carbonate dehydratase activity, GO:0004344 glucose dehydrogenase activity, GO:0004345 glucose-6-phosphate dehydrogenase activity, GO:0004346 glucose-6-phosphatase activity, GO:0004347 glucose-6-phosphate isomerase activity, **GO:0004360 glutamine-fructose-6-phosphate transaminase (isomerizing) activit**y, GO:0004456 phosphogluconate dehydratase activity, GO:0004475 mannose-1-phosphate guanylyltransferase activity, GO:0004476 mannose-6-phosphate isomerase activity, GO:0004555 alpha,alpha-trehalase activity, GO:0004558 alpha-1,4-glucosidase activity, **GO:0004616 phosphogluconate dehydrogenase (decarboxylating) activity**, GO:0004805 trehalose-phosphatase activity, **GO:0005351 carbohydrate:proton symporter activity**, GO:0005353 fructose transmembrane transporter activity, GO:0005354 galactose transmembrane transporter activity, GO:0005402 carbohydrate:cation symporter activity, GO:0005534 galactose binding, GO:0005537 mannose binding, GO:0005539 glycosaminoglycan binding, GO:0005975 carbohydrate metabolic process, GO:0005984 disaccharide metabolic process, GO:0005985 sucrose metabolic process, GO:0005987 sucrose catabolic process, GO:0005991 trehalose metabolic process, GO:0005993 trehalose catabolic process, GO:0005996 monosaccharide metabolic process, GO:0005997 xylulose metabolic process, GO:0005998 xylulose catabolic process, GO:0006000 fructose metabolic process, GO:0006001 fructose catabolic process, GO:0006007 glucose catabolic process, GO:0006012 galactose metabolic process, GO:0006013 mannose metabolic process, GO:0006022 aminoglycan metabolic process, GO:0006026 aminoglycan catabolic process, GO:0006027 glycosaminoglycan catabolic process, GO:0006098 pentose-phosphate shunt, GO:0006109 regulation of carbohydrate metabolic process, GO:0008108 UDP-glucose:hexose-1-phosphate uridylyltransferase activity, GO:0008422 beta-glucosidase activity, GO:0008446 GDP-mannose 4,6-dehydratase activity, GO:0008643 carbohydrate transport, GO:0008653 lipopolysaccharide metabolic process, GO:0008673 2-dehydro-3-deoxygluconokinase activity, GO:0008706 6-phospho-beta-glucosidase activity, GO:0008758 UDP-2,3-diacylglucosamine hydrolase activity, GO:0008788 alpha,alpha-phosphotrehalase activity, GO:0008801 beta-phosphoglucomutase activity, GO:0008873 gluconate 2-dehydrogenase activity, GO:0008874 gluconate 5-dehydrogenase activity, GO:0008875 gluconate dehydrogenase activity, GO:0008876 quinoprotein glucose dehydrogenase activity, GO:0008877 glucose-1-phosphatase activity, GO:0008905 mannose-phosphate guanylyltransferase activity, GO:0008918 lipopolysaccharide 3-alpha-galactosyltransferase activity, GO:0008919 lipopolysaccharide glucosyltransferase I activity, GO:0008920 lipopolysaccharide heptosyltransferase activity, GO:0008928 mannose-1-phosphate guanylyltransferase (GDP) activity, **GO:0008955 peptidoglycan glycosyltransferase activity**, GO:0008960 phosphatidylglycerol-membrane-oligosaccharide glycerophosphotransferase activity, GO:0009052 pentose-phosphate shunt, non-oxidative branch, GO:0009253 peptidoglycan catabolic process, GO:0009254 peptidoglycan turnover, GO:0009255 Entner-Doudoroff pathway through 6-phosphogluconate, GO:0009274 peptidoglycan-based cell wall, GO:0009311 oligosaccharide metabolic process, GO:0009313 oligosaccharide catabolic process, GO:0010675 regulation of cellular carbohydrate metabolic process, GO:0010676 positive regulation of cellular carbohydrate metabolic process, GO:0015128 gluconate transmembrane transporter activity, GO:0015144 carbohydrate transmembrane transporter activity, GO:0015145 monosaccharide transmembrane transporter activity, GO:0015146 pentose transmembrane transporter activity, GO:0015154 disaccharide transmembrane transporter activity, GO:0015157 oligosaccharide transmembrane transporter activity, GO:0015159 polysaccharide transmembrane transporter activity, GO:0015407 ATPase-coupled monosaccharide transmembrane transporter activity, GO:0015422 ATPase-coupled oligosaccharide transmembrane transporter activity, GO:0015478 oligosaccharide transporting porin activity, GO:0015517 galactose:proton symporter activity, GO:0015578 mannose transmembrane transporter activity, GO:0015608 carbohydrate-importing ATPase activity, GO:0015640 peptidoglycan peptide transmembrane transporter activity, GO:0015649 2-keto-3-deoxygluconate:proton symporter activity, GO:0015670 carbon dioxide transport, GO:0015749 monosaccharide transmembrane transport, GO:0015750 pentose transmembrane transport, GO:0015755 fructose transmembrane transport, GO:0015757 galactose transmembrane transport, GO:0015760 glucose-6-phosphate transport, GO:0015761 mannose transmembrane transport, GO:0015766 disaccharide transport, GO:0015770 sucrose transport, GO:0015771 trehalose transport, GO:0015772 oligosaccharide transport, GO:0015774 polysaccharide transport, GO:0015834 peptidoglycan-associated peptide transport, GO:0015926 glucosidase activity, GO:0015927 trehalase activity, GO:0015976 carbon utilization, GO:0016052 carbohydrate catabolic process, GO:0016137 glycoside metabolic process, GO:0016139 glycoside catabolic process, GO:0016741 transferase activity, transferring one-carbon groups, GO:0016857 racemase and epimerase activity, acting on carbohydrates and derivatives, GO:0017057 6-phosphogluconolactonase activity, GO:0019200 carbohydrate kinase activity, GO:0019203 carbohydrate phosphatase activity, GO:0019309 mannose catabolic process, GO:0019321 pentose metabolic process, GO:0019323 pentose catabolic process, GO:0019521 D-gluconate metabolic process, GO:0019595 non-phosphorylated glucose catabolic process, GO:0019655 glycolytic fermentation to ethanol, GO:0019659 glucose catabolic process to lactate, GO:0019660 glycolytic fermentation, GO:0019662 non-glycolytic fermentation, GO:0019673 GDP-mannose metabolic process, GO:0019755 one-carbon compound transport, GO:0022870 protein-N(PI)-phosphohistidine-mannose phosphotransferase system transporter activity, GO:0030203 glycosaminoglycan metabolic process, GO:0032881 regulation of polysaccharide metabolic process, GO:0033037 polysaccharide localization, GO:0034219 carbohydrate transmembrane transport, **GO:0035251 UDP-glucosyltransferase activity**, GO:0035429 gluconate transmembrane transport, GO:0042132 fructose 1,6-bisphosphate 1-phosphatase activity, GO:0042834 peptidoglycan binding, GO:0043211 ATPase-coupled carbohydrate transmembrane transporter activity, GO:0044247 cellular polysaccharide catabolic process, GO:0044262 cellular carbohydrate metabolic process, GO:0044275 cellular carbohydrate catabolic process, GO:0045913 positive regulation of carbohydrate metabolic process, GO:0045990 carbon catabolite regulation of transcription, GO:0046177 D-gluconate catabolic process, GO:0046316 gluconokinase activity, GO:0046323 glucose import, GO:0046352 disaccharide catabolic process, GO:0046365 monosaccharide catabolic process, GO:0046411 2-keto-3-deoxygluconate transmembrane transport, GO:0046430 non-phosphorylated glucose metabolic process, **GO:0046527 glucosyltransferase activity**, GO:0046835 carbohydrate phosphorylation, GO:0046838 phosphorylated carbohydrate dephosphorylation, GO:0047465 N-acylglucosamine-6-phosphate 2-epimerase activity, GO:0047733 CDP-glucose 4,6-dehydratase activity, GO:0047917 GDP-glucosidase activity, GO:0048029 monosaccharide binding, GO:0048030 disaccharide binding, GO:0050089 mannose isomerase activity, GO:0050580 2,5-didehydrogluconate reductase activity, GO:0050782 galactose uniporter activity, GO:0051691 cellular oligosaccharide metabolic process, GO:0051692 cellular oligosaccharide catabolic process, GO:0052751 GDP-mannose hydrolase activity, GO:0052779 amino disaccharide metabolic process, GO:0052782 amino disaccharide catabolic process, GO:0070413 trehalose metabolism in response to stress, GO:0070415 trehalose metabolism in response to cold stress, GO:0070492 oligosaccharide binding, GO:0097023 fructose 6-phosphate aldolase activity, GO:0097367 carbohydrate derivative binding, GO:1901135 carbohydrate derivative metabolic process, GO:1901136 carbohydrate derivative catabolic process, ko00030 pentose phosphate pathway, ko00051 fructose and mannose metabolism, ko00053 ascorbate and aldarate metabolism, ko00620 pyruvate metabolism |
|  |  |  | CAZy: Glycoside Hydrolases (GH15, GH37, **GH65**, **GH1**, GH102, GH103, GH110, **GH120**, GH123+GH33, GH126, GH13_11, GH13_18, GH13_27, GH13_29, GH13_3, GH13_5, GH144, GH161, **GH23**, GH27, GH37, GH38, GH4, GH43_11, GH43_26, GH43_28, GH43_31, GH5_18, GH76, GH78, GH92+3.2.1.-), GlycosylTransferases (GT0, GT1, GT10, GT101, GT2, GT2_Glyco_tranf_2_3, GT2_Glyco_trans_2_3, GT20, GT51, GT56, GT73, GT94), Polysaccharide Lyases (PL33_1, PL8, PL8_3, **PL9**), Carbohydrate Esterases (CE0, CE9), Auxiliary Activities (AA3_2, AA6, AA7), Carbohydrate-Binding Modules (CBM20+CBM34+GH13_39, CBM32, **CBM34**, CBM35, CBM48, CBM66) |
|  |  | Chitin | KOs+GOs: GO:0004563 beta-N-acetylhexosaminidase activity, GO:0004568 chitinase activity, GO:0006030 chitin metabolic process, GO:0006032 chitin catabolic process, GO:0008061 chitin binding, GO:0008448 N-acetylglucosamine-6-phosphate deacetylase activity, GO:0008761 UDP-N-acetylglucosamine 2-epimerase activity, GO:0008780 acyl-[acyl-carrier-protein]-UDP-N-acetylglucosamine O-acyltransferase activity, GO:0008843 endochitinase activity, GO:0015764 N-acetylglucosamine transport, GO:0015929 hexosaminidase activity, GO:0036311 chitin disaccharide deacetylase activity, GO:0045127 N-acetylglucosamine kinase activity, GO:0052777 diacetylchitobiose catabolic process, GO:0052778 diacetylchitobiose metabolic process |
|  |  |  | CAZy: chitinase (GH18, CBM5+GH18, CBM5+GH19), chitin deacetylase (CE4+GH153), chitosanase (GH8), lytic chitin monooxygenase (AA10), chitin-binding modules (CBM50), β-hexosaminidase (CBM32+GH20) |
|  |  | Protein | GO:0000096 sulfur amino acid metabolic process, GO:0000098 sulfur amino acid catabolic process, GO:0000209 protein polyubiquitination, GO:0001932 regulation of protein phosphorylation, GO:0001934 positive regulation of protein phosphorylation, GO:0003756 protein disulfide isomerase activity, GO:0003958 NADPH-hemoprotein reductase activity, GO:0004174 electron-transferring-flavoprotein dehydrogenase activity, GO:0004177 aminopeptidase activity, GO:0004180 carboxypeptidase activity, GO:0004181 metallocarboxypeptidase activity, GO:0004314 [acyl-carrier-protein] S-malonyltransferase activity, GO:0004721 phosphoprotein phosphatase activity, GO:0004722 protein serine/threonine phosphatase activity, GO:0004725 protein tyrosine phosphatase activity, GO:0004842 ubiquitin-protein transferase activity, GO:0004866 endopeptidase inhibitor activity, GO:0006468 protein phosphorylation, GO:0006470 protein dephosphorylation, GO:0006520 cellular amino acid metabolic process, **GO:0006522 alanine metabolic process**, GO:0006525 arginine metabolic process, GO:0006527 arginine catabolic process, **GO:0006534 cysteine metabolic process**, GO:0006541 glutamine metabolic process, GO:0006543 glutamine catabolic process, GO:0006544 glycine metabolic process, GO:0006546 glycine catabolic process, GO:0006549 isoleucine metabolic process, GO:0006551 leucine metabolic process, GO:0006553 lysine metabolic process, **GO:0006554 lysine catabolic process**, GO:0006555 methionine metabolic process, **GO:0006558 L-phenylalanine metabolic process**, GO:0006562 proline catabolic process, GO:0006563 L-serine metabolic process, GO:0006565 L-serine catabolic process, GO:0006566 threonine metabolic process, GO:0006567 threonine catabolic process, GO:0006569 tryptophan catabolic process, **GO:0006570 tyrosine metabolic process**, **GO:0006573 valine metabolic process**, GO:0006575 cellular modified amino acid metabolic process, GO:0006593 ornithine catabolic process, GO:0006749 glutathione metabolic process, GO:0008113 peptide-methionine (S)-S-oxide reductase activity, GO:0008138 protein tyrosine/serine/threonine phosphatase activity, GO:0008213 protein alkylation, GO:0008233 peptidase activity, GO:0008234 cysteine-type peptidase activity, GO:0008236 serine-type peptidase activity, GO:0008237 metallopeptidase activity, GO:0008238 exopeptidase activity, GO:0008242 omega peptidase activity, GO:0008320 protein transmembrane transporter activity, GO:0008693 3-hydroxydecanoyl-[acyl-carrier-protein] dehydratase activity, GO:0008770 [acyl-carrier-protein] phosphodiesterase activity, GO:0008773 [protein-PII] uridylyltransferase activity, GO:0008779 acyl-[acyl-carrier-protein]-phospholipid O-acyltransferase activity, GO:0008780 acyl-[acyl-carrier-protein]-UDP-N-acetylglucosamine O-acyltransferase activity, GO:0008798 beta-aspartyl-peptidase activity, GO:0008876 quinoprotein glucose dehydrogenase activity, GO:0008965 phosphoenolpyruvate-protein phosphotransferase activity, GO:0008982 protein-N(PI)-phosphohistidine-sugar phosphotransferase activity, GO:0008999 ribosomal-protein-alanine N-acetyltransferase activity, GO:0009046 zinc D-Ala-D-Ala carboxypeptidase activity, GO:0009063 cellular amino acid catabolic process, GO:0009064 glutamine family amino acid metabolic process, GO:0009065 glutamine family amino acid catabolic process, GO:0009066 aspartate family amino acid metabolic process, GO:0009069 serine family amino acid metabolic process, GO:0009071 serine family amino acid catabolic process, GO:0009072 aromatic amino acid family metabolic process, GO:0009074 aromatic amino acid family catabolic process, **GO:0009078 pyruvate family amino acid metabolic process**, GO:0009081 branched-chain amino acid metabolic process, GO:0009087 methionine catabolic process, GO:0009092 homoserine metabolic process, GO:0009100 glycoprotein metabolic process, GO:0009249 protein lipoylation, GO:0009977 proton motive force dependent protein transmembrane transporter activity, GO:0010133 proline catabolic process to glutamate, GO:0015031 protein transport, GO:0015450 P-P-bond-hydrolysis-driven protein transmembrane transporter activity, GO:0016540 protein autoprocessing, GO:0016567 protein ubiquitination, GO:0016579 protein deubiquitination, GO:0016807 cysteine-type carboxypeptidase activity, GO:0017003 protein-heme linkage, GO:0017006 protein-tetrapyrrole linkage, GO:0017038 protein import, GO:0018065 protein-cofactor linkage, GO:0018126 protein hydroxylation, GO:0018175 protein nucleotidylation, GO:0018215 protein phosphopantetheinylation, **GO:0018249 protein dehydration**, GO:0018298 protein-chromophore linkage, GO:0019171 3-hydroxyacyl-[acyl-carrier-protein] dehydratase activity, GO:0019243 methylglyoxal catabolic process to D-lactate via S-lactoyl-glutathione, **GO:0019477 L-lysine catabolic process**, GO:0019478 D-amino acid catabolic process, GO:0019529 taurine catabolic process, GO:0019530 taurine metabolic process, GO:0019545 arginine catabolic process to succinate, GO:0019665 anaerobic amino acid catabolic process, GO:0019787 ubiquitin-like protein transferase activity, GO:0022870 protein-N(PI)-phosphohistidine-mannose phosphotransferase system transporter activity, GO:0023014 signal transduction by protein phosphorylation, GO:0030163 protein catabolic process, GO:0030414 peptidase inhibitor activity, GO:0031072 heat shock protein binding, GO:0031249 denatured protein binding, GO:0031455 glycine betaine metabolic process, GO:0031647 regulation of protein stability, GO:0032270 positive regulation of cellular protein metabolic process, GO:0032271 regulation of protein polymerization, GO:0032978 protein insertion into membrane from inner side, GO:0032991 protein-containing complex, GO:0033281 TAT protein transport complex, GO:0033662 modulation by symbiont of host defense-related protein level, GO:0033743 peptide-methionine (R)-S-oxide reductase activity, GO:0035304 regulation of protein dephosphorylation, GO:0035307 positive regulation of protein dephosphorylation, GO:0035601 protein deacylation, GO:0035966 response to topologically incorrect protein, GO:0035967 cellular response to topologically incorrect protein, GO:0036088 D-serine catabolic process, GO:0042176 regulation of protein catabolic process, GO:0042851 L-alanine metabolic process, GO:0043393 regulation of protein binding, GO:0043543 protein acylation, GO:0044257 cellular protein catabolic process, GO:0044314 protein K27-linked ubiquitination, **GO:0046144 D-alanine family amino acid metabolic process**, **GO:0046416 D-amino acid metabolic process**, **GO:0046436 D-alanine metabolic process**, **GO:0046440 L-lysine metabolic process**, GO:0046777 protein autophosphorylation, GO:0046944 protein carbamoylation, GO:0047134 protein-disulfide reductase activity, GO:0050821 protein stabilization, GO:0051223 regulation of protein transport, GO:0051247 positive regulation of protein metabolic process, GO:0051603 proteolysis involved in cellular protein catabolic process, GO:0052547 regulation of peptidase activity, GO:0052548 regulation of endopeptidase activity, GO:0052868 protein-lysine lysyltransferase activity, GO:0061134 peptidase regulator activity, GO:0061135 endopeptidase regulator activity, GO:0070004 cysteine-type exopeptidase activity, GO:0070011 peptidase activity, acting on L-amino acid peptides, GO:0070178 D-serine metabolic process, GO:0070733 protein adenylyltransferase activity, GO:0071806 protein transmembrane transport, GO:0071915 protein-lysine lysylation, GO:0075344 modulation by symbiont of host protein levels, GO:1901605 alpha-amino acid metabolic process, GO:1901606 alpha-amino acid catabolic process, **GO:1902221 erythrose 4-phosphate/phosphoenolpyruvate family amino acid metabolic process,** ko00280 valine, leucine and isoleucine degradation, **ko00310 lysine degradation** ko00350 tyrosine metabolism, **ko00380 tryptophan metabolism**, ko00410 beta-Alanine metabolism, ko00440 phosphonate and phosphinate metabolism, **ko00450 selenocompound metabolism**, **ko00480 glutathione metabolism**, ko04120 ubiquitin mediated proteolysis |
|  |  | Lipid | GO:0001727 lipid kinase activity, GO:0003841 1-acylglycerol-3-phosphate O-acyltransferase activity, GO:0003867 4-aminobutyrate transaminase activity, GO:0003882 CDP-diacylglycerol-serine O-phosphatidyltransferase activity, GO:0003987 acetate-CoA ligase activity, GO:0004143 diacylglycerol kinase activity, GO:0004366 glycerol-3-phosphate O-acyltransferase activity, GO:0004368 glycerol-3-phosphate dehydrogenase (quinone) activity, GO:0004467 long-chain fatty acid-CoA ligase activity, GO:0004620 phospholipase activity, GO:0004622 lysophospholipase activity, GO:0004623 phospholipase A2 activity, GO:0004630 phospholipase D activity, GO:0005324 long-chain fatty acid transporter activity, **GO:0006071 glycerol metabolic process**, GO:0006083 acetate metabolic process, GO:0006490 oligosaccharide-lipid intermediate biosynthetic process, GO:0006629 lipid metabolic process, GO:0006631 fatty acid metabolic process, GO:0006635 fatty acid beta-oxidation, GO:0006636 unsaturated fatty acid biosynthetic process, GO:0006650 glycerophospholipid metabolic process, GO:0008444 CDP-diacylglycerol-glycerol-3-phosphate 3-phosphatidyltransferase activity, GO:0008775 acetate CoA-transferase activity, GO:0008776 acetate kinase activity, GO:0008779 acyl-[acyl-carrier-protein]-phospholipid O-acyltransferase activity, GO:0008825 cyclopropane-fatty-acyl-phospholipid synthase activity, GO:0008838 diaminopropionate ammonia-lyase activity, GO:0008888 glycerol dehydrogenase [NAD+] activity, GO:0008922 long-chain fatty acid [acyl-carrier-protein] ligase activity, GO:0008960 phosphatidylglycerol-membrane-oligosaccharide glycerophosphotransferase activity, GO:0008961 phosphatidylglycerol-prolipoprotein diacylglyceryl transferase activity, GO:0008970 phospholipase A1 activity, GO:0008980 propionate kinase activity, GO:0009062 fatty acid catabolic process, GO:0009331 glycerol-3-phosphate dehydrogenase complex, GO:0009395 phospholipid catabolic process, GO:0010124 phenylacetate catabolic process, GO:0010565 regulation of cellular ketone metabolic process, GO:0015168 glycerol transmembrane transporter activity, GO:0015169 glycerol-3-phosphate transmembrane transporter activity, GO:0015245 fatty acid transmembrane transporter activity, GO:0015254 glycerol channel activity, GO:0015430 ATPase-coupled glycerol-3-phosphate transmembrane transporter activity, GO:0015527 glycerol-phosphate:inorganic phosphate antiporter activity, GO:0015540 3-hydroxyphenyl propionate:proton symporter activity, GO:0015645 fatty acid ligase activity, GO:0015793 glycerol transport, GO:0015794 glycerol-3-phosphate transmembrane transport, GO:0015908 fatty acid transport, GO:0015909 long-chain fatty acid transport, GO:0015912 short-chain fatty acid transport, GO:0016042 lipid catabolic process, GO:0016298 lipase activity, GO:0016411 acylglycerol O-acyltransferase activity, GO:0018962 3-phenylpropionate metabolic process, **GO:0019216 regulation of lipid metabolic process**, GO:0019217 regulation of fatty acid metabolic process, GO:0019380 3-phenylpropionate catabolic process, GO:0019395 fatty acid oxidation, GO:0019413 acetate biosynthetic process, GO:0019427 acetyl-CoA biosynthetic process from acetate, GO:0019542 propionate biosynthetic process, GO:0019543 propionate catabolic process, **GO:0019563 glycerol catabolic process**, GO:0019588 anaerobic glycerol catabolic process, GO:0019622 3-(3-hydroxy)phenylpropionate catabolic process, GO:0019626 short-chain fatty acid catabolic process, GO:0019629 propionate catabolic process, 2-methylcitrate cycle, GO:0019658 glucose fermentation to lactate and acetate, GO:0019679 propionate metabolic process, methylcitrate cycle, GO:0030228 lipoprotein particle receptor activity, GO:0030258 lipid modification, GO:0031955 short-chain fatty acid-CoA ligase activity, GO:0033539 fatty acid beta-oxidation using acyl-CoA dehydrogenase, GO:0033542 fatty acid beta-oxidation, unsaturated, even number, GO:0033559 unsaturated fatty acid metabolic process, GO:0033969 gamma-glutamyl-gamma-aminobutyrate hydrolase activity, GO:0034440 lipid oxidation, GO:0035527 3-hydroxypropionate dehydrogenase (NADP+) activity, GO:0042157 lipoprotein metabolic process, GO:0042158 lipoprotein biosynthetic process, GO:0042182 ketone catabolic process, GO:0042618 poly-hydroxybutyrate metabolic process, GO:0043136 glycerol-3-phosphatase activity, GO:0043875 2-ketobutyrate formate-lyase activity, GO:0044242 cellular lipid catabolic process, GO:0044255 cellular lipid metabolic process, GO:0045017 glycerolipid biosynthetic process, GO:0045733 acetate catabolic process, GO:0045834 positive regulation of lipid metabolic process, GO:0046168 glycerol-3-phosphate catabolic process, GO:0046435 3-(3-hydroxy)phenylpropionate metabolic process, GO:0046474 glycerophospholipid biosynthetic process, GO:0046486 glycerolipid metabolic process, GO:0046834 lipid phosphorylation, GO:0047954 glycerol-2-phosphatase activity, GO:0050218 propionate-CoA ligase activity, GO:0050526 poly(3-hydroxybutyrate) depolymerase activity, GO:0051790 short-chain fatty acid biosynthetic process, GO:0052590 sn-glycerol-3-phosphate:ubiquinone oxidoreductase activity, GO:0070689 L-threonine catabolic process to propionate, GO:0070811 glycerol-2-phosphate transmembrane transport, ko00630 glyoxylate and dicarboxylate metabolism, ko00640 propanoate metabolism, **ko00660 C5-Branched dibasic acid metabolism,** ko00071 fatty acid degradation, ko00072 synthesis and degradation of ketone bodies, ko00140 steroid hormone biosynthesis, **ko00561 glycerolipid metabolism**, ko00564 glycerophospholipid metabolism, ko00565 ether lipid metabolism, ko00590 arachidonic acid metabolism, ko00591 linoleic acid metabolism, ko00592 alpha-Linolenic acid metabolism, ko01040 biosynthesis of unsaturated fatty acids |
|  | Xenobiotic detoxification | | GO:0006805 xenobiotic metabolic process, GO:0006855 drug transmembrane transport, GO:0008412 4-hydroxybenzoate octaprenyltransferase activity, GO:0008667 2,3-dihydro-2,3-dihydroxybenzoate dehydrogenase activity, GO:0008694 3-octaprenyl-4-hydroxybenzoate carboxy-lyase activity, GO:0009410 response to xenobiotic stimulus, GO:0009636 response to toxic substance, GO:0015643 toxic substance binding, GO:0015893 drug transport, GO:0017144 drug metabolic process, GO:0018966 styrene metabolic process, GO:0042178 xenobiotic catabolic process, GO:0042493 response to drug, GO:0042908 xenobiotic transport, GO:0042910 xenobiotic transmembrane transporter activity, GO:0046618 drug export, GO:0071466 cellular response to xenobiotic stimulus, GO:0097237 cellular response to toxic substance, GO:2001023 regulation of response to drug, ko00361 chlorocyclhexane and chlrbenzene degradation, **ko00362 benzoate degradation**, ko00364 fluorobenzoate degradation, **ko00621 dioxin degradation**, **ko00622 xylene degradation**, ko00623 toluene degradation, ko00624 polycyclic aromatic hydrocarbon degradation, **ko00625 chloroalkane and chloroalkene degradation**, **ko00626 vaphthalene degradation**, **ko00627 aminobenzoate degradation**, ko00633 nitrotoluene degradation, ko00642 ethylbenzene degradation, **ko00643 styrene degradation**, **ko00930 caprolactam degradation**, ko00980 metabolism of xenobiotics by cytochrome P450, ko00982 drug metabolism - cytochrome P450, ko00983 drug metabolism - other enzymes |
|  | Formic acid detoxification | | GO:0004477 methenyltetrahydrofolate cyclohydrolase activity, GO:0004486 methylenetetrahydrofolate dehydrogenase [NAD(P)+] activity, GO:0004488 methylenetetrahydrofolate dehydrogenase (NADP+) activity, GO:0005542 folic acid binding, GO:0006761 dihydrofolate biosynthetic process, GO:0008841 dihydrofolate synthase activity, GO:0009397 folic acid-containing compound catabolic process, GO:0030272 5-formyltetrahydrofolate cyclo-ligase activity, GO:0030412 formimidoyltetrahydrofolate cyclodeaminase activity, GO:0046452 dihydrofolate metabolic process, GO:0046657 folic acid catabolic process |
| Anteaters vs. Dogs | Nutrient | Non-Chitin-  Carbohydrate | KOs+GOs: GO:0000272 polysaccharide catabolic process, GO:0003850 2-deoxyglucose-6-phosphatase activity, **GO:0003978 UDP-glucose 4-epimerase activity**, GO:0003983 UTP:glucose-1-phosphate uridylyltransferase activity, GO:0004344 glucose dehydrogenase activity, GO:0004345 glucose-6-phosphate dehydrogenase activity, GO:0004346 glucose-6-phosphatase activity, GO:0004347 glucose-6-phosphate isomerase activity, **GO:0004360 glutamine-fructose-6-phosphate transaminase (isomerizing) activity**, GO:0004475 mannose-1-phosphate guanylyltransferase activity, GO:0004555 alpha,alpha-trehalase activity, GO:0004558 alpha-1,4-glucosidase activity, GO:0004614 phosphoglucomutase activity, **GO:0004616 phosphogluconate dehydrogenase (decarboxylating) activity**, GO:0004805 trehalose-phosphatase activity, **GO:0005351 carbohydrate:proton symporter activity**, GO:0005353 fructose transmembrane transporter activity, GO:0005354 galactose transmembrane transporter activity, GO:0005534 galactose binding, GO:0005537 mannose binding, GO:0005539 glycosaminoglycan binding, GO:0005984 disaccharide metabolic process, GO:0005997 xylulose metabolic process, GO:0005998 xylulose catabolic process, GO:0006109 regulation of carbohydrate metabolic process, GO:0006110 regulation of glycolytic process, GO:0008375 acetylglucosaminyltransferase activity, GO:0008446 GDP-mannose 4,6-dehydratase activity, GO:0008675 2-dehydro-3-deoxy-phosphogluconate aldolase activity, GO:0008706 6-phospho-beta-glucosidase activity, GO:0008801 beta-phosphoglucomutase activity, GO:0008873 gluconate 2-dehydrogenase activity, GO:0008874 gluconate 5-dehydrogenase activity, GO:0008875 gluconate dehydrogenase activity, GO:0008876 quinoprotein glucose dehydrogenase activity, GO:0008905 mannose-phosphate guanylyltransferase activity, GO:0008928 mannose-1-phosphate guanylyltransferase (GDP) activity, **GO:0008955 peptidoglycan glycosyltransferase activity**, GO:0008960 phosphatidylglycerol-membrane-oligosaccharide glycerophosphotransferase activity, GO:0009313 oligosaccharide catabolic process, GO:0010675 regulation of cellular carbohydrate metabolic process, GO:0010676 positive regulation of cellular carbohydrate metabolic process, GO:0010906 regulation of glucose metabolic process, GO:0015159 polysaccharide transmembrane transporter activity, GO:0015422 ATPase-coupled oligosaccharide transmembrane transporter activity, GO:0015478 oligosaccharide transporting porin activity, GO:0015517 galactose:proton symporter activity, GO:0015640 peptidoglycan peptide transmembrane transporter activity, GO:0015755 fructose transmembrane transport, GO:0015757 galactose transmembrane transport, GO:0015760 glucose-6-phosphate transport, GO:0015771 trehalose transport, GO:0015774 polysaccharide transport, GO:0015834 peptidoglycan-associated peptide transport, GO:0016137 glycoside metabolic process, GO:0016139 glycoside catabolic process, GO:0019134 glucosamine-1-phosphate N-acetyltransferase activity, GO:0019595 non-phosphorylated glucose catabolic process, GO:0019655 glycolytic fermentation to ethanol, GO:0019659 glucose catabolic process to lactate, GO:0019662 non-glycolytic fermentation, GO:0032494 response to peptidoglycan, GO:0032881 regulation of polysaccharide metabolic process, GO:0033037 polysaccharide localization, GO:0033922 peptidoglycan beta-N-acetylmuramidase activity, GO:0034069 aminoglycoside N-acetyltransferase activity, **GO:0035251 UDP-glucosyltransferase activity**, GO:0042834 peptidoglycan binding, GO:0043470 regulation of carbohydrate catabolic process, GO:0043471 regulation of cellular carbohydrate catabolic process, GO:0044247 cellular polysaccharide catabolic process, GO:0044275 cellular carbohydrate catabolic process, GO:0045013 carbon catabolite repression of transcription, GO:0045913 positive regulation of carbohydrate metabolic process, GO:0045990 carbon catabolite regulation of transcription, GO:0046323 glucose import, GO:0046352 disaccharide catabolic process, GO:0046430 non-phosphorylated glucose metabolic process, **GO:0046527 glucosyltransferase activity**, GO:0047465 N-acylglucosamine-6-phosphate 2-epimerase activity, GO:0047663 aminoglycoside 6'-N-acetyltransferase activity, GO:0047917 GDP-glucosidase activity, GO:0047919 GDP-mannose 6-dehydrogenase activity, GO:0047936 glucose 1-dehydrogenase [NAD(P)] activity, GO:0048029 monosaccharide binding, GO:0050089 mannose isomerase activity, GO:0050511 undecaprenyldiphospho-muramoylpentapeptide beta-N-acetylglucosaminyltransferase activity, GO:0050580 2,5-didehydrogluconate reductase activity, GO:0050782 galactose uniporter activity, GO:0051691 cellular oligosaccharide metabolic process, GO:0051692 cellular oligosaccharide catabolic process, GO:0051748 UTP-monosaccharide-1-phosphate uridylyltransferase activity, GO:0052751 GDP-mannose hydrolase activity, GO:0052779 amino disaccharide metabolic process, GO:0052782 amino disaccharide catabolic process, GO:0071224 cellular response to peptidoglycan, GO:0097367 carbohydrate derivative binding, GO:1901136 carbohydrate derivative catabolic process, ko00562 inositol phosphate metabolism |
|  |  |  | CAZy: Glycoside Hydrolases (GH106, GH151, GH136, GH13_29, GH43_35, **GH23**, GH13_20, GH154, **GH1**, GH78, GH128, GH13_4, GH4, GH13_39, GH156, GH159, GH112, GH13_31, GH24, GH113, GH30_1, GH129, GH13_11, **GH120**, GH43_26, GH42, GH39, GH5_8, GH5_25, GH13_21, GH81, GH43_32, GH5_44, GH43_28, GH13_3, GH5_1, GH74, GH27, GH5_36, GH13_1, GH13_33, GH49, GH93, GH143, GH50, GH5_37, GH114, GH13_16, GH157, GH165, GH147, GH82, GH5_5, GH13_27, GH87, GH105+GH28, GH116+CBM38, CBM48+GH13_9, GH13+CBM41+CBM48, GH13+CBM34, CBM48+GH13_8, GH142+GH143, GH2+CBM51+CBM67, GH2+CBM57, GH27+CBM13+CBM35, GH27+CBM51, CBM35+GH27+3.2.1.22, CBM35+CBM61+GH27+GH31, CBM35+GH39, GH43+CBM6, GH5+CBM13, GH51+CBM66, GH73+CBM50, CBM34+GH13_39+GH77,GH92+GH94, GH92+3.2.1.113, GH94+GT84, GH95+CBM13+CBM51, GH5_18, GH90, GH0+GH36, CBM57+GH2, GH37, **GH65**), GlycosylTransferases (GT2_Glyco_tranf_2_3, GT6, GT76, GT10, GT2_Glyco_trans_2_3, GT39, GT56, GT84, GT81, GT80, GT22, GT74, GT0+GT9, GT2+GT4, GT35+GT3), Polysaccharide Lyases (PL12, PL11, PL27, PL12_3, PL17_2, **PL9**, PL33_2, PL15, PL33, PL17, L9_2, PL4_4, PL6_1, PL7), Carbohydrate Esterases(CE15, CE13), Auxiliary Activities (AA4, AA6, AA3_2, AA3), Carbohydrate-Binding Modules (CBM13, CBM48, **CBM34**, CBM50, CBM61, CBM26, CBM35, CBM57, CBM74, CBM2, CBM38, CBM16, CBM58, CBM83, CBM82, CBM3), Cellulosome (SLH) |
|  |  | Chitin | KOs+GOs: GO:0003977 UDP-N-acetylglucosamine diphosphorylase activity, GO:0004568 chitinase activity, GO:0006030 chitin metabolic process, GO:0006032 chitin catabolic process, GO:0008761 UDP-N-acetylglucosamine 2-epimerase activity, GO:0008843 endochitinase activity, GO:0036311 chitin disaccharide deacetylase activity, GO:0045127 N-acetylglucosamine kinase activity, GO:0052777 diacetylchitobiose catabolic process, GO:0052778 diacetylchitobiose metabolic process |
|  |  |  | CAZy: chitinase (GH18, CBM5+GH18, GH18+ CBM5, CBM5+GH19), chitin deacetylase (CE4, CE4+GH153), chitin-binding modules (CBM50, CBM5+CBM50, CBM54), exo-beta-glucosaminidase (GH9) |
|  |  | Protein | GO:0000096 sulfur amino acid metabolic process, GO:0003958 NADPH-hemoprotein reductase activity, GO:0004174 electron-transferring-flavoprotein dehydrogenase activity, GO:0004176 ATP-dependent peptidase activity, GO:0004721 phosphoprotein phosphatase activity, GO:0004722 protein serine/threonine phosphatase activity, GO:0006468 protein phosphorylation, **GO:0006522 alanine metabolic process**, GO:0006530 asparagine catabolic process, **GO:0006534 cysteine metabolic process**, GO:0006544 glycine metabolic process, GO:0006552 leucine catabolic process, **GO:0006554 lysine catabolic process**, **GO:0006558 L-phenylalanine metabolic process**, GO:0006562 proline catabolic process, GO:0006563 L-serine metabolic process, **GO:0006570 tyrosine metabolic process**, **GO:0006573 valine metabolic process**, GO:0006593 ornithine catabolic process, GO:0006613 cotranslational protein targeting to membrane, GO:0006614 SRP-dependent cotranslational protein targeting to membrane, GO:0008092 cytoskeletal protein binding, GO:0008113 peptide-methionine (S)-S-oxide reductase activity, GO:0008138 protein tyrosine/serine/threonine phosphatase activity, GO:0008242 omega peptidase activity, GO:0008770 [acyl-carrier-protein] phosphodiesterase activity, GO:0008798 beta-aspartyl-peptidase activity, GO:0008876 quinoprotein glucose dehydrogenase activity, GO:0009046 zinc D-Ala-D-Ala carboxypeptidase activity, GO:0009069 serine family amino acid metabolic process, **GO:0009078 pyruvate family amino acid metabolic process**, GO:0009305 protein biotinylation, GO:0010133 proline catabolic process to glutamate, GO:0016579 protein deubiquitination, GO:0016805 dipeptidase activity, GO:0018126 protein hydroxylation, **GO:0018249 protein dehydration**, GO:0018298 protein-chromophore linkage, GO:0019243 methylglyoxal catabolic process to D-lactate via S-lactoyl-glutathione, GO:0019466 ornithine catabolic process via proline, **GO:0019477 L-lysine catabolic process**, GO:0019478 D-amino acid catabolic process, GO:0019529 taurine catabolic process, GO:0019530 taurine metabolic process, GO:0019665 anaerobic amino acid catabolic process, GO:0023014 signal transduction by protein phosphorylation, GO:0030163 protein catabolic process, GO:0031455 glycine betaine metabolic process, GO:0032271 regulation of protein polymerization, GO:0033345 asparagine catabolic process via L-aspartate, GO:0033743 peptide-methionine (R)-S-oxide reductase activity, GO:0034017 trans-2-decenoyl-acyl-carrier-protein isomerase activity, GO:0035268 protein mannosylation, GO:0035269 protein O-linked mannosylation, GO:0035304 regulation of protein dephosphorylation, GO:0035307 positive regulation of protein dephosphorylation, GO:0043393 regulation of protein binding, GO:0045047 protein targeting to ER, **GO:0046144 D-alanine family amino acid metabolic process**, **GO:0046416 D-amino acid metabolic process**, **GO:0046436 D-alanine metabolic process**, **GO:0046440 L-lysine metabolic process**, GO:0046777 protein autophosphorylation, GO:0046944 protein carbamoylation, GO:0051223 regulation of protein transport, GO:0051258 protein polymerization, GO:0052868 protein-lysine lysyltransferase activity, GO:0070006 metalloaminopeptidase activity, GO:0070084 protein initiator methionine removal, GO:0071915 protein-lysine lysylation, **GO:1902221 erythrose 4-phosphate/phosphoenolpyruvate family amino acid metabolic process, ko00310 lysine degradation**, ko00340 histidine metabolism, **ko00380 tryptophan metabolism**, **ko00450 selenocompound metabolism**, ko00472 D-Arginine and D-ornithine metabolism, ko00473 D-Alanine metabolism, **ko00480 glutathione metabolism**, ko03050 Proteasome |
|  |  | Lipid | GO:0001727 lipid kinase activity, GO:0003867 4-aminobutyrate transaminase activity, GO:0003987 acetate-CoA ligase activity, GO:0004424 imidazoleglycerol-phosphate dehydratase activity, GO:0004630 phospholipase D activity, GO:0004806 triglyceride lipase activity, GO:0005324 long-chain fatty acid transporter activity, **GO:0006071 glycerol metabolic process**, GO:0006083 acetate metabolic process, GO:0006490 oligosaccharide-lipid intermediate biosynthetic process, GO:0006636 unsaturated fatty acid biosynthetic process, GO:0008444 CDP-diacylglycerol-glycerol-3-phosphate 3-phosphatidyltransferase activity, GO:0008679 2-hydroxy-3-oxopropionate reductase activity, GO:0008775 acetate CoA-transferase activity, GO:0008825 cyclopropane-fatty-acyl-phospholipid synthase activity, GO:0008838 diaminopropionate ammonia-lyase activity, GO:0008888 glycerol dehydrogenase [NAD+] activity, GO:0008960 phosphatidylglycerol-membrane-oligosaccharide glycerophosphotransferase activity, GO:0009331 glycerol-3-phosphate dehydrogenase complex, GO:0010565 regulation of cellular ketone metabolic process, GO:0015168 glycerol transmembrane transporter activity, GO:0015245 fatty acid transmembrane transporter activity, GO:0015254 glycerol channel activity, GO:0015527 glycerol-phosphate:inorganic phosphate antiporter activity, GO:0015540 3-hydroxyphenyl propionate:proton symporter activity, GO:0015793 glycerol transport, GO:0015908 fatty acid transport, GO:0015909 long-chain fatty acid transport, GO:0015912 short-chain fatty acid transport, GO:0016298 lipase activity, GO:0018962 3-phenylpropionate metabolic process, **GO:0019216 regulation of lipid metabolic process**, GO:0019217 regulation of fatty acid metabolic process, GO:0019380 3-phenylpropionate catabolic process, GO:0019413 acetate biosynthetic process, GO:0019415 acetate biosynthetic process from carbon monoxide, GO:0019427 acetyl-CoA biosynthetic process from acetate, **GO:0019563 glycerol catabolic process**, GO:0019588 anaerobic glycerol catabolic process, GO:0019622 3-(3-hydroxy)phenylpropionate catabolic process, GO:0019658 glucose fermentation to lactate and acetate, GO:0019679 propionate metabolic process, methylcitrate cycle, GO:0030228 lipoprotein particle receptor activity, GO:0031955 short-chain fatty acid-CoA ligase activity, GO:0033539 fatty acid beta-oxidation using acyl-CoA dehydrogenase, GO:0033542 fatty acid beta-oxidation, unsaturated, even number, GO:0033559 unsaturated fatty acid metabolic process, GO:0042618 poly-hydroxybutyrate metabolic process, GO:0043136 glycerol-3-phosphatase activity, GO:0045733 acetate catabolic process, GO:0045834 positive regulation of lipid metabolic process, GO:0046168 glycerol-3-phosphate catabolic process, GO:0046435 3-(3-hydroxy)phenylpropionate metabolic process, GO:0046834 lipid phosphorylation, GO:0046890 regulation of lipid biosynthetic process, GO:0047577 4-hydroxybutyrate dehydrogenase activity, GO:0050218 propionate-CoA ligase activity, GO:0052590 sn-glycerol-3-phosphate:ubiquinone oxidoreductase activity, ko00062 Fatty acid elongation, **ko00072 Synthesis and degradation of ketone bodies**, ko00100 Steroid biosynthesis, ko00120 Primary bile acid biosynthesis, **ko00561 glycerolipid metabolism**, ko01040 Biosynthesis of unsaturated fatty acids |
|  | Xenobiotic detoxification | | GO:0006805 xenobiotic metabolic process, GO:0008412 4-hydroxybenzoate octaprenyltransferase activity, GO:0008667 2,3-dihydro-2,3-dihydroxybenzoate dehydrogenase activity, GO:0008694 3-octaprenyl-4-hydroxybenzoate carboxy-lyase activity, GO:0009410 response to xenobiotic stimulus, GO:0018966 styrene metabolic process, GO:0071466 cellular response to xenobiotic stimulus, GO:0097237 cellular response to toxic substance, GO:2001023 regulation of response to drug, **ko00362 benzoate degradation**, ko00365 furfural degradation, **ko00621 dioxin degradation**, **ko00622 xylene degradation**, **ko00625 chloroalkane and chloroalkene degradation**, **ko00626 vaphthalene degradation**, **ko00627 aminobenzoate degradation**, ko00642 ethylbenzene degradation, **ko00643 styrene degradation**, **ko00930 caprolactam degradation**, ko00983 drug metabolism - other enzymes |
|  | Formic acid detoxification | | GO:0004477 methenyltetrahydrofolate cyclohydrolase activity, GO:0030272 5-formyltetrahydrofolate cyclo-ligase activity, GO:0030412 formimidoyltetrahydrofolate cyclodeaminase activity |
| Echidna vs. Other 38 mammals | Nutrient | Non-Chitin-  Carbohydrate | KOs+GOs: **GO:0003978 UDP-glucose 4-epimerase activity**, **GO:0004360 glutamine-fructose-6-phosphate transaminase (isomerizing) activity**, GO:0004614 phosphoglucomutase activity, **GO:0004616 phosphogluconate dehydrogenase (decarboxylating) activity**, **GO:0005351 carbohydrate:proton symporter activity**, GO:0005402 carbohydrate:cation symporter activity, GO:0006012 galactose metabolic process, GO:0006026 aminoglycan catabolic process, GO:0006027 glycosaminoglycan catabolic process, GO:0006098 pentose-phosphate shunt, **GO:0008955 peptidoglycan glycosyltransferase activity**, GO:0009029 tetraacyldisaccharide 4'-kinase activity, GO:0009253 peptidoglycan catabolic process, GO:0009254 peptidoglycan turnover, GO:0009273 peptidoglycan-based cell wall biogenesis, GO:0009274 peptidoglycan-based cell wall, GO:0019388 galactose catabolic process, GO:0030604 1-deoxy-D-xylulose-5-phosphate reductoisomerase activity, GO:0033499 galactose catabolic process via UDP-galactose, **GO:0035251 UDP-glucosyltransferase activity**, GO:0046401 lipopolysaccharide core region metabolic process, **GO:0046527 glucosyltransferase activity**, GO:0071972 peptidoglycan L,D-transpeptidase activity, GO:1901135 carbohydrate derivative metabolic process, KEGG: ko00010 glycolysis / Gluconeogenesis, ko00030 pentose phosphate pathway, ko00620 pyruvate metabolism |
|  |  |  | CAZy: Glycoside Hydrolases (**GH65**, **GH1**, GH105+GH28, GH113, **GH120**, GH126, GH13, GH13+CBM34, GH13+CBM41, GH13+GH77+CBM34, GH144, GH16, **GH23**, GH23+CBM50, GH25, GH25+CBM50, GH25+GH73+CBM50, GH32+CBM66, GH33+CBM32, GH43+CBM42, GH53, GH70, GH73, GH73+CBM50, GH74+CBM1+CBM2, GH74+CBM2, GH85, GH92+GH94), GlycosylTransferases (GT0, GT1, GT101, GT14, GT19, GT2, GT20, GT26, GT2+GT4, GT4, GT51, GT8), Polysaccharide Lyases (PL11+CBM2, **PL9**), Carbohydrate Esterases(CE14), Carbohydrate-Binding Modules (CBM2, **CBM34**, CBM38, CBM41) |
|  |  | Chitin | KOs+GOs: GO:0006047 UDP-N-acetylglucosamine metabolic process |
|  |  |  | CAZy: chitinase (GH18+CBM12+CBM5), lytic chitin monooxygenase (AA10), exo-beta-glucosaminidase (GH9+CBM2+CBM4, CE0+GH9) |
|  |  | Protein | GO:0003756 protein disulfide isomerase activity, GO:0004180 carboxypeptidase activity, GO:0004185 serine-type carboxypeptidase activity, GO:0004222 metalloendopeptidase activity, GO:0004314 [acyl-carrier-protein] S-malonyltransferase activity, GO:0004318 enoyl-[acyl-carrier-protein] reductase (NADH) activity, GO:0005515 protein binding, **GO:0006522 alanine metabolic process**, GO:0006527 arginine catabolic process, GO:0006530 asparagine catabolic process, **GO:0006534 cysteine metabolic process**, GO:0006541 glutamine metabolic process, GO:0006543 glutamine catabolic process, GO:0006546 glycine catabolic process, GO:0006548 histidine catabolic process, **GO:0006554 lysine catabolic process**, **GO:0006558 L-phenylalanine metabolic process**, GO:0006560 proline metabolic process, GO:0006568 tryptophan metabolic process, **GO:0006570 tyrosine metabolic process**, **GO:0006573 valine metabolic process**, GO:0006605 protein targeting, GO:0006612 protein targeting to membrane, GO:0006613 cotranslational protein targeting to membrane, GO:0006614 SRP-dependent cotranslational protein targeting to membrane, GO:0006886 intracellular protein transport, GO:0008213 protein alkylation, GO:0008237 metallopeptidase activity, GO:0009002 serine-type D-Ala-D-Ala carboxypeptidase activity, GO:0009063 cellular amino acid catabolic process, GO:0009065 glutamine family amino acid catabolic process, GO:0009072 aromatic amino acid family metabolic process, **GO:0009078 pyruvate family amino acid metabolic process**, GO:0009083 branched-chain amino acid catabolic process, GO:0015031 protein transport, GO:0016485 protein processing, GO:0018101 protein citrullination, **GO:0018249 protein dehydration**, **GO:0019477 L-lysine catabolic process**, GO:0019538 protein metabolic process, GO:0031522 cell envelope Sec protein transport complex, GO:0032991 protein-containing complex, GO:0033345 asparagine catabolic process via L-aspartate, GO:0043933 protein-containing complex subunit organization, GO:0043952 protein transport by the Sec complex, GO:0044267 cellular protein metabolic process, GO:0045047 protein targeting to ER, **GO:0046144 D-alanine family amino acid metabolic process**, **GO:0046416 D-amino acid metabolic process**, **GO:0046436 D-alanine metabolic process**, **GO:0046440 L-lysine metabolic process**, GO:0046983 protein dimerization activity, GO:0051258 protein polymerization, GO:0065002 intracellular protein transmembrane transport, GO:0070008 serine-type exopeptidase activity, GO:0070930 trans-translation-dependent protein tagging, GO:0071806 protein transmembrane transport, GO:1901606 alpha-amino acid catabolic process, **GO:1902221 erythrose 4-phosphate/phosphoenolpyruvate family amino acid metabolic process,** ko00280 valine, leucine and isoleucine degradation, ko00300 lysine biosynthesis, **ko00310 lysine degradation**, ko00350 tyrosine metabolism, **ko00380 tryptophan metabolism**, ko00430 taurine and hypotaurine metabolism, **ko0450** s**elenocompound** **metabolism**, ko00473 D-Alanine metabolism, **ko00480 glutathione metabolism** |
|  |  | Lipid | GO:0004367 glycerol-3-phosphate dehydrogenase [NAD+] activity, GO:0004424 imidazoleglycerol-phosphate dehydratase activity, **GO:0006071 glycerol metabolic process**, GO:0006072 glycerol-3-phosphate metabolic process, GO:0006629 lipid metabolic process, GO:0006644 phospholipid metabolic process, GO:0008610 lipid biosynthetic process, GO:0008654 phospholipid biosynthetic process, GO:0008679 2-hydroxy-3-oxopropionate reductase activity, GO:0010124 phenylacetate catabolic process, **GO:0019216 regulation of lipid metabolic process**, GO:0019541 propionate metabolic process, **GO:0019563 glycerol catabolic process**, GO:0019678 propionate metabolic process, methylmalonyl pathway, GO:0042180 cellular ketone metabolic process, GO:0042181 ketone biosynthetic process, GO:0044255 cellular lipid metabolic process, GO:0046459 short-chain fatty acid metabolic process, GO:0046890 regulation of lipid biosynthetic process, GO:0047475 phenylacetate-CoA ligase activity, GO:0047952 glycerol-3-phosphate dehydrogenase [NAD(P)+] activity, ko00640 Propanoate metabolism, ko00650 butanoate metabolism, **ko00660 C5-Branched dibasic acid metabolism**, ko00061 fatty acid biosynthesis, ko00071 fatty acid degradation, **ko00072 synthesis and degradation of ketone bodies**, ko00120 primary bile acid biosynthesis, ko00121 secondary bile acid biosynthesis, ko00140 steroid hormone biosynthesis [PATH:ko00140], **ko00561 glycerolipid metabolism**, ko00590 arachidonic acid metabolism, ko01040 biosynthesis of unsaturated fatty acids, ko00984 Steroid degradation |
|  | Xenobiotic detoxification | | GO:0008144 drug binding, GO:0042178 BaP--xenobiotic catabolic process, ko00361 chlorocyclhexane and chlrbenzene degradation, ko00362 benzoate degradation, ko00363 Bisphenol degradation, ko00364 fluorobenzoate degradation, ko00621 dioxin degradation, ko00622 xylene degradation, ko00623 toluene degradation, ko00624 polycyclic aromatic hydrocarbon degradation, ko00625 chloroalkane and chloroalkene degradation, ko00626 vaphthalene degradation, ko00627 aminobenzoate degradation, ko00643 styrene degradation, ko00930 caprolactam degradation, ko00980 metabolism of xenobiotics by cytochrome P450, ko00982 drug metabolism - cytochrome P450 |
|  | Formic acid detoxification | | GO:0009396 folic acid-containing compound biosynthetic process, GO:0004489 methylenetetrahydrofolate reductase (NAD(P)H) activity, ko00790 folate biosynthesis |

**Table S14.** **Enzyme activity assays in the pangolins and dogs.** “-” indicates that the sample was not successfully collected.

| **Species** | **Sample ID** | **Tissues/Contents** | **Chitinolytic Activity (U/g)** | | **Trehalase Activity (U/g)** |
| --- | --- | --- | --- | --- | --- |
| Malayan pangolin (*Manis javanica*) | Mp01T | Stomach tissue | 0.21 | 9.50 | |
|  |  | Small Intestine tissue | 0.07 | 87.06 | |
|  |  | Colon tissue | 0.14 | 11.67 | |
|  |  | Stomach content | 5.24 | 35.07 | |
|  |  | Small Intestine content | 0.75 | 208.15 | |
|  |  | Colon content | 5.5 | 14.74 | |
|  | Mp02T | Stomach tissue | 0.04 | 10.79 | |
|  |  | Small Intestine tissue | 0.04 | 72.49 | |
|  |  | Colon tissue | 0.3 | 11.25 | |
|  |  | Stomach content | - | - | |
|  |  | Small Intestine content | 0.2 | 49.82 | |
|  |  | Colon content | 6.8 | 239.35 | |
|  | Mp03T | Oxyntic Gland tissue | 45.97 | 4.52 | |
|  |  | Small Intestine tissue | 0.19 | 53.52 | |
|  |  | Colon tissue | 0.17 | 9.68 | |
|  |  | Stomach content | 4.22 | 38.25 | |
|  |  | Small Intestine content | 0.59 | 96.16 | |
|  |  | Colon content | 5.65 | 33.45 | |
| Dog  (*Canis familiaris*) | DA | Stomach tissue | 0.2 | 8.00 | |
|  |  | Small Intestine tissue | 0.06 | 7.65 | |
|  |  | Colon tissue | 0.06 | 8.77 | |
|  |  | Stomach content | 0 | 32.38 | |
|  |  | Small Intestine content | 1.12 | 33.79 | |
|  |  | Colon content | 0.53 | 8.51 | |

**Table S15. Summary of the metagenomics data from the digestive tract contents of the pangolin.**

| **Sample ID** | **contents** | **Clean Data (Mb)** | **Contigs** | **ORFs** | **Mapping rate (%)** |
| --- | --- | --- | --- | --- | --- |
| Mp02T | Stomach | 453.49 | 24,436 | 35,866 | 39.37 |
|  | Small Intestine | 494.52 | 31,129 | 38,549 | 27.17 |
|  | Colon | 2,566.15 | 68,695 | 140,496 | 81.92 |

Table S16. Classification of chitin-degrading related function at high abundance in the digestive tract contents of pangolin.

| Content | GO and KEGG and CAZy |
| --- | --- |
| Stomach | GOs: GO:0008061 chitin binding  KOAs: K03933 chitin-binding protein  CAZy: lytic chitin monooxygenase (AA10), trehalase (GH65), β-hexosaminidase (GH20+CBM5) |
| Small Intestine | CAZy: chitosanase (GH8), chitinase (CBM5+GH18), β-hexosaminidase (GH20+3.2.1.52) |
| Colon | GOs: GO:0004568 chitinase activity, GO:0006030 chitin metabolic process, GO:0006032 chitin catabolic process, GO:0008843 endochitinase activity, GO:0036311 chitin disaccharide deacetylase activity, GO:0016977 chitosanase activity, GO:0052777 diacetylchitobiose catabolic process, GO:0052778 diacetylchitobiose metabolic process  KOAs: K01183 chitinase [EC:3.2.1.14], K13381 bifunctional chitinase/lysozyme [EC:3.2.1.14 3.2.1.17], K03478 chitin disaccharide deacetylase [EC:3.5.1.105], K0452 chitin deacetylase [EC:3.5.1.41], K03791 putative chitinase  CAZy: endoglucanase (GH9), chitinase (GH18, GH19, CBM5+GH19, CBM5+GH18+3.2.1.14, GH48+CBM2), chitin deacetylase (CE4, CE4+GH153), chitin-binding modules (CBM5, CBM50), β-hexosaminidase (GH20) |

**Table S17. List of genes and primer pairs used for quantitative reverse transcription PCR (RT-qPCR).**

| **Species** |  | **Gene** |  | **Primer sequence (5'->3')** |
| --- | --- | --- | --- | --- |
| Malayan pangolin (*Manis javanica*) | Target gene | *CHIA* (Acidic Mammalian Chitinase) | F | GGACGCTTCATGCCTAGTAACA |
|  |  |  | R | GCAGTTCCAAAGTTCCATCCTC |
|  |  | *AMDHD2* (Amidohydrolase Domain Containing 2) | F | TGGTGGCTCGGAGGTTATTG |
|  |  |  | R | CGCTTCTCCCGGCTGATAAA |
|  |  | *GNPDA1* (Glucosamine-6-Phosphate Deaminase 1) | F | ATTGACATCCACCCAGAAA |
|  |  |  | R | ATGCCTCCGACAAACAGC |
|  |  | *TREH* (Trehalase) | F | AATGAATCCCAAGCCACG |
|  |  |  | R | ATTTCAAAGCCTTGTCCACG |
|  | Reference gene | *TBP* (TATA-Box Binding Protein) | F | CTTGGCTGTAAACTTGACCT |
|  |  |  | R | GTCTGGACTGTTCTTCACTCTT |
| Dog (*Canis familiaris*) | Target gene | *CHIA* (Acidic Mammalian Chitinase) | F | CAATGAGTGGGTTGGCTATG |
|  |  |  | R | AAGGGCTTTCTTCAGGGTG |
|  |  | *AMDHD2* (Amidohydrolase Domain Containing 2) | F | TTTCCACCACCGTGACCC |
|  |  |  | R | GCCCATCTACTTCCACCTCC |
|  |  | *GNPDA1* (Glucosamine-6-Phosphate Deaminase 1) | F | TGTCAGGGAGGTGCTGTTGT |
|  |  |  | R | CTGGGTTGAACTGGATGATGC |
|  |  | *TREH* (Trehalase) | F | GAGGAGAAAGGTGCCTGGTT |
|  |  |  | R | GTTCTGGAGAGAGGTCGGGA |
|  | Reference gene | *TBP* (TATA-Box Binding Protein) | F | CTTGGCTGTAAACTTGACCT |
|  |  |  | R | GTCTGGACTGTTCTTCACTCTT |

**REFERENCES**

1. Akinwole MT and Babarinde IA. Assessing Tissue Lysis with Sodium Dodecyl Sulphate for DNA Extraction from Frozen Animal Tissue. *J Forensic Res* 2019; **10**: 3.
2. Chin CS, Alexander DH, Marks P et al. Nonhybrid, finished microbial genome assemblies from long-read SMRT sequencing data. *Nature Methods* 2013; **10**: 563-569.
3. Walker BJ, Abeel T, SheaT et al. Pilon: An Integrated Tool for Comprehensive Microbial Variant Detection and Genome Assembly Improvement. *PLoS ONE* 2014; **9**:e112963.
4. Parra G, Bradnam K, Korf et al. CEGMA. *Bioinformatics* 2007; **23**: 1061-7.
5. Simão F, Waterhouse RM, Panagiotis I et al. BUSCO: Assessing genome assembly and annotation completeness with single-copy orthologs. *Bioinformatics* 2015; **31**: 3210-2.
6. Li H and Durbin R. Fast and accurate short read alignment with Burrows-Wheeler transform. *Bioinformatics* 2009; **25**: 1754-60.
7. Mckenna A, Hanna M, Banks E, et al. The Genome Analysis Toolkit: A MapReduce framework for analyzing next-generation DNA sequencing data. *Genome Res* 2010; **20**: 1297-1303.
8. Tarailo-Graovac M and Chen N. Using RepeatMasker to identify repetitive elements in genomic sequences. *Curr Protoc Bioinformatics* 2009; **Chapter 4**: Unit 4.10.
9. Jurka J, Kapitonov VV, Pavlicek A et al. Repbase Update, a database of eukaryotic repetitive elements. *Cytogenet Genome Res* 2005; **110**: 462-7.
10. Benson G. Tandem repeats finder: A program to analyze DNA sequences. *Nucleic Acids Res* 1999; **27**: 573-80.
11. Stanke M, Steinkamp R, Waack S et al. AUGUSTUS: a web server for gene finding in eukaryotes. *Nucleic Acids Res* 2004; **32**: W309-12.
12. Majoros W, Pertea M, and Salzberg S. TigrScan and GlimmerHMM: two open source ab initio eukaryotic gene-finders. *Bioinformatics* 2004; **20**: 2878-9.
13. Blanco E, Parra G, and Guigó R. Using geneid to identify genes. *Curr Protoc Bioinformatics* 2007; **Chapter 4**: Unit 4.3.
14. Birney E and Durbin R. Using GeneWise in the Drosophila annotation experiment. *Genome Res* 2000; **10**: 547-8.
15. Haas BJ, Salzberg SL, Wei Z et al. Automated eukaryotic gene structure annotation using EVidenceModeler and the Program to Assemble Spliced Alignments. *Genome Biol* 2008; **9**: R7.
16. Boeckmann B, Bairoch A, Apweiler R et al. The SWISS-PROT protein knowledgebase and its supplement TrEMBL in 2003. *Nucleic Acids Res* 2003; **31**: 365-70.
17. Gerlich M and Neumann S. KEGG: Kyoto encyclopedia of genes and genomes. *Nucleic Acids Res* 2000; **28**: 27-30.
18. Dimmer EC, Huntley RP, Alam-Faruque Y et al. The UniProt-GO Annotation database in 2011. *Nucleic Acids Res* 2012; **40**: D565-70.
19. Moreno-Hagelsieb G and Latimer K. Choosing BLAST options for better detection of orthologs as reciprocal best hits. *Bioinformatics* 2007; **24**: 319-24.
20. Zdobnov EM and Rolf A. InterProScan--an integration platform for the signature-recognition methods in InterPro. *Bioinformatics* 2001; **17**: 847-8.
21. Sarah H, Rolf A, Attwood TK et al. InterPro: The integrative protein signature database. *Nucleic Acids Res* 2009; **37**: D211-5.
22. Ruan J, Li H, Chen Z et al. TreeFam: 2008 Update. *Nucleic Acids Res* 2008; **36**: D735-40.
23. Bie TD, Cristianini N, Demuth JP et al. CAFE: A computational tool for the study of gene family evolution. *Bioinformatics* 2006; **22**: 1269-71.
24. Stamatakis A. Using RAxML to infer phylogenies. *Curr Protoc Bioinformatics* 2015; **51**: 6.14.1-6.14.14.
25. Darriba D, Taboada GL, Doallo R et al. jModelTest 2: More models, new heuristics and parallel computing. *Nature Methods* 2012; **9**: 772.
26. Löytynoja A. Phylogeny-aware alignment with PRANK. *Methods Mol Biol* 2014; **1079**: 155-70.
27. Yang Z. PAML 4: Phylogenetic Analysis by Maximum Likelihood. *Mol Biol Evol* 2007; **24**: 1586-91.
28. Hedges SB, Dudley J, Kumar et al. TimeTree: A public knowledge-base of divergence times among organisms. *Bioinformatics* 2006; **22**: 2971-2.
29. Liu L, Zhang J, Rheindt FE et al. Genomic evidence reveals a radiation of placental mammals uninterrupted by the KPg boundary. *Proc Natl Acad Sci U S A* 2017; **114**: E7282-90.
30. Wesley‐Hunt GD and Flynn JJ. Phylogeny of the Carnivora: Basal relationships among the carnivoramorphans, and assessment of the position of ‘Miacoidea’relative to Carnivora. *J Syst Palaeonto* 2005; **3**: 1-28.
31. Meredith RW, Janečka JE, Gatesy J et al. Impacts of the Cretaceous Terrestrial Revolution and KPg extinction on mammal diversification. *Science* 2011; **334**: 521-4.
32. Springer MS, Emerling CA, Meredith RW et al. Waking the undead: Implications of a soft explosive model for the timing of placental mammal diversification. *Mol Phylogenet Evol* 2017; **106**: 86-102.
33. Sonnhammer EL and Ostlund G. InParanoid 8: Orthology analysis between 273 proteomes, mostly eukaryotic. *Nucleic Acids Res* 2015; **43**: D234-9.
34. Zhang J, Rasmus N, and Yang Z. Evaluation of an improved branch-site likelihood method for detecting positive selection at the molecular level. *Mol Biol Evol* 2005; **22**: 2472-9.
35. Zou Z and Zhang J. Are convergent and parallel amino acid substitutions in protein evolution more prevalent than neutral expectations? *Mol Biol Evol* 2015; **32**: 2085-96.
36. Zhou X, Xu S, Xu J et al. Phylogenomic analysis resolves the interordinal relationships and rapid diversification of the laurasiatherian mammals. *Syst Biol* 2012; **61**: 150-64.
37. Esselstyn JA, Oliveros CH, Swanson MT et al. Investigating difficult nodes in the placental mammal tree with expanded taxon sampling and thousands of ultraconserved elements. *Genome Biol Evol* 2017; **9**: 2308-21.
38. Lv X, Hu JY, Hu YW et al. Diverse phylogenomic datasets uncover a concordant scenario of laurasiatherian interordinal relationships. *Mol Phylogenet Evol* 2021; **157**: 107065.
39. Ma JE, Li LM, Jiang HY et al. Acidic mammalian chitinase gene is highly expressed in the special oxyntic glands of Manis javanica. *FEBS Open Bio* 2018; **8**: 1247-55.
40. Yong K. Btrim: A fast, lightweight adapter and quality trimming program for next-generation sequencing technologies. *Genomics* 2011; **98**: 152-3.
41. Bolger AM, Lohse M, and Usadel B. Trimmomatic: A flexible trimmer for Illumina sequence data. *Bioinformatics* 2014; **30**: 2114-20.
42. Hu JY, Hao ZQ, Laurent F et al. Genomic consequences of population decline in critically endangered pangolins and their demographic histories. *Natl Sci Rev* 2020; **7**: 84-100.
43. Pertea M, Kim D, Pertea GM et al. Transcript-level expression analysis of RNA-seq experiments with HISAT, StringTie and Ballgown. *Nature Protoc* 2016; **11**: 1650-67.
44. Chen C, Zhou Y, Fu H et al. Expanded catalog of microbial genes and metagenome-assembled genomes from the pig gut microbiome. *Nature Commun* 2021; **12**: 1106.
45. Hauke J and Kossowski T. Comparison of values of Pearson's and Spearman's correlation coefficients on the same sets of data. *Quaest Geograph* 2011; **30**: 87-93.
46. Love MI, Huber W, and Anders S. Moderated estimation of fold change and dispersion for RNA-seq data with DESeq2. *Genome Biol* 2014; **15**: 550.
47. Smyth GK. edgeR: a Bioconductor package for differential expression analysis of digital gene expression data. *Bioinformatics* 2010; **26**: 139-40.
48. Benjamini Y and Hochberg Y. Controlling the False Discovery Rate: A practical and powerful approach to multiple testing. *J R Stat Soc B* 1995; **57**: 289-300.
49. Storey JD and Tibshirani R. Statistical significance for genomewide studies. *Proc Natl Acad Sci U S A* 2003; **100**: 9440-5.
50. Coelho LP, Kultima JR, Costea PI et al. Similarity of the dog and human gut microbiomes in gene content and response to diet. *Microbiome* 2018; **6**: 72.
51. Li D, Liu CM, Luo R et al. MEGAHIT: An ultra-fast single-node solution for large and complex metagenomics assembly via succinct de Bruijn graph. *Bioinformatics* 2015; **31**: 1674-6.
52. Zhu W, Lomsadze A, and Borodovsky M. Ab initio gene identification in metagenomic sequences. *Nucleic Acids Res* 2010; **38**: e132.
53. Li min, Niu, Bei fang et al. CD-HIT: accelerated for clustering the next-generation sequencing data. *Bioinformatics* 2012; **28**: 3150-2.
54. Buchfink B, Xie C, and Huson DH. Fast and sensitive protein alignment using DIAMOND. *Nature Methods* 2015; **12**: 59-60.
55. Huson DH, Beier S, Flade I et al. MEGAN Community Edition - Interactive exploration and analysis of large-scale microbiome sequencing data. *PLoS Comput Biol* 2016; **12**: e1004957.
56. Johnson LS, Eddy SR, and Portugaly E. Hidden Markov model speed heuristic and iterative HMM search procedure. *BMC Bioinformatics* 2010; **11**: 431.
57. Jaime HC, Kristoffer F, Pedro CL et al. Fast genome-wide functional annotation through orthology assignment by eggNOG-Mapper. *Mol Biol Evol* 2016; **34**: 2115.
58. Huerta-Cepas J, Szklarczyk D, Heller D et al. eggNOG 5.0: A hierarchical, functionally and phylogenetically annotated orthology resource based on 5090 organisms and 2502 viruses. *Nucleic Acids Rese* 2019; **47**: D309-14.
59. Zhang H, Tanner Y, Huang L et al. dbCAN2: A meta server for automated carbohydrate-active enzyme annotation. *Nucleic Acids Res* 2018; **46**: W95-W101.
60. Willis ATJ. Canonical analysis of Principal Coordinates: A useful method of constrained ordination for ecology. *Ecology* 2003; **84**: 511-25.
61. Dixon P. VEGAN, a package of R functions for community ecology. *J Veg Sci*. 2003; **14**: 927-30.
62. Muegge BD, Kuczynski J, Dan K et al. Diet drives convergence in gut microbiome functions across mammalian phylogeny and within humans. *Science* 2011; **332**: 970.
63. Miller GL. Use of dinitrosalicylic acid reagent for determination of reducing sugar. *Anal Chem* 1959; **31**: 426-8.
64. Coelho L, Kultima J, Costea P et al. Similarity of the dog and human gut microbiomes in gene content and response to diet. *Microbiome* 2018; **6**: 72.
